# Programmable Assembly of Mechanically Robust and Functional Polymer–Spore Biocomposites in Organic Solvent

**DOI:** 10.1021/jacs.5c13976

**Published:** 2025-10-21

**Authors:** Masamu Kawada, Ziyu Cui, Justin Chen, Reis Dorit, Jaeho Cho, Seunghyun Sim

**Affiliations:** † Department of Chemistry, 8788 University of California Irvine, Irvine, California 92697, United States; ‡ Department of Chemical and Biomolecular Engineering, 8788 University of California Irvine, Irvine, California 92697, United States; § Department of Biomedical Engineering, University of California Irvine, Irvine, California 92697, United States; ∥ Center for Complex and Active Materials, University of California Irvine, Irvine, California 92697, United States

## Abstract

Microbial biocomposites offer genetically programmable and regenerative functionality, but their mechanical tunability remains limited by the mild conditions required for biological activity and viability. Here, we report the programmable self-assembly of *Bacillus subtilis* spores with benzalcyanoacetate (BCA)-functionalized polymers to form robust composites exhibiting tunable viscoelastic and tensile properties. Surface-exposed cysteines on the spore coat react with BCA motifs, forming dynamic thia-Michael networks with Young’s moduli of >100 MPa. Systematic variation of BCA reactivity and comonomer-dependent polymer dynamics enabled control over stiffness, stress-relaxation behavior, microscale morphology, and covalent biocontainment. Incorporation of engineered spores confers catalytic function that can be regenerated following solvent-triggered disassembly. This work establishes a modular platform for constructing biocomposites that are both mechanically and genetically programmable, bridging the synthetic and biological domains through molecularly defined interfaces.

## Graphical abstract

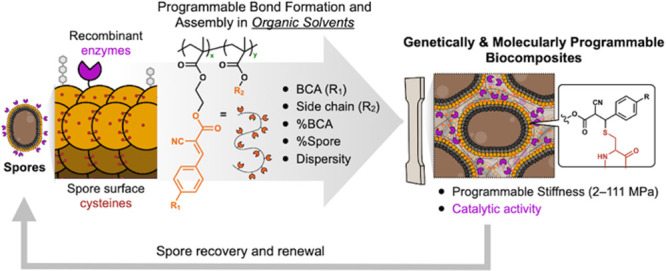

## Introduction

Biocomposite materials incorporating renewable biological sources are crucial for advancing sustainable technologies, particularly in resource-constrained settings.^1–4^ In addition, recent advances in synthetic biology present exciting opportunities to genetically engineer complex material functions such as catalysis, sensing, and therapy. Inspired by these possibilities, the field of engineered living materials has produced numerous examples of hybrid materials that integrate living cells for autonomous growth, regeneration, and genetically encoded functions.^5,6^ However, because living cells require mild, aqueous environments to remain viable, the macromolecular composition of these hybrid materials is typically limited to soft hydrogels.^7–10^ These hydrogels offer restricted mechanical tunability and narrow solvent compatibility, thereby constraining the broader design space that could be accessed with synthetic polymers.

A central challenge in engineering biocomposite materials is the incompatibility of biological entities with processing conditions for high-performance polymers, which typically involve heat or organic solvents. Certain biological particles, however, are naturally adapted to withstand harsh environments. *Bacillus subtilis* spores are metabolically dormant and partially dehydrated microparticles encased in a highly cross-linked protein shell.^11^ They can survive in conditions relevant to material synthesis and application, including organic solvents, elevated temperatures, and irradiation.^12–14^ Furthermore, providing nutrients to dormant spores initiates their germination and subsequent cell division, allowing for the full regeneration of new spores and engineered functions.^15^ Their durability, renewability, and genetic programmability to yield functional yet dormant particles make them uniquely well-suited as a building block for biocomposite materials.^16–18^

The physical properties and performance of composite materials depend on the interfacial compatibility between components.^2,19–21^ Establishing covalent connectivity between biological particles and polymeric matrices could be a compelling route to integrate the two, especially when such linkages are chemically tunable, reversible, and stimuli-responsive. In this regard, dynamic covalent bonds, with structure-dependent equilibrium constants (*K*_eq_) and dissociation rates (*k*_d_), offer precise control at the interface to modulate mechanical properties.^22–24^ Bond reversibility would also help recover both spores and polymers, enabling polymer recycling and the renewal of functional spores. We previously demonstrated self-assembly using dynamic covalent boronate ester bonds between phenylboronic acid-functionalized polymers and spore surface glycans, yielding hydrogels with reversible biocontainment and tunable stiffness.^25^ We envisioned that the organic solvent tolerance of *B. subtilis* spores could broaden the design space for polymeric matrices, enabling the fabrication of mechanically tunable, rigid, and functional biocomposite materials. While a few prior studies have embedded spores into polymers, the resulting materials exhibited limited mechanical tunability, likely due to insufficient interfacial connectivity between the biological and synthetic components.^17,18^ By introducing well-defined dynamic covalent linkages, we sought to strengthen polymer–spore interactions and achieve molecular-level control over biocomposite material properties.

In this study, we report the dynamic covalent assembly of *B. subtilis* spores with benzalcyanoacetate (BCA)-functionalized synthetic polymers in organic solvents. We identified abundant cysteines on the spore coat as reactive free thiols that undergo thia-Michael (tM) addition with BCA motifs. Macroscopic assemblies of spores and BCA-bearing polymers yielded gels with programmable stiffness and stress-relaxation behavior. Materials were processed into dry biocomposites and molecularly tuned to achieve mechanical properties ranging from 2 MPa to over 100 MPa in Young’s modulus. Entropy-driven disassembly enabled the recovery of polymers as well as spores for the renewal of fully catalytic spores. The catalytic activity of materials with engineered spores showcased the potential for this approach to yield a series of genetically programmable, renewable, and high-performance biocomposites.

## Results and Discussion

### Thia-Michael Bond Formation with Available Cysteines on *B. subtilis* Spores in DMSO

BCAs are highly electrophilic tM acceptors that form reversible BCA–thiol bonds (Figure 1a).^26^ This reaction exists in dynamic equilibrium because the ester and nitrile electron-withdrawing groups (EWGs) acidify the α-proton on the resultant adduct, promoting dissociation. Meanwhile, the β-aryl groups extend conjugation to stabilize the BCA, minimizing its energy difference with the adduct.^27–29^ *Para*-substituents (R) on the aryl ring further modulate adduct stability, providing structural control over *K*_eq_.^30,31^ Prior studies have shown that electronic tuning of BCA enables control over cross-link density and exchange kinetics in fully synthetic systems, thereby programming properties linked to material stiffness, stress relaxation, and glass transition temperature (*T*_g_).^32–34^ Using model reactions with BCA–R and 1-octanethiol, we confirmed that BCA–thiol reactivity is R-dependent and observed bond dissociation and exchange upon dilution and addition of competing thiols or BCAs (Figures S2.1–S2.3 and S4.1–S4.3). While full dissociation was not observed, the dynamic covalent character of these bonds could allow us to tune the mechanical properties of the biocomposite and help recover a significant portion of the polymer and spore for further analysis.

**Figure 1:**
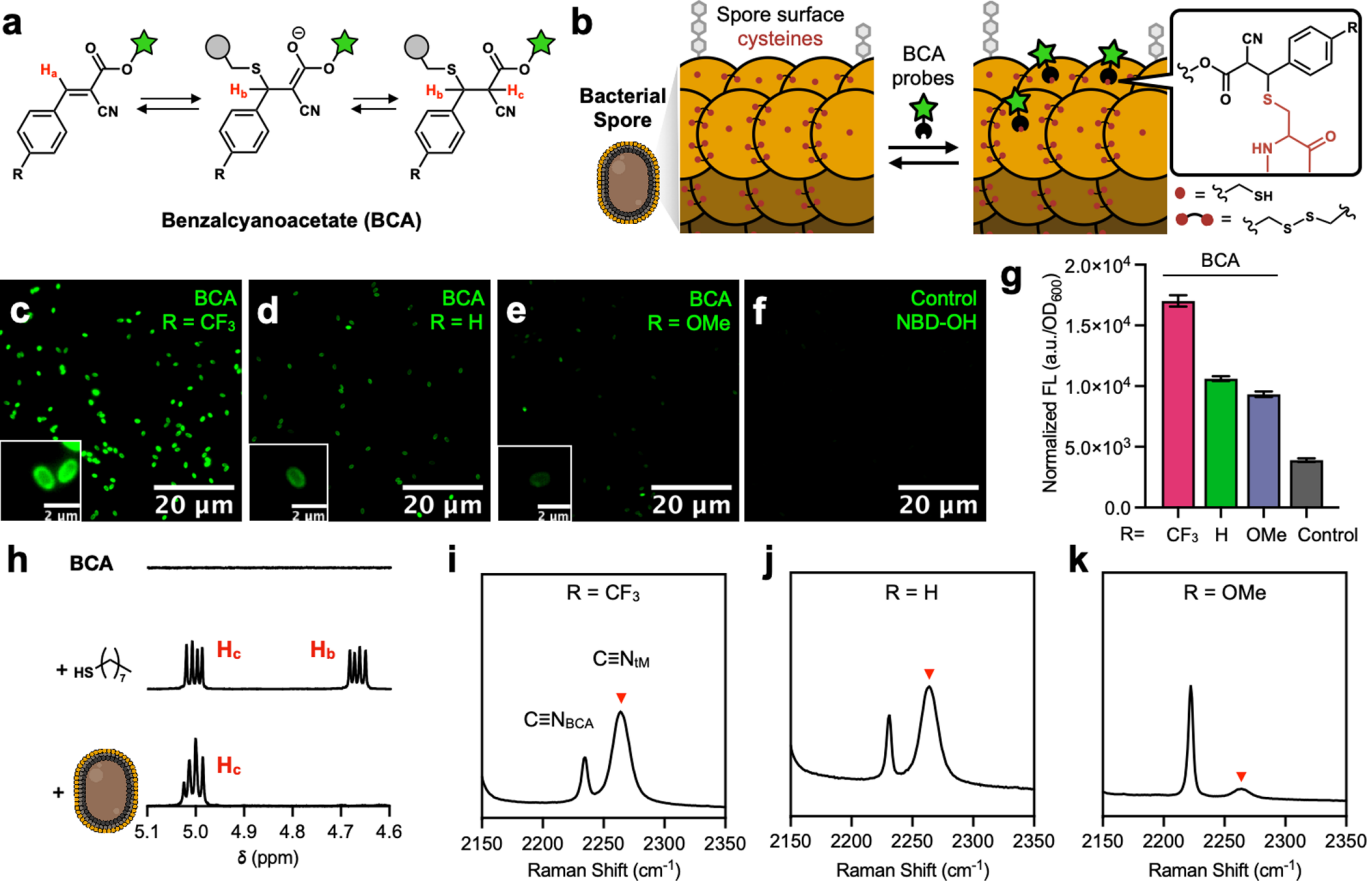
Thia-Michael (tM) bond formation with available cysteines on *B. subtilis* spores in DMSO**.** (a) Dynamic covalent tM addition to BCA. (b) Covalent attachment of fluorescently tagged BCA probes onto available cysteines on the spore surface in DMSO. (c–f) Representative fluorescence micrograph of spores incubated with various BCA probes compared to a control without BCA. (g) Normalized fluorescence emission of labeled spore suspensions. Error bars represent standard error of the mean (*n* = 3). (h) ^1^H NMR spectrum of BCA–CF_3_, with 1-octanethiol, and with spores. (i–k) Raman spectrum of spores incubated with BCA molecules bearing different aryl substituents. Red arrow = adduct.

*B. subtilis* spores exhibit exceptional stress tolerance, in part due to their outermost proteinaceous layer, the spore crust.^12^ The crust comprises several proteins – CotV, CotW, CotX, CotY, and CotZ – with CotY and CotZ being most abundant and notable for their high cysteine content, containing approximately 15 and 10 per protein, respectively.^35,36^ While some cysteines likely form disulfides as part of the native protein structure, we hypothesized that a fraction remains as free thiols, offering chemically addressable handles for covalent tM bond formation (Figure 1b).^37^ To assess the accessibility of surface-exposed thiols, we incubated spores overnight with sulfo-Cyanine5 maleimide, a commercially available thiol-selective fluorescent probe. Significant fluorescence was localized on the spore surface, compared to a sulfoCyanine-5 amine control, indicating the presence of reactive thiol groups (Figure S5.1). Fluorescence intensity increased with reducing agent tris­(2-carboxyethyl)­phosphine (TCEP), consistent with disulfide bond reduction and an increase in free thiol availability. Conversely, pretreatment with nonfluorescent maleimide substantially diminished labeling, validating that the fluorescence signal arose primarily from covalent thiol–maleimide conjugation. Together, these results support the presence of accessible surface cysteines on *B. subtilis* spores that are available for tM bond formation.

With confirmation of spore surface-accessible cysteine thiols, we next sought to test BCA addition for dynamic covalent tM addition in organic solvent. A series of fluorescent nitrobenzoxadiazole (NBD)–BCA probes were synthesized in three steps: (1) electrophilic aromatic substitution of NBD chloride with ethanolamine to install a terminal hydroxyl group, (2) Steglich esterification with cyanoacetic acid, and (3) Knoevenagel condensation with various benzaldehydes derivatives to install R-groups (Figures 1a, S2.4–S2.10). Spores incubated in DMSO at ambient temperature with these probes displayed fluorescence patterns that correlated with the electron-withdrawing strength of the R-group (CF_3_ > H > OMe), consistent with increased electrophilicity promoting thia-Michael bond formation (Figures 1b–f, S5.2–S5.3). In contrast, a control probe lacking the BCA motif (NBD–OH) showed negligible spore labeling. Bulk fluorescence measurements, normalized to spore concentrations (OD_600_), further established the R-dependent labeling efficiency of the probes, reinforcing the role of BCA electronics in governing covalent bond formation on the spore surface (Figure 1g).

TM adduct formation on the spore surface was further supported by nuclear magnetic resonance (NMR) spectroscopy. When BCA–CF_3_ was incubated with lyophilized spores in DMSO-*d*_6_, a characteristic overlapping doublet corresponding to the adduct’s α-proton (H_c_) appeared at 5.0 ppm (Figure 1h). The peak intensity decreased upon D_2_O addition, consistent with deuterium exchange at the enolizable α-position (Figure S5.4). Notably, the neighboring benzylic proton (H_b_) appeared upfield at 4.2 ppm as evidenced by a cross-peak observed in ^1^H–^1^H correlation spectroscopy (COSY) (Figure S5.4). This peak also showed the expected asymmetric decrease in doublet intensity upon D_2_O addition. Maleimide–spore adducts likewise exhibited altered chemical shifts but retained clear splitting patterns and relative intensities matching those of model reactions with 1-octanethiol (Figure S5.5). These observations suggest that the shielding environments of key methine protons are perturbed by the local spore surface microstructure, which may involve dielectric heterogeneity, restricted solvation, and surface charge density.^38^ Additional support for covalent conjugation came from the R-dependent appearance of diagnostic NMR signals: well-resolved adduct peaks were observed with highly electrophilic BCAs (R = CF_3_), while less reactive analogs (R = H, OMe) gave weak signals (Figure S5.6). Across all BCA variants, the relative shifts of the α-proton peaks aligned closely with those from model thiol adducts, reinforcing the conclusion that BCA–spore adducts formed through bonding with surface-exposed cysteine residues.

To further probe tM adduct formation on the spore surface, Raman spectroscopy was performed on DMSO mixtures of spores and BCA derivatives. Nitriles exhibit distinct Raman-active stretching vibrations due to their strong dipole and low background in biological samples. In BCA compounds, thiol addition disrupts conjugation between the alkene and nitrile groups, resulting in a blue shift of the nitrile stretching frequency from ∼2229 cm^–1^ (unreacted BCA) to 2264 cm^–1^ (tM adduct).^32^ We observed this diagnostic shift in our BCA–spore mixtures, along with R-dependent peak ratios between free BCA and the tM adduct (Figures 1i–k and S15). This trend verifies that electron-withdrawing substituents enhance BCA electrophilicity and promote adduct formation. The structure–reactivity relationship between BCA derivatives and spores and was consistent across fluorescence, ^1^H NMR, and Raman spectroscopy, supporting the formation of tM bonds. Notably, the organic solvent tolerance of *B. subtilis* spores enabled the use of three different spectroscopic methods, allowing direct, multimodal observation of covalent bond formation at the biological interface.

### Programmable Dynamic Covalent Assembly between *B. subtilis* Spores and BCA-Appended Copolymers in DMSO

We hypothesized that covalent bond formation in DMSO could facilitate the assembly of polymers with bacterial spores, yielding organogels. To incorporate the key functionality, we synthesized BCA methacrylate (BCAMA) monomers on decagram scales in two steps from 2-hydroxyethyl methacrylate (Figures S2.11–S2.19). Methyl methacrylate (MMA, R_2_ = CH_3_) and ethylene glycol methyl ether methacrylate (EGMEMA, R_2_ = CH_2_CH_2_OCH_3_) were selected as comonomers due to their high DMSO solubility and structural diversity to build a polymer library with varying polarity and backbone rigidity (Figure 2a). Polymers used in this study are summarized in Figure 2b (Figures S2.20–S2.33 and S3).

**Figure 2:**
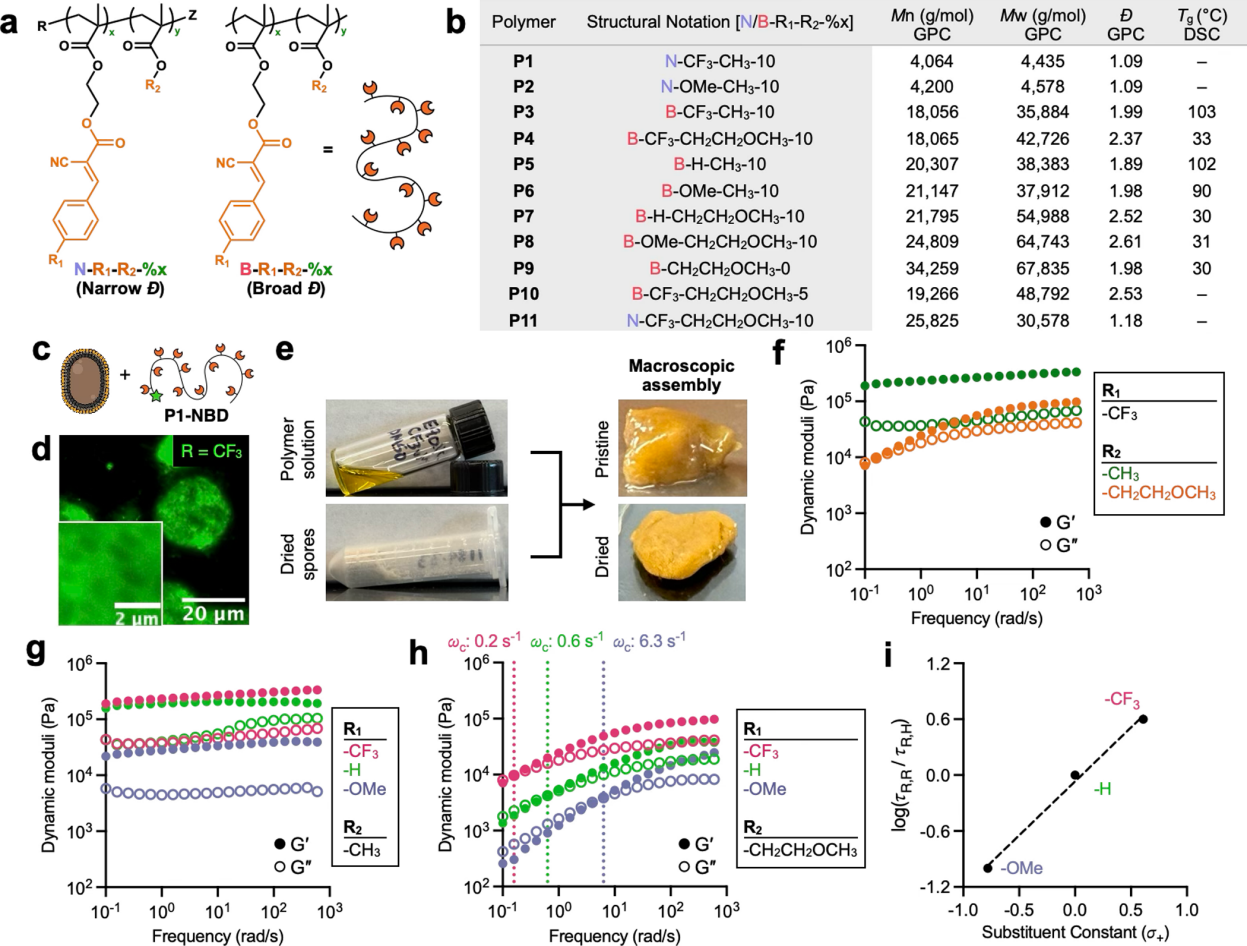
Programmable dynamic covalent self-assembly between *B. subtilis* spores and BCA-appended copolymers in DMSO. (a) Schematic illustration of BCA-appended copolymers synthesized by free radical or RAFT polymerization with variable identities and compositions of aryl substituents and comonomers. (b) Table of structurally diverse polymers characterized for their *M*_n_, *Đ*, and *T*_g_. (c) Incubation of a fluorescently tagged BCA copolymers with spores in DMSO. (d) Representative fluorescence image of polymer–spore assemblies showing localized fluorescence on the spore surface. (e) Macroscopic assembly of lyophilized spores with 30% w/v polymer solutions (4:1 mass ratio of polymer to spore). (f) Frequency sweep of materials assembled from copolymers with a constant R_1_ = CF_3_ and varied R_2_ at 25 °C, highlighting comonomer-dependent dynamic moduli. (g) Stiffness profiles for materials with fixed R_2_ = CH_3_ and varied R_1_. (h) Viscoelastic behavior of materials with fixed R_2_ = CH_2_CH_2_OCH_3_ and varied R_1_, revealing a crossover frequency indicative of aryl substituent-dependent relaxation dynamics. (i) Linear free energy relationship between the log of relaxation time and Hammett σ^+^ constants of aryl substituents R_1_ revealed a strong positive correlation (slope ρ = +1.16 and *R* ^2^ = 0.99).

Polymer–spore interaction was first examined using fluorescence microscopy. We synthesized polymers with 10 mol % BCAMA and 90 mol % methyl methacrylate (MMA) composition using reversible addition–fragmentation chain transfer (RAFT) polymerization and conjugated the carboxylic acid terminal group with NBD to afford fluorescent P1–NBD (R_1_ = CF_3_) and P2–NBD (R_1_ = OMe) (Figures 2c and S2.20–S2.23). In DMSO under dilute conditions (1.2 μM), P1-NBD assembled with spores into micron-scale assemblies displaying localized fluorescence on the spore surface (Figure 2d). P1-NBD yielded higher fluorescence intensity and larger aggregate size compared to P2-NBD, consistent with the relative reactivity of BCA monomers (Figure S6).

With direct observation of polymer–spore interactions, we next explored macroscopic assembly and comonomer effects. Free radical polymerization of BCAMA (R_1_ = CF_3_) with comonomers at a 0.1:0.9 feed ratio yielded copolymers P3 (R_2_ = CH_3_) and P4 (R_2_ = CH_2_CH_2_OCH_3_) using MMA and EGMEMA, respectively (Figures S2.24–S2.25). These polymers exhibited number-average molecular weights (*M*_n_) of ∼20 kDa and dispersities (*Đ*) ranging from 2.0 to 2.4 and readily dissolved in DMSO at 30% w/v. Overnight incubation and mixing of the polymer solutions with lyophilized spores yielded self-standing organogels (Figure 2e). However, they continuously excreted DMSO from their surfaces, creating a slippery film that interfered with analyses of the materials’ internal structures. We thus implemented a solvent equilibration protocol by allowing samples to rest uncovered on the benchtop for 2 days and wiping off any excess solvent prior to measurements.

Rheological measurements revealed that materials made with P4 (R_2_ = CH_2_CH_2_OCH_3_) were less stiff than those made with P3 (R_2_ = CH_3_) (Figure 2f). This difference aligns with a softening mechanism driven by polymer–solvent interactions and correlates with the decreasing Hansen solubility parameter distance (*R*_a_) between DMSO and each polymer’s side chain structure: *R*_a,P4_ = 3.4 < *R*_a,P3_ = 6.1 (Section S7).^39^ Lower *R*_a_ values reflect stronger compatibility with DMSO, promoting solvent retention and plasticization. Consistent with this interpretation, P3-based gels expelled DMSO more rapidly during storage (Figure S8), resulting in network densification and increased stiffness. Differential scanning calorimetry (DSC) further showed *T*_g_ values of 103 °C for P3 and 33 °C for P4, highlighting the rubbery character of P4 compared to the glassy nature of P3 in the dry state (Figure 2b). Thus, the combined effects of greater DMSO plasticization and lower intrinsic backbone rigidity account for the reduced stiffness of P4 materials compared to P3 materials.

The influence of BCA reactivity was first observed with materials made using MMA-based copolymers (R_2_ = CH_3_) – including P3 (R_1_ = CF_3_), P5 (R_1_ = H), and P6 (R_1_ = OMe) (Figure 2g). These gels exhibited high moduli with relative stiffness that mirror BCA electrophilicity (CF_3_ > H > OMe), indicating that persistent cross-linking was achieved in the rigid MMA matrix. This result demonstrates the influence of BCA in dictating organogel mechanics, with contributions from both cross-link density and polymer–solvent interactions.

Programmable viscoelastic behavior was observed upon assembly of *B. subtilis* spores with EGMEMA-based copolymers (R_2_ = CH_2_CH_2_OCH_3_), P4 (R_1_ = CF_3_), P7 (R_1_ = H), and P8 (R_1_ = OMe) (Figure 2h). The resulting gels exhibited tunable storage moduli (*G*′) and crossover frequencies (ω_c_) that were consistent with the relative BCA reactivity and cross-link exchange kinetics, reflecting the relative *K*_eq_ and *k*_d_ of the BCA derivatives (Figure S9). Notably, relaxation time (τ_R_ = 1/ω_c_) showed a strong positive linear correlation with the Hammett constants (σ^+^) of the BCA aryl substituents (*R* ^2^ = 0.99), supporting the conclusion that network dynamics are governed by electronic effects (Figure 2i).^40–42^ From this correlation, the sensitivity of network dynamics to R_1_ (ρ = +1.16), derived from the Hammett-type relationship log­(τ_R,R_/τ_R,H_) = ρ · σ^+^, was found to qualitatively match the trend observed in small-molecule dissociation kinetics (ρ = +1.42, Figure S4.2). This attenuation in ρ could suggest a partial decoupling of bond exchange dynamics from stress relaxation due to restricted chain mobility.^43^ Together, these results demonstrate that tuning in the electronic properties of the BCA handle and the structure of comonomer side chains enables dynamic covalent chemistry to operate coherently across molecular, mesoscopic, and macroscopic length scales, thereby controlling the stiffness and stress-relaxation behavior of polymer–spore organogels.

### BCA Reactivity Dictates Mechanical Reinforcement and Biocontainment of *B. subtilis* Spores in Dry Composites

Hybrid materials incorporating cells are typically processed as soft hydrogels to mimic tissue-like environments, whereas conventional thermoplastics and thermosets exhibit mechanical robustness through strong interchain interactions and covalent cross-linking. We hypothesized that the solvent stability of *B. subtilis* spores could expand the design space of hybrid materials to afford dry biocomposites with enhanced tensile properties driven by dense, persistent polymer–spore interactions. Spores were assembled with 30% w/v polymer solution in DMSO, cast into dog bone molds, subjected to a solvent exchange by ethanol immersion (Figure S10.1), and dried on polytetrafluoroethylene sheets. Materials prepared with P3 (R_2_ = CH_3_) dried rapidly but became brittle, whereas P4 (R_2_ = CH_2_CH_2_OCH_3_) yielded robust, flexible composites that could be reproducibly processed in ethanol (Supplementary Video). This difference likely reflects variations in polymer polarity and backbone rigidity (*T*_g_). Notably, P4 materials softened in ethanol yet retained structural integrity, enabling clean release from the mold. We therefore selected the EGMEMA-based, low-*T*_g_ architecture of P4 for tensile experiments (Figure 3a).

**Figure 3:**
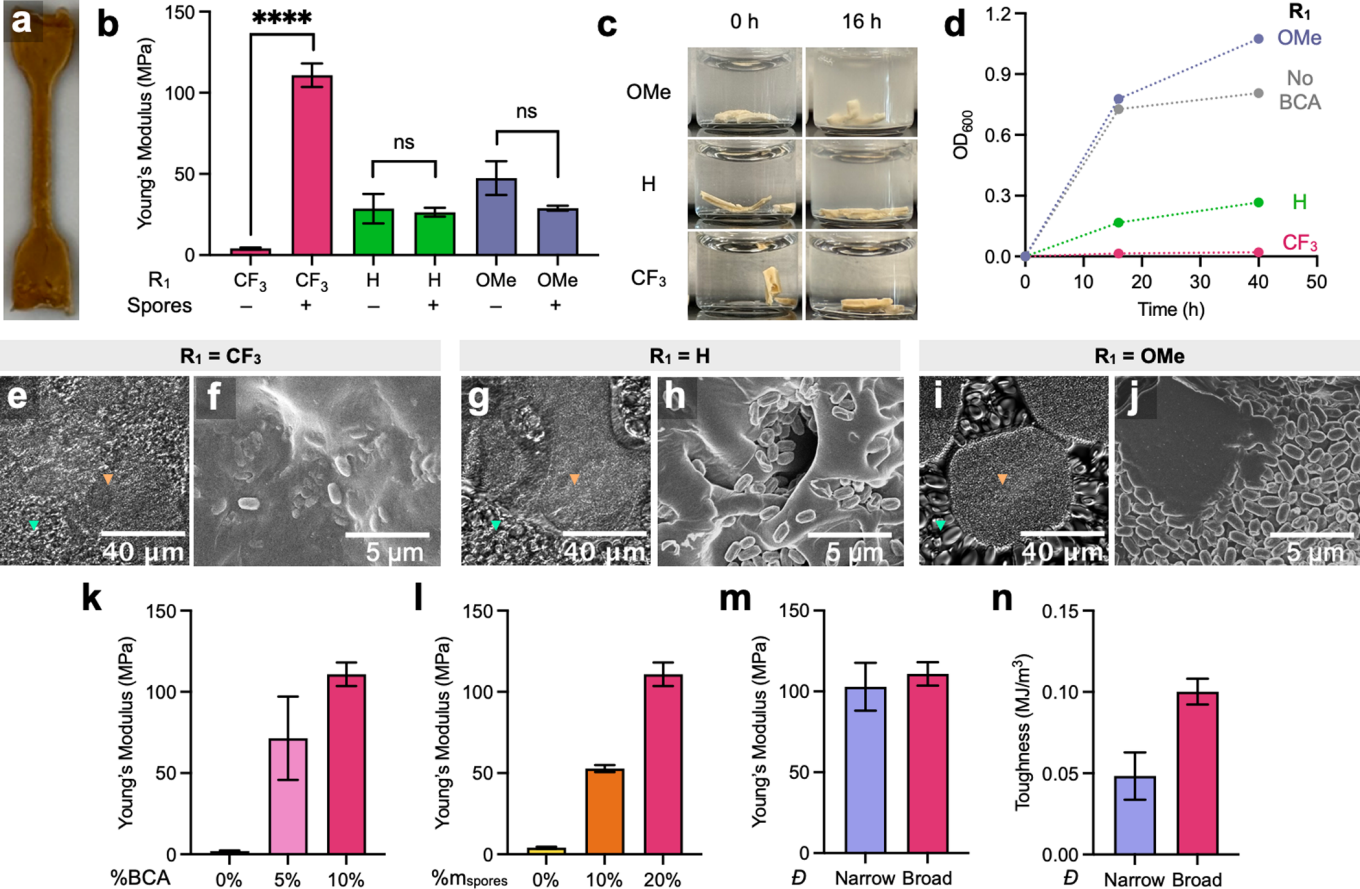
BCA reactivity dictates mechanical reinforcement and biocontainment of *B. subtilis* spores in dry composites (R_2_ = CH_2_CH_2_OCH_3_). (a) Dry biocomposite materials processed into dog bone shapes (25 mm × 5 mm × 1 mm, 4:1 mass ratio of polymer to spore). (b) Young’s moduli of EGMEMA-based materials with (+) and without (−) spores using polymers with different aryl substituents. (c) Visually tracked spore leakage from materials with different aryl substituents. (d) Solution turbidity monitored by optical density measurements (OD_600_) and baseline subtracted by OD_600_ = 0.085 of PBS. (e–j) Optical and scanning electron micrographs of polymer–spore mixtures. Orange arrow = spore-rich phase. Teal arrow = polymer-rich phase. SEM scale bar = 5 μm. (k) Young’s moduli of materials with different %BCA–CF_3_ composition. (l) Stiffness dependence on %w/w spores within materials made with P4 (R_1_ = CF_3_). (m) Young’s moduli of materials made using broad (*Đ* = 2.4, *M*_n_ = 18 kDa) and narrow (*Đ* = 1.2, *M*_n_ = 26 kDa) dispersity polymers with R_1_ = CF_3_. (n) Effect of molecular weight and dispersity differences on material toughness. All error bars represent standard error of the mean (*n* = 3). *P* values were determined by unpaired two-tail *t*-tests. *P < 0.05, **P < 10^–2^, *** *P* < 10^–3^, and**** *P* < 10^–4^.

Dry composites were prepared with (+) and without (−) *B. subtilis* spores using P4 (R_1_ = CF_3_), P7 (R_1_ = H), and P8 (R_1_ = OMe) to evaluate the hypothesis that covalent spore–polymer interactions enhance mechanical reinforcement in accordance with BCA reactivity (Figure 3b). While P4 exhibited an increase in Young’s modulus of approximately 100 MPa upon spore addition, P7 and P8 showed negligible changes. These results suggested that high BCA electrophilicity was required to achieve mechanical reinforcement by covalent spore integration. This cross-link-dependent tensile behavior of P4 materials further implied their parallel capacity for covalent biocontainment. To evaluate this, material fragments were incubated in phosphate-buffered saline (PBS, pH = 7.2) at 37 °C and 250 rpm overnight (Figure 3c). P4 composites retained embedded spores, while P7 and P8 samples exhibited measurable turbidity in the supernatant, indicating spore leakage (Figure 3d). Spore release inversely correlated with BCA electrophilicity, confirming that weaker polymer–spore bonding compromised containment. Although P7 (R_1_ = H) did not significantly stiffen upon spore addition, it retained spores more effectively than P8 (R_1_ = OMe), which showed leakage levels comparable to a control polymer, P9, lacking BCA (Figure S10.2). These findings establish that BCA reactivity dictates both mechanical reinforcement and covalent biocontainment in dry biocomposites.

Optical and scanning electron microscopy (SEM) were used to visualize polymer–spore interactions before and after the solvent exchange and drying process. In DMSO, optical microscopy revealed distinct spore-rich (yellow arrow) and polymer-rich (white arrow) domains (Figures 3e,g,i and S10.3). The extent of interfacial blending increased with the electrophilicity of the BCA motif (R_1_ = OMe < H < CF_3_), reflecting differences in equilibrium bond formation. Polymers bearing the highly reactive CF_3_ group exhibited near-continuous interfaces with spores (Figure 3e), while OMe-substituted polymers displayed poor integration and phase separations (Figure 3i). These trends mirrored the *K*_eq_-dependent cross-linking efficacy observed in small-molecule tM model systems in DMSO-*d*_6_ (Figure S4.2). SEM images of dry material fragments further supported this correlation, showing extents of spore embedding that tracked with BCA reactivity (Figures 3f,h,j and S10.4) CF_3_-substituted polymers exhibited uniform incorporation (Figure 3f), while OMe analogs and non-functionalized controls showed limited interfacial bonding and retention (Figure 3j). These results reinforce that strong polymer–spore interactions arise from BCA-mediated covalent cross-linking and are crucial for structural coherence in the dry state.

Tensile properties of the CF_3_-based biocomposites were tunable through modulation of both polymer and spore composition. Decreasing the BCA content from 10% (P4) to 5% (P10) and 0% (P9) led to a stepwise decrease in Young’s modulus from 111 to 71 and 2 MPa, respectively, confirming that tM cross-links significantly stiffen the material (Figure 3k). Likewise, reducing the spore content from 20 wt % to 10 and 0 wt % with P4 yielded moduli of 111, 53, and 4 MPa, respectively, highlighting the reinforcing role of spores (Figure 3l). To examine the effect of polymer molecular weight dispersity, we synthesized a low-dispersity RAFT copolymer (P11, *Đ* = 1.2) with a comparable *M*_n_ to P4. While the modulus remained comparable, P11 exhibited reduced toughness, likely due to diminished ductility associated with the absence of high molecular weight chains in its narrower molecular weight distribution (Figure 3m,n).^44^ Collectively, these results demonstrate that the mechanical performance of biocomposites can be precisely tailored through reactive handle density, spore loading, and polymer dispersity.

### Entropy-Driven Material Disassembly in Organic Solvent for the Recovery and Growth of Bacterial Spores

On-demand dissociation of polymer–spore linkage offers a strategy to control material fate at end-of-life, potentially supporting both polymer recycling and spore renewal depending on the extent of disassembly. Disassembly can be triggered by shifting the dynamic covalent equilibrium through increased polymer entropy or the introduction of competitive thiols. We monitored the dissociation of a model tM adduct formed between BCA and 1-octanethiol using ^1^H NMR spectroscopy to investigate these possibilities (Figure S4.3). Among the tested conditions, thiol exchange most effectively promoted bond reversal. However, dilution offered a particularly simple and mild method; by using organic solvent as the only variable in both assembly and disassembly, we also evaluated the effect of prolonged solvent exposure on *B. subtilis* spore stability and viability.

We selected DMSO and acetone as disassembly solvents because polymers dissolved well in both. Fragments of P4-based materials were submerged in DMSO or acetone to initiate entropy-driven disassembly, and the resulting crude mixtures were centrifuged to isolate and analyze the recovered components (Figure 4a). ^1^H NMR analysis showed that the polymer could be recovered in pure form from both solvents within 30 min (Figure 4b). To evaluate spore release efficiency, we examined aqueous suspensions of the recovered pellets using optical microscopy (Figure 4c,d). After 30 min, spores recovered from DMSO appeared well dispersed, whereas those from acetone-treated samples showed visible aggregation (Figure S11.1). After 8 h, complete disassembly was achieved in both solvents (Figure 4e). To investigate the effect of solvent polarity on dynamic covalent reactivity, we performed a model tM reaction in acetone-*d*_6_, monitored via ^1^H NMR spectroscopy (Figure S4.2). The extent of adduct formation varied with BCA R-group identity, and full conversion required 1 day in acetone compared to about 1 h in DMSO. This difference likely reflects DMSO’s higher dielectric constant (ε = 47), which better stabilizes polar or charged transition states and intermediates than acetone (ε = 21), thereby accelerating both the forward and reverse pathways.^27,45^ Nevertheless, acetone remains a practical alternative due to its volatility and ease of removal during spore and polymer recovery.

**Figure 4:**
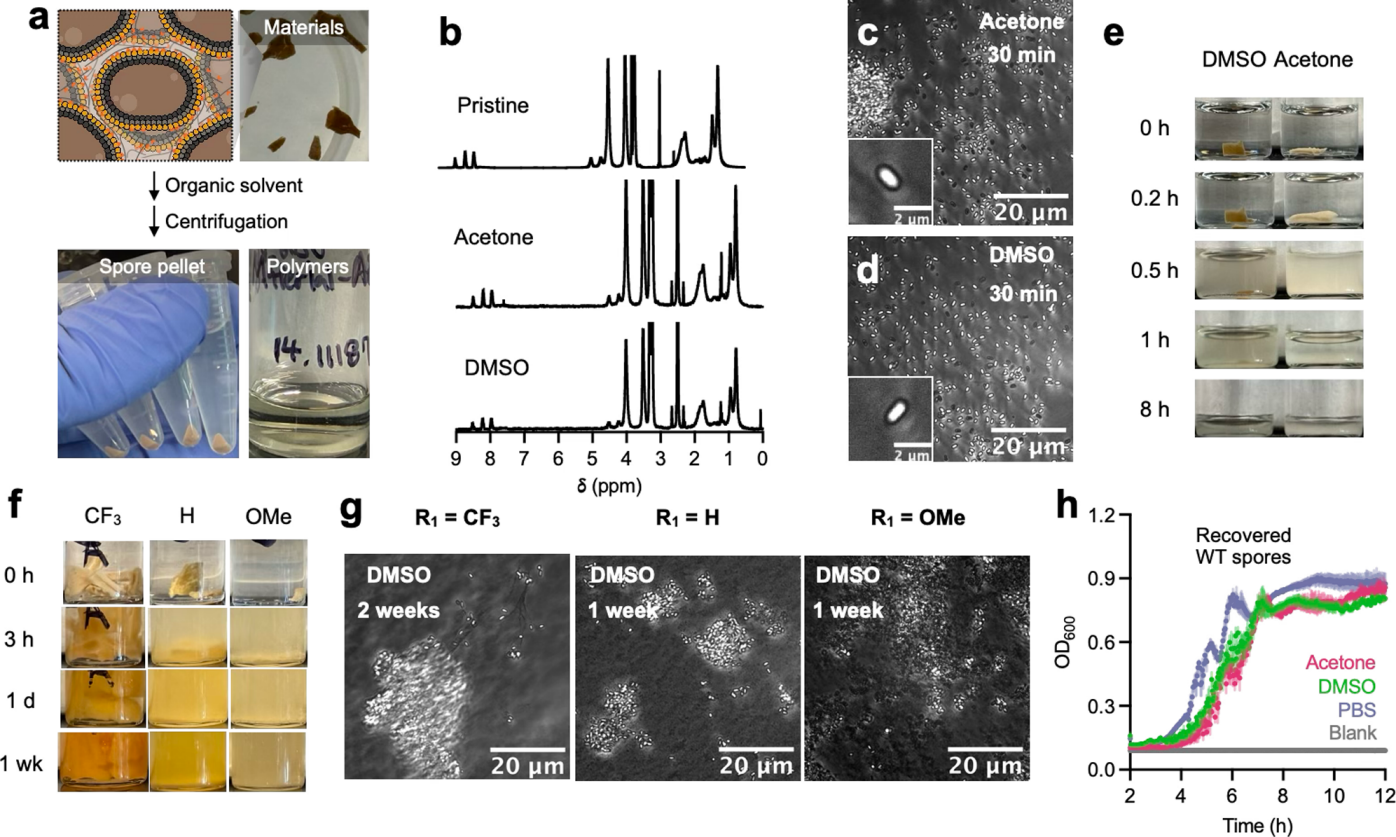
Entropy-driven material disassembly in organic solvent for the recovery and growth of bacterial spores. (a) Polymer–spore disassembly in organic solvents. (b) ^1^H NMR spectra of the pristine P4 and P4 isolated from acetone and DMSO after 30 min of material incubation. (c, d) Optical micrographs of crude polymer and spore mixtures in acetone and DMSO after 30 min. (e) P4-based material disassembly in DMSO and acetone. (f) Lyophilized materials with different R_1_ groups showing different relative rates of disassembly in DMSO at 37 °C and 250 rpm. (g) Optical micrographs showing polymers, spores, and polymer–spore aggregates in DMSO after material incubation in DMSO with different R_1_ groups. (h) Growth curve of spores recovered from P4 material disassembly in acetone and DMSO compared to that of untreated spores.

When disassembly is performed on materials that have undergone additional thermal incubation and subsequent lyophilization to remove any residual solvents or moisture, the progression is markedly slow (Figure 4f). We speculate that the additional thermal incubation may have promoted additional cross-linking, while lyophilization removed moisture that had previously facilitated solvent penetration and chain mobility. The extent of disassembly, based on the turbidity of the mixture, mirrored the R_1_-group-dependent reactivity trends. Micrographs of the supernatant showed this dependence: CF_3_-substituted polymers resisted full disassembly for 2 weeks, while H- and OMe-substituted variants underwent near-complete or complete disassembly within a week (Figure 4g). CF_3_-based materials were subjected to further dilution, mechanical agitation, and heating. Spore release was effective at 0.2 mM, 1000 rpm, and 80 °C, but resulted in polymer degradation (Figure S11.2). NMR spectrum revealed polymers that are likely still bound to cysteine residues (Figures S11.3–11.4). Comparison of CF_3_-, H-, and OMe-based polymers confirmed that the extent of polymer–thiol dissociation depends on BCA reactivity, with the recovered P8 (R_1_ = OMe) being identical to the pristine P8 (Figure S11.5–11.6).

To assess whether prolonged exposure to organic solvents compromised spore integrity, we tested the viability of spores through recovered from 8-h disassembly in DMSO or acetone. Recovered spores were dried and resuspended in PBS to a standardized optical density (OD_600_ = 0.36 ± 0.03). When 1 μL aliquots were inoculated into 100 μL of Lysogeny Broth (LB), all samples germinated and reached saturation overnight (Figure 4h). Spore viability was also tested directly in intact materials. P4 composites were submerged in LB and incubated at 37 °C with shaking (250 rpm). Even without disassembly, bacterial growth reached saturation in 1 day, indicating that surface-accessible spores remained viable (Figure S12). No significant increases in turbidity were observed in nutrient- or oxygen-depleted conditions, indicating that growth originated primarily from the material surface rather than from spore leakage. These findings show that germination can occur without network degradation, potentially enabling self-regenerating or signal-responsive behavior in future designs of living materials. Lastly, colony-forming unit (CFU) assays revealed that ∼51% of spores remained viable following material assembly conditions, consistent with the robust germination observed both before and after material disassembly (Figure S16).

### Catalytic Activity of Materials Assembled in Organic Solvent

Incorporating engineered spores displaying recombinant enzymes yielded catalytic biocomposite materials capable of facilitating specific chemical reactions (Figure 5a).^16^ We first evaluated the effect of organic solvents on the activity of the spore-displayed APEX2 by monitoring the production of fluorescent Resorufin from Amplex Red with H_2_O_2_ as a cofactor (Figure 5b). Engineered spores with loading densities of >10^6^ enzymes per spore were incubated in various solvents overnight prior to standardized activity assays (Figure S13.1).^16^ Catalytic activity was strongly solvent-dependent: DMSO caused near-complete loss of activity, while acetone, ethanol, and methanol preserved partial function (Figure 5b). Optical microscopy revealed corresponding morphological changes. DMSO-treated spores underwent structural collapse, acetone-exposed spores retained their native appearance, and ethanol/methanol induced visible swelling (Figure 5c–f). These changes were specific to APEX2 spores with high recombinant enzyme density and were not observed in wild-type controls (Figure 4d–g). Wild-type spores appeared predominantly as bright-phase particles due to their partially dehydrated core, which present a refractive index contrast relative to the solvent medium. On the other hand, the dark-phase APEX2 spores likely have altered external protein layers may increase organic solvent permeability.

**Figure 5:**
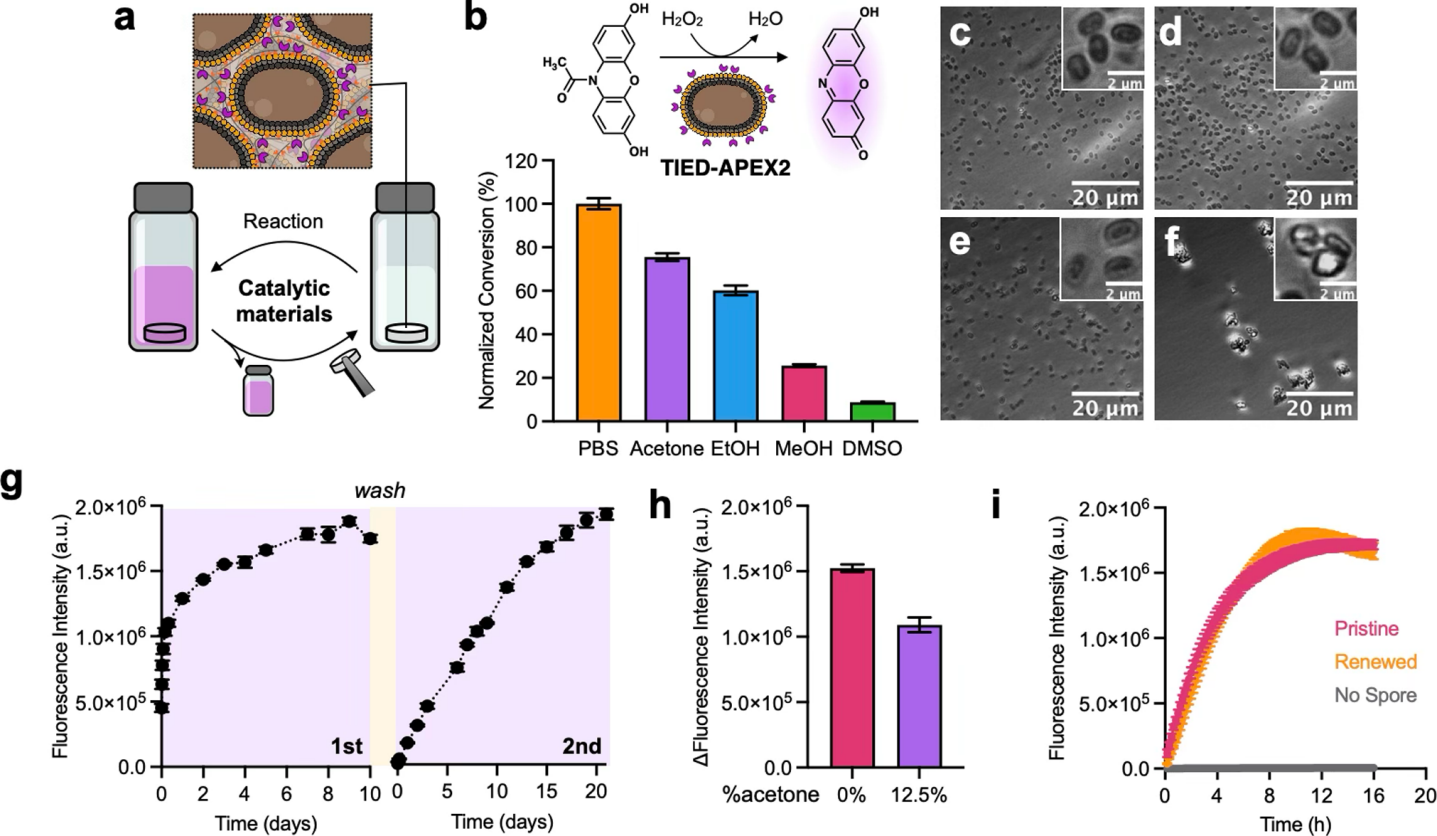
Catalytic activity of materials assembled in organic solvent. (a) Schematic illustration of reusable catalytic materials enabled by the molecular assembly of polymers with engineered spores. (b) Schematic illustration of the APEX2-catalyzed conversion of Amplex Red to colorogenic and fluorogenic product, resorufin. Relative differences in Amplex Red conversion using APEX2-displaying spores incubated in various solvents. (c–f) Micrographs of APEX2-displaying spores that were incubated in (c) PBS (pH = 7.16), (d) acetone, (e) ethanol, and (f) DMSO, and then resuspended in PBS. (g) Reaction conversion tracked with bulk fluorescence emission over time in samples containing catalytic materials. Reuse of the material shown in a second reaction cycle. (h) Relative conversion of the reaction run with the catalytic material in PBS vs 12.5% acetone in PBS. (i) Recovered catalytic activity of APEX2-displaying spores after recovery, germination, growth, and resporulation (renewal). All error bars represent standard error of the mean (*n* = 3).

Viability studies further supported these observations. Overnight incubation of APEX2 spores in a combination of DMSO and ethanol reduced survival to ∼16% compared to ∼51% for wild-type spores, whereas acetone treatment increased survival of APEX2 spores 2.4-fold to ∼39% (Figure S16). Together, these results indicate that the viability and catalytic activity of APEX2 spores are highly sensitive to the solvent environment, with enzyme denaturation and structural compromise of spores likely explaining the loss of function. These findings underscore the importance of solvent compatibility in designing enzyme-functionalized biocomposite materials and guided our selection of acetone for catalytic biocomposite assembly.

Catalytic biocomposite materials were assembled in acetone with P4 and APEX2-displaying spores and assessed by immersing them in a reaction buffer containing Amplex Red and H_2_O_2_. The acetone-assembled material successfully catalyzed the reaction with full efficiency, in stark contrast to the DMSO-assembled counterpart that showed negligible activity (Figures 5g and S13.2–13.3). The material could be reused for a second cycle, but the reaction rate was slower, suggesting that extended incubation in acetone might partially impair the enzymes’ structure while still preserving some catalytic activity. Given that both acetone and ethanol caused minimal damage to APEX2 spores, we next explored the feasibility of biocatalysis in aqueous–organic mixtures. A long-standing challenge in biocatalysis is the poor compatibility between nonpolar, organic solvent-soluble substrates and enzymes that are typically unstable in organic solvents.^46^ We found that catalytic activity was generally decreased with an increasing organic solvent content but remained significant at 12.5% acetone (Figures 5h and S13.4).

While organic solvent exposure during both material assembly and catalysis can partially damage the catalytic output of a spore-displayed enzyme, germination of spores could offer a route to completely restore catalytic activity. To test this, we disassembled catalytic materials in acetone and recovered some of the embedded spores (Figure S14). Upon resuspension in LB medium, the spores successfully germinated, and the resulting bacterial cells were resporulated to yield a new population of fully catalytic spores (Figure 5i). This sustainable strategy restores the functional properties of biocomposite materials, uniquely possible due to the dormant, resilient, and genetically tractable nature of spore particles.

## Conclusions and Outlook

This work presents a modular framework for covalently integrating functional bacterial spores into mechanically robust synthetic polymers using dynamic covalent tM chemistry at the biology–material interface. By targeting surface-exposed cysteine residues on *B. subtilis* spores with BCA-functionalized polymers, we achieved tunable cross-linking in organic solvents to form organogels and dry biocomposites with emergent properties spanning molecular to macroscopic scales. Systematic variation of BCA electrophilicity and comonomer identity revealed clear structure–property relationships: highly reactive BCAs, when combined with polymers bearing polar and conformationally flexible side chains (e.g., EGMEMA), promoted enhanced viscoelastic stiffness, tensile reinforcement, and covalent biocontainment. The reversible polymer–spore conjugation enabled disassembly and recovery of both polymer and viable spores, laying the groundwork for recyclable and regenerative materials. Incorporation of enzyme-displaying spores endowed the composites with catalytic activity that can be recovered via germination and resporulation. Importantly, investigation of polymer–spore–solvent compatibility enabled the development of materials with stiffnesses exceeding 100 MPa while preserving microbial function and regenerative capacity.

Our findings demonstrate that molecular compatibilization of synthetic polymers with bacterial spores enables the development of mechanically and functionally programmable biocomposites. Central to this approach is the translation of interfacial reactivity across length scales, encoding macroscopic properties at the molecular level. This strategy is likely generalizable to other spore and extremophile fillers, which could afford new composites with alternative chemistries and functions. Altogether, this platform establishes foundational design principles for new biocomposites capable of circular lifecycles, environmental sensing, or catalytic deployment in both aqueous and organic environments – bridging a long-standing gap between synthetic polymer chemistry and microbial engineering at the macroscopic level.

## Supplementary Material





## Data Availability

The data generated in this study are archived at the Dryad Repository: 10.5061/dryad.573n5tbmt.

## Notes

Materials; instrumentations; experimental and synthetic methods; ^1^H, ^13^C, ^1^H–^1^H COSY, and ^1^H–^1^C HMQC NMR spectra; HRMS data; GPC measurements; fluorescence microscopy and spectroscopy results; Hansen solubility parameter calculations; rheological characterization; solvent evaporation; tensile testing; material preparation; SEM images; biocatalysis; spore germination and renewal; OD_600_ measurements; optical images of materials and reaction mixtures (PDF)
Video: Biocomposite material after solvent exchange (MOV)
