# Supplementary material for: Programmable Assembly of Mechanically Robust and Functional Polymer–Spore Biocomposites in Organic Solvent
Source: J Am Chem Soc. 2025 Oct 21;147(44):40859–68. doi: 10.1021/jacs.5c13976 (PMC12593326; doi:10.1021/jacs.5c13976)
Supplement: Supplementary file 1 [file ja5c13976_si_001.pdf]

Supporting Information

Programmable Assembly of Mechanically Robust and Functional Polymer–Spore Biocomposites in Organic Solvent

Masamu Kawada,[1] Ziyu Cui,[2] Justin Chen,[1] Reis Dorit,[1] Jaeho Cho,[1] and Seunghyun Sim[1,2,3,4] *

[1]*Department of Chemistry, University of California Irvine, Irvine, CA 92697, USA*

[2]*Department of Chemical and Biomolecular Engineering, University of California Irvine, Irvine, CA 92697, USA*

[3]*Department of Biomedical Engineering, University of California Irvine, Irvine, CA 92697, USA*

[4]*Center for Complex and Active Materials, University of California Irvine, Irvine, CA 92697, USA*

Table of Contents

S1. Materials, Instrumentations, and Methods **2**
S2. Synthesis and Characterization of Chemical Compounds **7**
S3. Gel Permeation Chromatography (GPC) Measurements **32**
S4. BCA Reactivity in Organic Solvents (DMSO-*d*$_6$ and Acetone-*d*$_6$) **34**
S5. Spore Labeling **37**
S6. Fluorescent Polymer–Spore Assemblies **43**
S7. Approximation of Polymer–DMSO Affinity **44**
S8. Solvent Expulsion and Evaporation from Material **45**
S9. Rheological Characterization and Analysis of Organogels **46**
S10. Preparation and Characterization of Biocomposites **47**
S11. Material Disassembly and Analysis **50**
S12. Spore Viability After Organic Solvent Exposure **56**
S13. Catalytic Materials Assembled in Organic Solvent **57**
S14. Recovery and Renewal of Catalytic Spores **61**
S15. Raman Spectroscopy Analysis **62**
S16. CFU Assay **63**
S17. References **64**

S1. Materials, Instrumentations, and Methods

S1.1. Materials: Reagents and solvents were used as received from commercial sources without further purification. Dichloromethane (DCM), anhydrous acetonitrile (MeCN), anhydrous methanol (MeOH), anhydrous tetrahydrofuran (THF), methyl methacrylate (MMA), 4-Chloro-7-nitrobenzofurazan (nitrobenzoxadiazole, NBD) chloride, 2,2'-azobis(2-methylpropionitrile) (AIBN), 2-hydroxyethyl methacrylate (HEMA), ethylene glycol methyl ether methacrylate (EGMEMA), 2,2,6,6-tetramethylpiperidin-1-yl)oxyl (TEMPO), 1-octanethiol, 4-(trifluoromethyl)benzaldehyde, tris(2-carboxyethyl)phosphine (TCEP), *p*-anisaldehyde, 3-mercaptopropanoic acid, *N*,*N*'-diisopropylcarbodiimide (DIC), 4-(dimethylamino)pyridine (DMAP), ethanolamine, cyanoacetic acid, 2-cyano-2-propyl benzodithioate, L-glutamic acid, L-tryptophan, lysozyme from chicken egg white, manganese chloride, sodium chloride, and sodium sulfate were purchased from Sigma-Aldrich (MO, USA). DMSO-d_6, acetone-d_6, and D_2O were purchased from Cambridge Isotopes Laboratories (MA, USA). Molecular sieves (13x, 4 to 8 mesh) were purchased from Acros Organics (Belgium). Benzaldehyde and resorufin were purchased from TCI America (OR, USA). 4-cyano-4-((phenylcarbonothioyl)thio)pentanoic acid was purchased from ChemScene (NJ, USA). Methyl cyanoacetate were purchased from ThermoFisher (MA, USA). Dimethyl sulfoxide (DMSO) was purchased from Fisher Chemical (NH, USA). Piperidine and 3.5 kDa MWCO dialysis tubing were purchased from Spectrum Chemical (CA, USA). Sulfo-Cyanine5 maleimide and sulfo-Cyanine5 amine were purchased from Lumiprobe (MD, USA). Ethyl alcohol was purchased from Gold Shield Distributors (CA, USA). Ammonium chloride, methanol and ethyl acetate were purchased from VWR Chemicals (PA, USA). Acetone, calcium chloride, iron chloride, and hydrogen peroxide were purchased from Matrix Scientific (SC, USA). PBS premix was purchased from Genesee Scientific (CA, USA). Yeast extract powder was purchased from MP Biomedicals (CA, USA). Casamino acid and magnesium sulfate were purchased from RPI (IL, USA). Amplex Red was purchased from Invitrogen (MA, USA).

S1.2. Instrumentations: A Horiba type LAQUAtwin-pH-22B compact pH meter was used for pH measurements. Absorption spectra and fluorescence spectra were recorded on a BioTek™ Synergy™ H1 Hybrid Multi-Mode microplate reader. Fluorescent microscope images were obtained on an RVL-100-M model ECHO Revolve fluorescence microscope at 60x magnification with a phase-contrast objective. Gel Permeation Chromatography (GPC) traces were obtained on a Waters Advanced Polymer Chromatography (APC) system using ACQUITY APC XT columns (125, 200, 450, 2.5 μm) with an RI detector. HPLC-grade THF was used as eluting solvent at a flow rate of 1.0 or 0.75 mL/min with column temperature at 25 °C. Number average and weight average molecular weight distributions of samples were measured with respect to polystyrene (PS) standards. Polymer samples for GPC were prepared by dissolving polymer powders in HPLC-grade THF at 3 mg/mL concentrations. DSC measurements were performed using TA Instruments Discovery DSC 2500. Rheology measurements were performed on a Discovery series HR-2 hybrid rheometer from TA instruments with an 8 mm diameter parallel plate geometry. Tensile testing experiments were performed on an Instron 3365 dual-column Universal Testing System (UTS) from INSTRON. SEM images were acquired from an FEI Magellan 400 XHR SEM at the UC Irvine Materials Research Institute (IMRI). Doubly distilled water was prepared using a Fisher Scientific Barnstead water purification system. Column chromatography was performed using a C-815 Flash instrument. Thin-layer chromatography was performed on Silicycle aluminum-backed TLC plates (Silica 200 *μ*m), and compounds were visualized using a UV lamp. Organic solvents were concentrated under reduced pressure using a rotary evaporator. Raman spectra were obtained at the Linear Spectroscopy Lab at UCI. High-resolution mass spectrometry spectra were obtained at the University of California Irvine Mass Spectrometry Facility. ^{1}H and $^{13}C\{^{1}H\}$ NMR spectra were obtained using Bruker instruments AV400, GN500, and AVANCE600 equipped with a cryoprobe. ^{1}H NMR spectra were acquired at 400 MHz, 500 MHz, or 600 MHz, $^{13}C\{^{1}H\}$ NMR spectra were acquired at 151 MHz, and all spectra were referenced to residual solvent signals (2.50 ppm for ^{1}H and 39.52 ppm for $^{13}C\{^{1}H\}$ DMSO peaks, and 2.09 ppm for ^{1}H acetone residual peaks). Peaks labeled with an asterisk (*) on ^{1}H NMR spectra are dichloromethane as a singlet at 5.76 ppm, methanol as a quartet at 4.01 ppm and a triplet at 3.16 ppm, water as a broad singlet at 3.33 ppm, DMSO as a septet at 2.50 ppm. Peaks labeled with an asterisk (*) on $^{13}C\{^{1}H\}$ NMR spectra in DMSO-d_6 are DMSO at 39.52 ppm.

S1.3. Methods

S1.3.1. Bacterial growth conditions: Saturated cultures of *B. subtilis* were prepared by inoculating a single colony into 5 mL of Lysogeny Broth (LB) at 37 °C and 250 rpm and growing for 9 h, followed by a 100x dilution using 495 mL of additional LB in a 2 L Erlenmeyer flask. The 500 mL culture was shaken at 37 °C and 250 rpm for 20 h, then centrifuged at 4700 rpm for 30 minutes. The supernatant was poured out, and the cell pellets were resuspended in 500 mL of SM medium (0.046 mg $FeCl_2$, 4.8 g $MgSO_4$, 12.6 mg $MnCl_2$, 535 mg NH_4Cl, 106 mg Na_2SO_4, 68 mg KH_2PO_4, 96.5 mg NH_4NO_3, 219 mg $CaCl_2$, 2 g L-glutamic acid, and 20 mg L-tryptophan, pH 7.1) and shaken at 37 °C and 250 rpm for 18 h according to the protocol established in our previous study.[S1] The mixture was then centrifuged at 4700 rpm for 30 minutes, and the resulting pellet was resuspended in a 0.1 mg/mL lysozyme solution in PBS (100 mM, pH 7.16) of 500 mL total volume. The lysozyme mixture was shaken at 37 °C and 250 rpm for 1 h and subsequently centrifuged at 4700 rpm for 30 minutes. The supernatant was poured out, and the spore pellet was washed five times with doubly distilled water and lyophilized to afford dry spore powder.

S1.3.2. Characterization of labeled *B. subtilis*: In microcentrifuge tubes, 95 μL suspensions of lyophilized spores (5 mg/mL) in DMSO were mixed with 5 μL DMSO solutions of fluorescent probes (120 μM for NBD–BCA–R, 125 μM for sulfoCyanine-5 dyes), and the resulting mixtures were incubated on a heating block at 25 °C and 800 rpm for 20 h. The mixtures were diluted with 1000 μL of DMSO and thoroughly mixed for 30 seconds, then centrifuged for 1 minute. The supernatants were poured out, and the spore pellets were resuspended in 1000 μL of DMSO followed by centrifugation for one minute, and the cycle was repeated five more times. The pellets were resuspended in 400 μL of DMSO, mixed, centrifuged, and 200 μL of the supernatant was taken out before thoroughly mixing the remaining 200 μL mixture. Bulk fluorescence spectroscopy (λ_{ex} = 460 nm and λ_{em} = 540 nm for NBD–BCA–R, λ_{ex} = 630 nm and λ_{em} = 650–700 nm for sulfoCyanine-5 dyes) and solution turbidity (OD_{600}) measurements were then taken of both the spore suspensions and the supernatants at 100 μL volumes. Fluorescence readouts from the supernatants were subtracted from that of the spore suspensions to account for background fluorescence. Spore labeling (normalized FL) was determined by dividing bulk fluorescence intensity (a.u.) values by the solution turbidity (OD_{600}) of each sample. Fluorescence (λ_{ex} = 488 nm and λ_{em} = 510 nm for NBD–BCA–R, λ_{ex} = 630 nm and λ_{em} = = 690–740 nm for sulfoCyanine-5 dyes) and optical microscopy images were also taken for each sample.

S1.3.3. Raman spectroscopy: Raman spectra were obtained by placing a 200 mM BCA–R and 1 mg/mL spore DMSO solution on a glass slide, focusing on the spore surface with a microscope, irradiating 785 nm light, and measuring the signal intensity of the resulting inelastic scattering. Presented spectra are average values of n = 5.

S1.3.4. Spore material preparation: Polymer powder (600 mg) was dissolved in 2 mL of DMSO. This polymer solution (200 μL) was added to a lyophilized *B. subtilis* spore (15 mg) in a 2 mL microcentrifuge tube so that the mass ratio of polymer to spore was 4 to 1. The resulting mixture was stirred vigorously for 5 minutes, incubated overnight, placed on a glass slide, and stored on the benchtop for another two days before wiping off any secreted DMSO for rheology studies. Dry biocomposite materials were prepared by repeating the same mixing procedure, transferring the DMSO mixture into a PTFE dog-bone mold, and letting the material sit overnight. The entire mold was then submerged in ethanol for two hours for solvent exchange, and the material was taken out of the mold and placed on a PTFE sheet for 24 hours to dry for tensile testing. For biocatalysis studies, the mixing procedure was repeated with APEX2 spores in acetone instead of DMSO and the material was transferred into water after overnight incubation in microcentrifuge tubes.

S1.3.5. Rheological characterizations: Linear rheological data (G' and G") were acquired at 1% strain amplitude with a frequency range of 0.1 to 600 rad/s (25 °C).

S1.3.6. Tensile testing: Dry materials were loaded on the Instron, pulled at a speed of 10 mm/min, and the stress response (MPa) was recorded against the strain (mm/mm).

S1.3.7. SEM imaging: Material fragments were sputter-coated with 5 nm of iridium. Images were acquired under immersion mode, dwell time 3.0 μs, integrated by 12 scans.

S1.3.8. Material disassembly: Materials were immersed in DMSO or acetone at 75 μL per mg of material. For materials that weren't lyophilized, the mixture was allowed to sit at ambient temperature for 8 hours. For materials lyophilized to obtain an accurate mass, DMSO mixtures were shaken at 250 rpm and 37 °C for one week. Lyophilized P4-based materials were transferred to a 1 L round bottom flask and further diluted to 0.2 mM per BCA to mimic ideal tM bond dissociation conditions determined by small molecule NMR studies (Figure S4.3). The mixture was then stirred at 1000 rpm and heated to 50 °C, and the extent of disassembly was monitored by microscopy while increasing the temperature by 10 °C increments every one to three days.

S1.3.9. Biocatalysis: Catalytic materials were soaked in 1 mL of water overnight, then placed into

a glass vial containing 1 mL of PBS (100 mM, pH 7.16) with 0.1 mM of Amplex Red and 0.8 mM of hydrogen peroxide. The vials were agitated at 250 rpm and 37 °C, and 100 μL aliquots of the supernatant were taken out at 30 min, 1 h, 2 h, 4 h, 1 day, 2 days, 5 days, 7 days, 8 days, 9 days, and 10 days (up to 33 days) to monitor conversion by fluorescence intensity (λ_{ex} = 530 nm, λ_{em} = 590 nm). For material reusability studies, the resorufin-stained TIED-APEX2 materials were washed by immersing them in 3 mL of PBS, shaking them at 37 °C and 250 rpm for one day, placing the material in a fresh vial containing 3 mL PBS, and repeating this cycle until the supernatant fluorescence of a final 1 mL PBS wash reached below 1% of maximum fluorescence observed in the biocatalysis. Free spore suspensions were prepared by creating 100 µL, 1 mg/mL solutions of the APEX2 spores in PBS, and fluorescence was measured by running the reaction in 96 well plates in the plate reader overnight at 37 °C and 205 cpm with measurements taken every 10 minutes. For reactions containing acetone, the mixtures were prepared at 150 µL in microcentrifuge tubes and shaken at 800 rpm and 37 °C for 48 hours before centrifuging, evaporating, lyophilizing, resuspending in 150 µL of PBS, and measuring fluorescence of the supernatant in 96-well plates.

S2. Synthesis and Characterization of Chemical Compounds

S2.1–S2.3. Synthesis and characterization of BCA–R

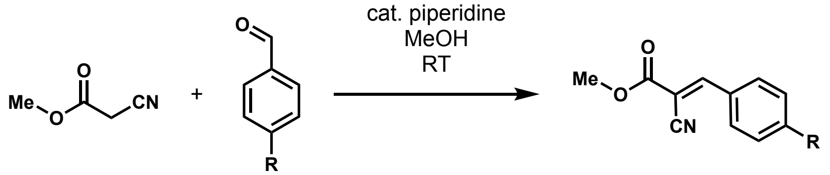

General procedure 1 (BCA-H). Following literature protocol with slight modifications,[S2] benzaldehyde (531 mg, 2.5 mmol, 1.0 equiv.), methyl cyanoacetate (500 mg, 5.0 mmol, 1.0 equiv.), and piperidine (cat.), were dissolved in anhydrous methanol (20 mL) in a 20 mL scintillation vial equipped with a micro stir bar. The reaction was stirred at room temperature overnight. The reaction mixture was then placed in a -20˚C freezer for 6 hours. The precipitate was vacuum-filtered and washed with methanol. The precipitate was then dried under reduced pressure to remove excess solvent to afford 393 mg of methyl-2-cyano-3-phenylacrylate as a white crystalline powder (42% isolated yield). ^{1}H NMR (600 MHz, DMSO) δ 8.43 (s, 1H), 8.06 (d, J = 7.7 Hz, 2H), 7.65 (t, J = 7.3 Hz, 1H), 7.60 (t, J = 7.2 Hz, 2H), 3.87 (s, 3H); ^{13}C{^{1}H} NMR (151 MHz, DMSO) δ 162.3, 155.2, 133.5, 131.4, 130.8, 129.4, 115.6, 102.4, 53.4; HRMS (TOF MS ES+) m/z calculated for $C_{11}H_9NO_2$ $[M+Na]^+$ 210.0531, found 210.0536.

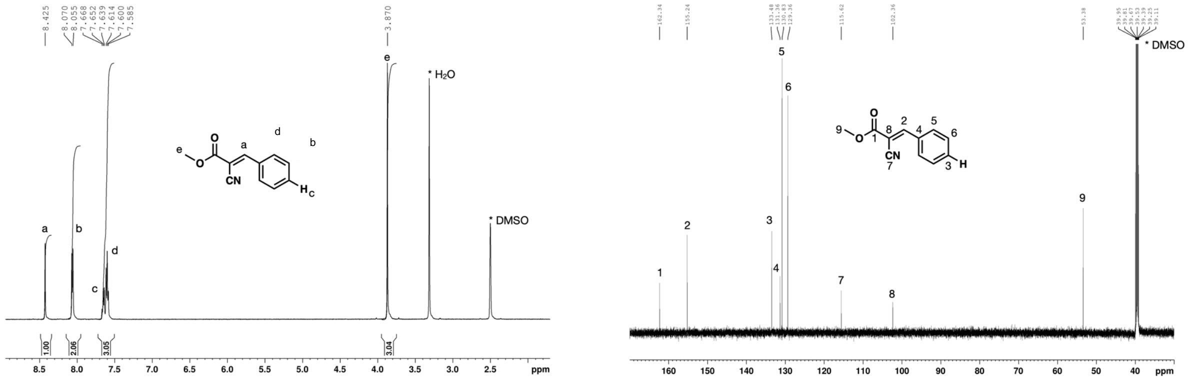

Figure S2.1 ^{1}H NMR (left) and ^{13}C{^{1}H} NMR (right) spectra of BCA–H in DMSO at 25 °C.

BCA-CF$_3$. Following **General procedure 1**, 535 mg of methyl-2-cyano-3-(4-(trifluoromethyl)phenyl)acrylate was isolated as a white crystalline solid (42% yield). ^{1}H NMR (600 MHz, DMSO) δ 8.54 (s, 1H), 8.22 (d, *J* = 8.1 Hz, 2H), 7.98 (d, *J* = 8.2 Hz, 2H), 3.89 (s, 3H); ^{13}C{^{1}H} NMR (151 MHz, DMSO) δ 161.9, 153.6, 135.2, 132.1, 131.2, 126.1, 123.7, 115.1, 105.5, 53.6; HRMS (TOF MS ES+) m/z calculated for $C_{12}H_8F_3NO_2$ [M+Na]$^+$ 278.0405, found 278.0407.

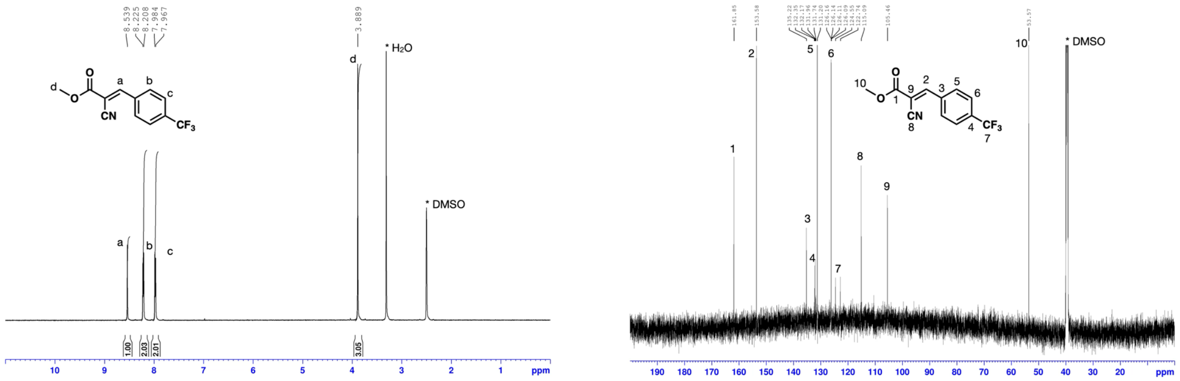

Figure S2.2 ^{1}H NMR (left) and ^{13}C{^{1}H} NMR (right) spectra of BCA–CF$_3$ in DMSO at 25 °C.

BCA-OMe. Following **General procedure 1**, 757 mg of methyl-2-cyano-3-(4-methoxyphenyl)acrylate was isolated as a white crystalline solid (70% yield). ^{1}H NMR (500 MHz, DMSO) δ 8.33 (s, 1H), 8.10 (d, *J* = 8.7 Hz, 2H), 7.16 (d, *J* = 8.8 Hz, 2H), 3.87 (s, 3H), 3.84 (s, 3H); ^{13}C{^{1}H} NMR (151 MHz, DMSO) δ 163.6, 162.9, 154.6, 133.6, 124.0, 116.3, 115.0, 98.3, 55.8, 53.2; HRMS (TOF MS ES+) m/z calculated for $C_{12}H_{11}NO_3$ [M+Na]$^+$ 240.0637, found 240.0628.

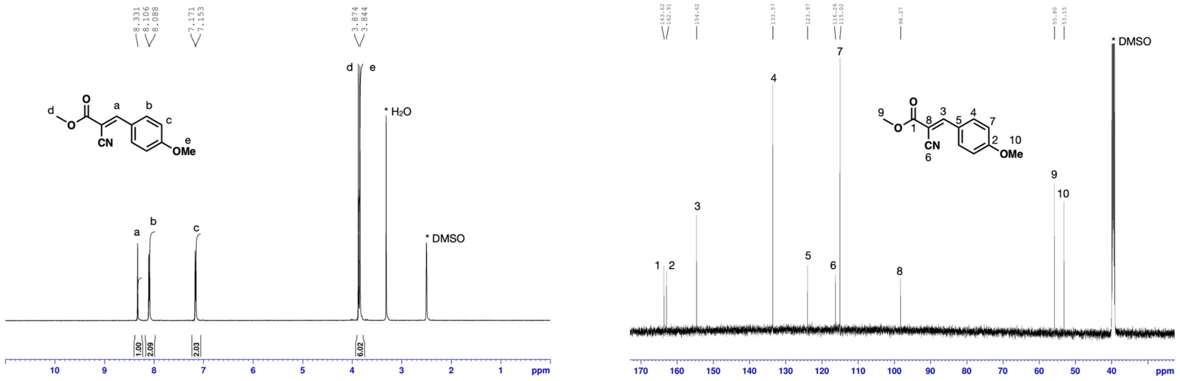

Figure S2.3 ^{1}H NMR (left) and ^{13}C{^{1}H} NMR (right) spectra of BCA–OMe in DMSO at 25 °C.

S2.4–S2.10. Synthesis and characterization of NBD–BCA–R

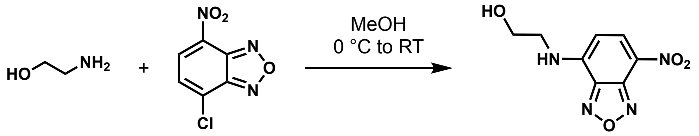

NBD–OH. Following literature protocol with slight modifications,[S3] 4-Chloro-7-nitrobenzofurazan (nitrobenzoxadiazole, NBD) chloride (1960 mg, 9.82 mmol, 1.0 equiv) was dissolved in anhydrous methanol (98 mL) in a 250 mL round bottom flask equipped with a stir bar and cooled to 0 °C. Ethanolamine (1.2 mL, 19.64 mmol, 2.0 equiv) was added dropwise at 0 °C, and the reaction mixture was stirred at room temperature for 20 hours. The solvent was removed under reduced pressure, and the residue was dissolved in ethyl acetate. The organic phase was then washed with ammonium chloride (x 5) and brine (x 1), dried with sodium sulfate, and concentrated under reduced pressure to afford 1168 mg of 2-((7-nitrobenzo[*c*][1,2,5]oxadiazol-4-yl)amino)ethan-1-ol as a black powder (53% isolated yield). ^{1}H NMR (600 MHz, DMSO) δ 9.42 (s, 1H), 8.51 (d, *J* = 8.6 Hz, 1H), 6.45 (d, *J* = 8.7 Hz, 1H), 4.94 (s, 1H), 3.69 (q, *J* = 5.6 Hz, 2H), 3.55 (s, 2H); ^{13}C{^{1}H} NMR (151 MHz, DMSO) δ 145.7, 144.5, 144.1, 138.0, 120.5, 99.4, 58.9, 46.1. HRMS (TOF MS ES-) m/z calculated for $C_8H_8N_4O_4$ $[M+H]^+$ 225.0624, found 225.0614.

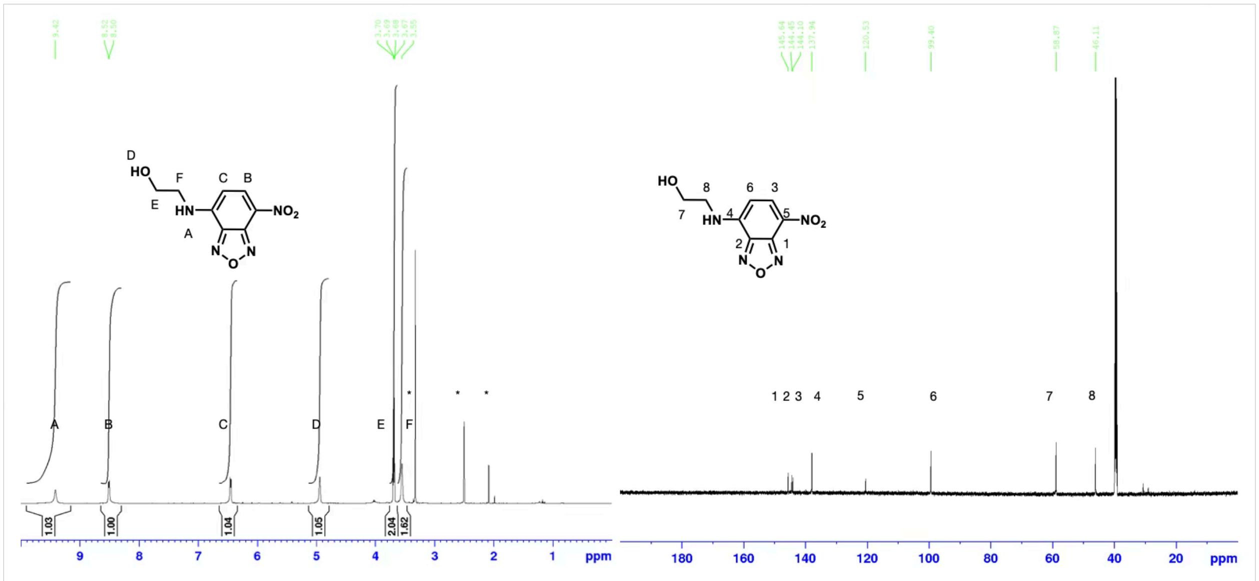

Figure S2.4 ^{1}H NMR (left) and ^{13}C{^{1}H} NMR (right) spectra of NBD–OH in DMSO at 25 °C.

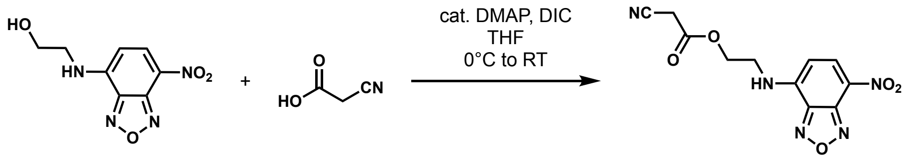

NBD–CN. NBD–OH (819 mg, 3.65 mmol, 1.0 equiv) and cyanoacetic acid (310 mg, 3.65 mmol, 1.0 equiv) were dissolved in anhydrous THF (6 mL) in a flame-dried 25 mL round bottom flask equipped with a stir bar and cooled to 0 °C. DIC (0.6 mL, 4.02 mmol, 1.1 equiv) and DMAP (45 mg, 0.37 mmol, 0.1 equiv) were sequentially added dropwise at 0 °C while stirring. Then reaction was allowed to warm to room temperature and stirred for 24 hours. The solvent was removed under reduced pressure, and the residue was dissolved in DCM. The organic phase was washed with sodium bicarbonate (x 4) and brine (x 2), dried with sodium sulfate, and concentrated under reduced pressure to afford 999 mg of 2-((7-nitrobenzo[*c*][1,2,5]oxadiazol-4-yl)amino)ethyl 2-cyanoacetate as a black powder (94% isolated yield). ^{1}H NMR (600 MHz, DMSO) δ 9.46 (s, 1H), 8.53 (d, *J* = 8.8 Hz, 1H), 6.51 (d, *J* = 8.9 Hz, 1H), 4.41 (t, *J* = 5.3 Hz, 2H), 3.98 (s, 2H), 3.79 (bs, 2H); $^{13}C\{^{1}H\}$ NMR (151 MHz, DMSO) δ 164.4, 156.8, 144.4, 144.1, 137.9, 121.4, 114.9, 99.6, 63.5, 40.7, 24.6. HRMS (TOF MS ES-) m/z calculated for $C_{11}H_{9}N_{5}O_{5}$ $[M-H]^{-}$ 290.0526, found 290.0524.

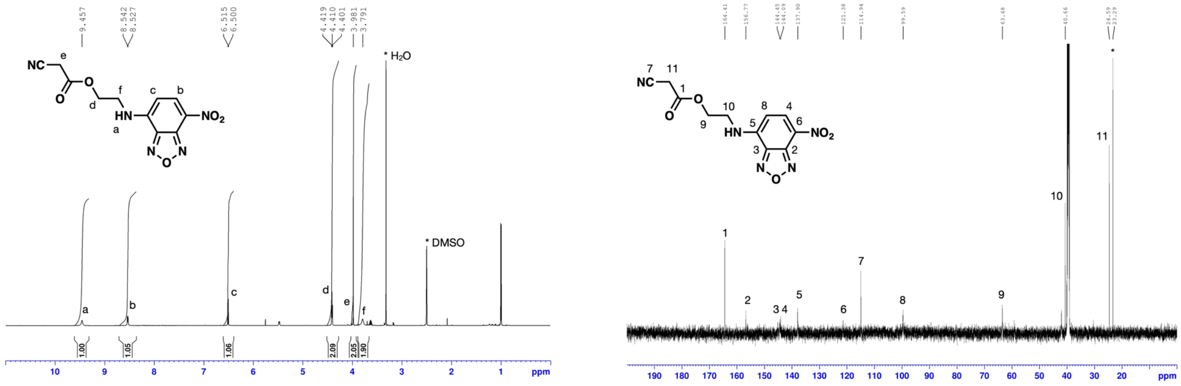

Figure S2.5 ^{1}H NMR (left) and $^{13}C\{^{1}H\}$ NMR (right) spectra of NBD–CN in DMSO at 25 °C.

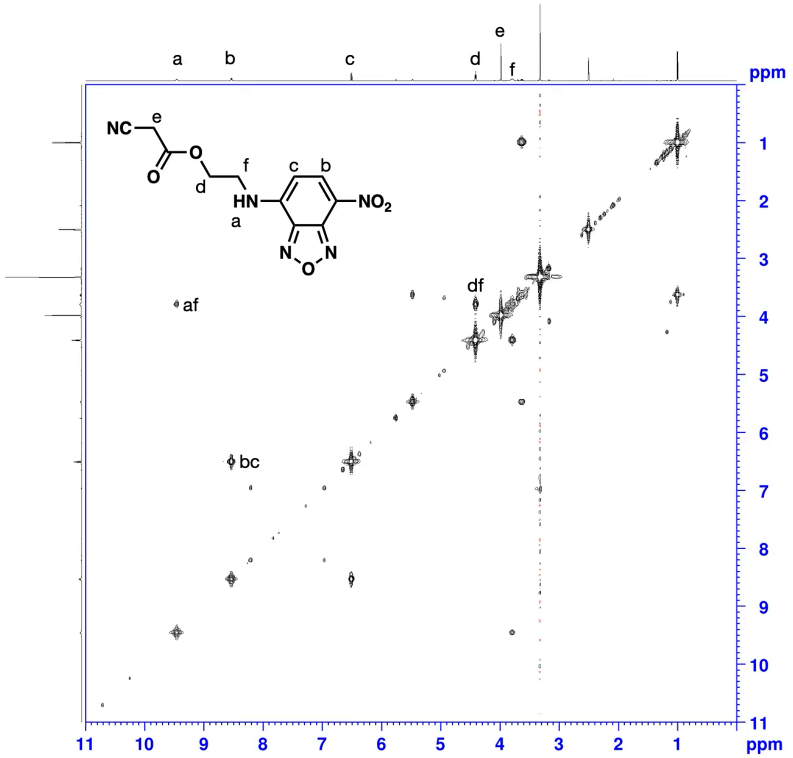

Figure S2.6 $^1H–^1H$ COSY NMR spectrum of NBD-CN in DMSO at 25 °C

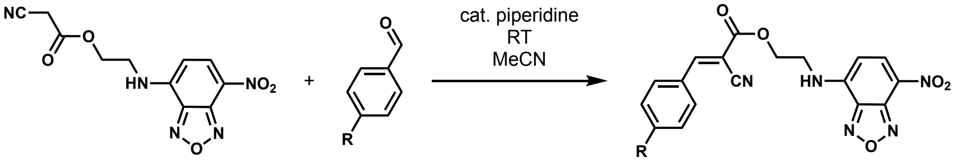

General Procedure 2 (NBD–BCA–CF_3). NBD–CN (100 mg, 0.69 mmol, 1.0 equiv), 4-(trifluoromethyl)benzaldehyde (0.06 mL, 0.41 mmol, 1.2 equiv), and catalytic piperidine were dissolved in anhydrous acetonitrile (7 mL) in a 20 mL vial equipped with activated molecular sieves and a stir bar. The mixture was stirred at room temperature for 24 hours, filtered to remove molecular sieves, dried with sodium sulfate, and concentrated under reduced pressure. The crude mixture was purified by flash column chromatography (SiO_2, 0–100% ethyl acetate in hexane gradient) to afford 37 mg of 2-((7-nitrobenzo[*c*][1,2,5]oxadiazol-4-yl)amino)ethyl (*E*)-2-cyano-3-(4-(trifluoromethyl)phenyl)acrylate as an orange powder (24% isolated yield). ^{1}H NMR (600 MHz, DMSO) δ 9.52 (s, 1H), 8.50 (app. s, 1H), 8.49 (s, 1H), 8.16 (d, *J* = 8.2 Hz, 2H), 7.94 (d, *J* = 8.3 Hz, 2H), 6.57 (d, *J* = 8.8 Hz, 1H), 4.58 (t, *J* = 5.1 Hz, 2H), 3.90 (bs, 2H); ^{13}C{^{1}H} NMR (151 MHz, DMSO) δ 161.4, 153.7, 145.1, 144.5, 144.0, 137.8, 135.1, 132.2 (*J* = 32.2 Hz), 126.3, 126.1 (*J* = 3.7 Hz), 123.6 (*J* = 272.7 Hz), 121.4, 114.9, 105.4, 99.7, 64.3, 42.0. HRMS (TOF MS ES+) m/z calculated for $C_{19}H_{12}F_3N_5O_5$ [M+H]$^+$ 448.0869, found 448.0859.

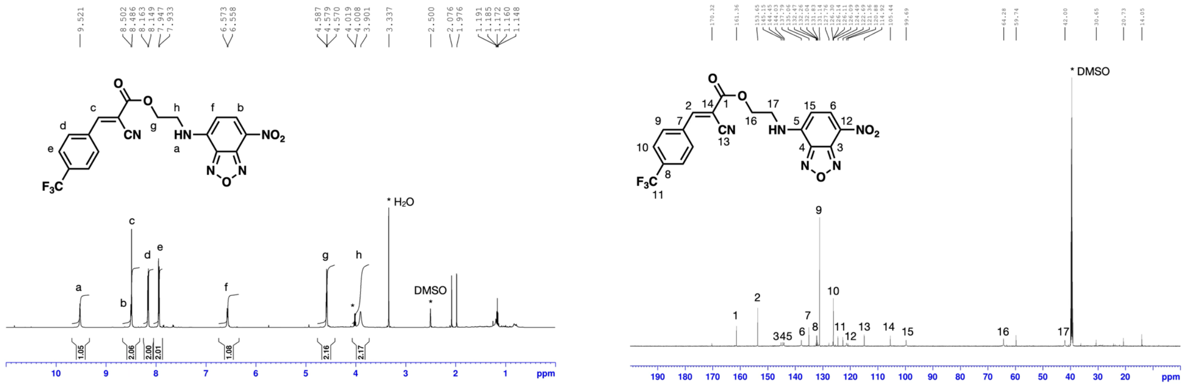

Figure S2.7 ^{1}H NMR (left) and ^{13}C{^{1}H} NMR (right) spectra of NBD–BCA–CF_3 in DMSO at 25 °C.

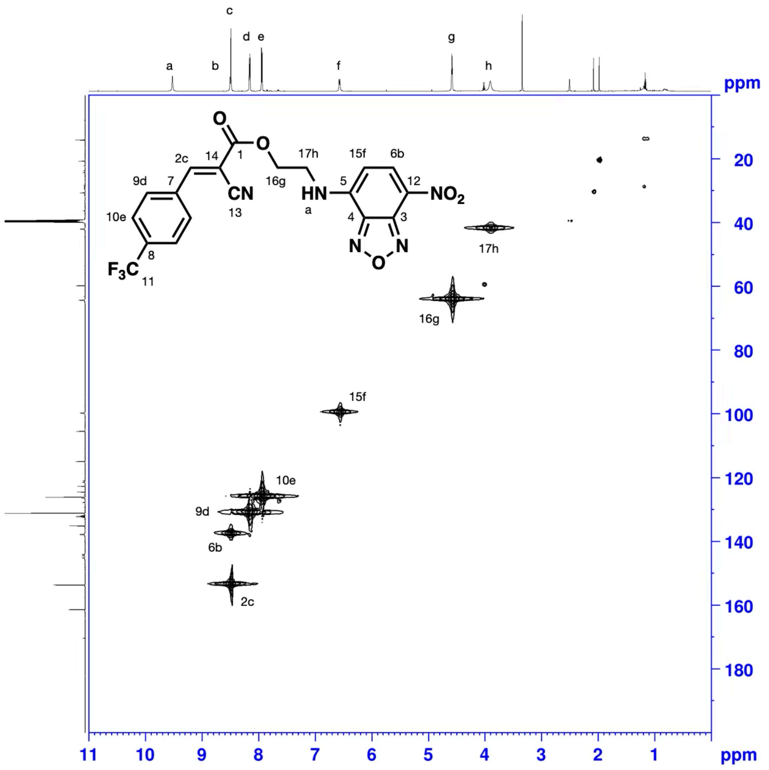

Figure S2.8 ^{1}H–^{13}C HMQC NMR spectrum of NBD–BCA–CF_3 in DMSO at 25 °C

NBD–BCA–H. Following **General procedure 2**, 38 mg of 2-((7-nitrobenzo[*c*][1,2,5]oxadiazol-4-yl)amino)ethyl (*E*)-2-cyano-3-phenylacrylate was isolated as an orange powder (14% yield). ^{1}H NMR (600 MHz, DMSO) δ 9.52 (s, 1H), 8.49 (app. d, *J* = 8.7 Hz, 1H), 8.36 (s, 1H), 7.99 (d, *J* = 7.7 Hz, 2H), 7.63 (app. t, *J* = 7.3 Hz, 1H), 7.57 (app. t, *J* = 7.6 Hz, 2H), 6.56 (app. d, *J* = 8.6 Hz, 1H), 4.56 (app. t, *J* = 4.8 Hz, 2H), 3.89 (s, 2H); $^{13}C\{^{1}H\}$ NMR (151 MHz, DMSO) δ 161.9, 155.3, 145.2, 144.4, 137.8, 133.5, 131.2, 130.8, 129.2, 128.7, 115.4, 102.6, 102.4, 99.7, 64.1, 42.0. HRMS (TOF MS ES+) m/z calculated for $C_{18}H_{13}N_{5}O_{5}$ [M+H]$^{+}$ 380.0995, found 380.1005.

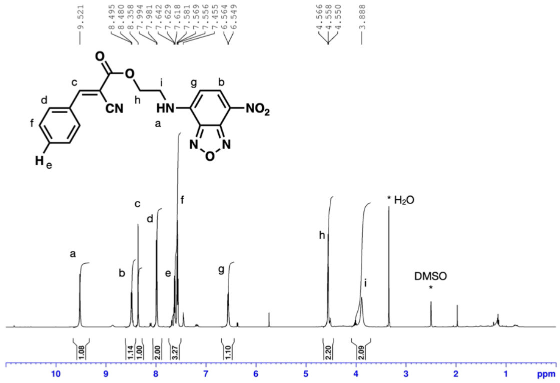

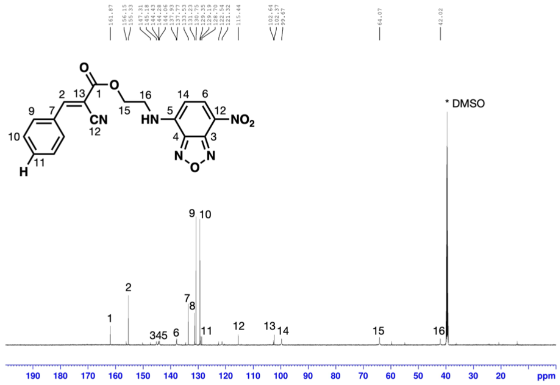

Figure S2.9 ^{1}H NMR (left) and $^{13}C\{^{1}H\}$ NMR (right) spectra of NBD–BCA–H in DMSO at 25 °C.

NBD–BCA–OMe. Following **General procedure 2**, 38 mg of 2-((7-nitrobenzo[*c*][1,2,5]oxadiazol-4-yl)amino)ethyl (*E*)-2-cyano-3-(4-methoxyphenyl)acrylate was isolated as an orange powder (27% yield). ^{1}H NMR (600 MHz, DMSO) δ 9.54 (s, 1H), 8.52 (d, *J* = 8.8 Hz, 1H), 8.28 (s, 1H), 8.04 (app. d, *J* = 8.8 Hz, 2H), 7.15 (app. d, *J* = 9.0 Hz, 2H), 6.58 (app. d, *J* = 8.8 Hz, 1H), 4.54 (t, *J* = 5.2 Hz, 2H), 4.09–3.77 (bs, 5H); ^{13}C{^{1}H} NMR (151 MHz, DMSO) δ 163.7, 162.5, 154.8, 145.2, 144.5, 144.1, 137.9, 133.5, 123.9, 121.3, 116.1, 115.0, 99.8, 98.3, 63.8, 55.8, 42.1. HRMS (TOF MS ES+) m/z calculated for $C_{19}H_{15}N_5O_6$ [M+Na]$^+$ 432.0920, found 432.0922.

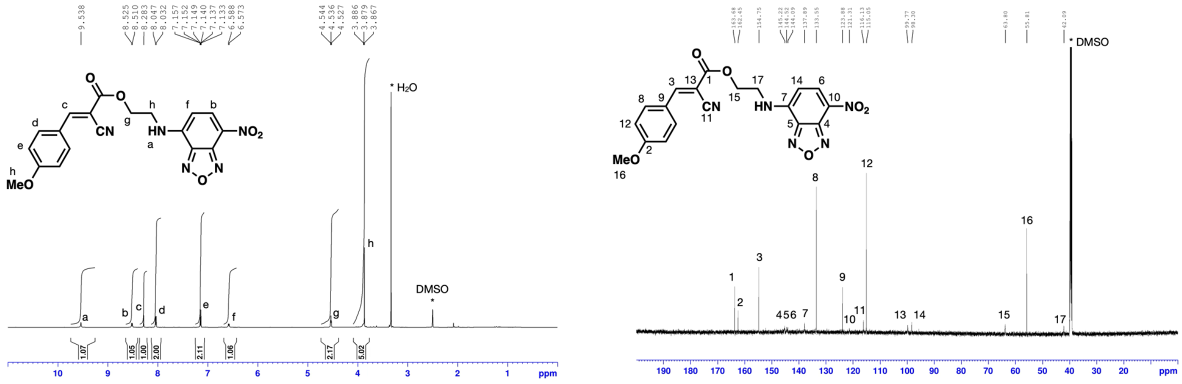

Figure S2.10 ^{1}H NMR (left) and ^{13}C{^{1}H} NMR (right) spectra of NBD–BCA–H in DMSO at 25 °C.

S2.11–S2.19. Synthesis and characterization of BCAMA–R

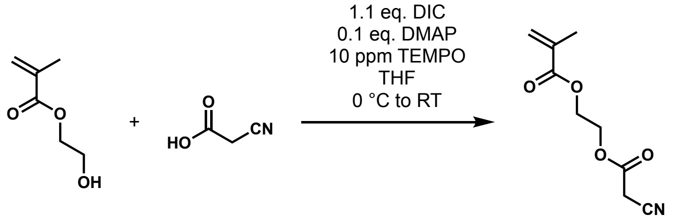

HEMA–CN. HEMA (18.67 mL, 153.68 mmol, 1.0 equiv), cyanoacetic acid (1307.39 mg, 153. 68 mmol, 1.0 equiv), and TEMPO (24.01 mg, 0.15 mmol, 0.001 equiv) were dissolved in anhydrous THF (200 mL) in a 500 mL flame dried round bottom flask equipped with a stir bar and cooled to 0 °C. DIC (26.17 mL, 169.07 mmol, 1.1 equiv) and DMAP (1877.86 mg, 15.37 mmol, 0.1 equiv) dissolved in THF (20 mL) were sequentially added dropwise at 0 °C, and the reaction mixture was allowed to warm to room temperature and stirred for 20 hours. The mixture was then filtered to remove the diisopropylurea byproduct, concentrated under reduced pressure, and dissolved in DCM. The organic phase was washed with doubly distilled water (x 2), sodium bicarbonate (x1), brine (x 1), dried with sodium sulfate, and concentrated to afford 3030.24 mg of 2-(2-cyanoacetoxy)ethyl methacrylate as an orange oil (99% isolated yield). ^{1}H NMR (498 MHz, DMSO) δ 6.05 (s, 1H), 5.70 (t, *J* = 1.5 Hz, 1H), 4.39 (app. dt, *J* = 4.5, 3.0 Hz, 2H), 4.31 (app. dt, *J* = 4.5, 3.0, 2H), 4.04 (s, 2H), 1.88 (s, 3H); ^{13}C{^{1}H} NMR (125 MHz, DMSO) δ 166.4, 164.3, 135.5, 126.2, 114.9, 63.6, 62.1, 24.4, 17.9. HRMS (TOF MS ES-) m/z calculated for $C_9H_{11}NO_4$ $[M-H]^-$ 196.0610, found 196.0605.

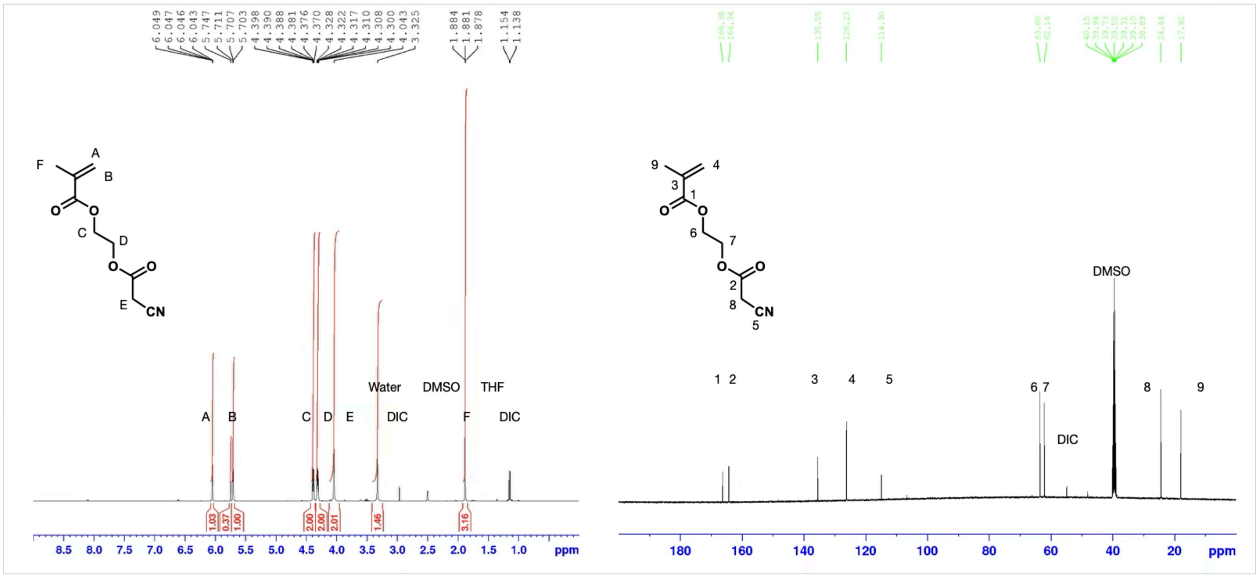

Figure S2.11 ^{1}H NMR (left) and ^{13}C{^{1}H} NMR (right) spectra of HEMA–CN in DMSO at 25 °C.

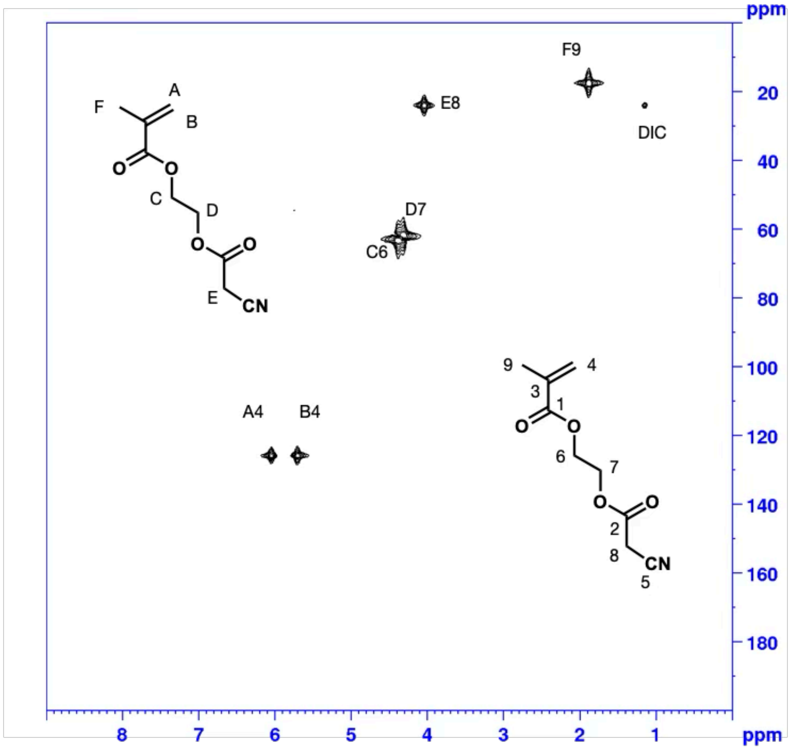

Figure S2.12 ^{1}H–^{13}C HMQC NMR spectrum of HEMA–CN in DMSO at 25 °C

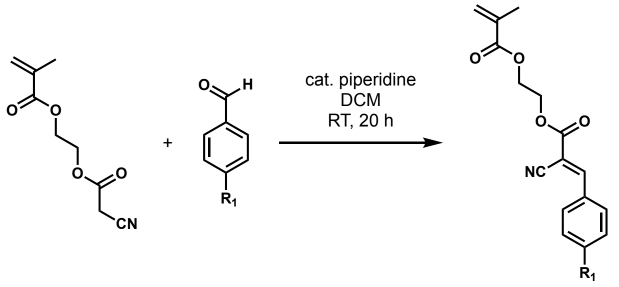

General Procedure 3 (BCAMA–CF_3). HEMA–CN (3707.17 mg, 188.00 mmol, 1.0 equiv), 4-(trifluoromethyl)benzaldehyde (28.24 mL, 206.80 mmol, 1.1 equiv), and 3 drops of piperidine (cat.) were dissolved in DCM (470 mL) in a 1000 mL round bottom flask equipped with a stir bar and stirred at room temperature for 20 hours. A saturated aqueous solution of sodium bisulfite (470 mL) was added, and the mixture was stirred for an additional 4 hours. The two layers were then separated, and the organic layer was washed with water (x 2), ammonium chloride (x 4), brine (x 2), dried with sodium sulfate, and concentrated under reduced pressure to afford 4286.90 mg of 2-(methacryloyloxy)ethyl (*E*)-2-cyano-3-(4-(trifluoromethyl)phenyl)acrylate as a yellow crystalline powder (65 % isolated yield). ^{1}H NMR (600 MHz, DMSO) δ 8.54 (s, 1H), 8.21 (d, *J* = 8.3 Hz, 2H), 7.97 (d, *J* = 8.3 Hz, 2H), 6.06 (app. q, *J* = 0.8 Hz, 1H), 5.71 (app. dt, *J* = 3.0, 1.5 Hz, 1H), 4.56 (dt, *J* = 3.6, 2.2 Hz, 2H), 4.42 (dt, *J* = 3.6, 2.3 Hz, 2H), 1.88 (app. t, *J* = 1.1 Hz, 3H); ^{13}C{^{1}H} NMR (151 MHz, DMSO) δ 166.5, 161.3, 153.9, 135.6, 135.2, 132.2 (q, *J* = 32.2 Hz), 131.3, 126.3, 126.2 (q, *J* = 3.6 Hz), 123.7 (q, *J* = 272.6 Hz), 115.0, 105.4, 64.3, 62.1, 18.0; HRMS (TOF MS ES+) m/z calculated for $C_{17}H_{14}F_3NO_4$ $[M+Na]^+$ 376.0773, found 376.0776.

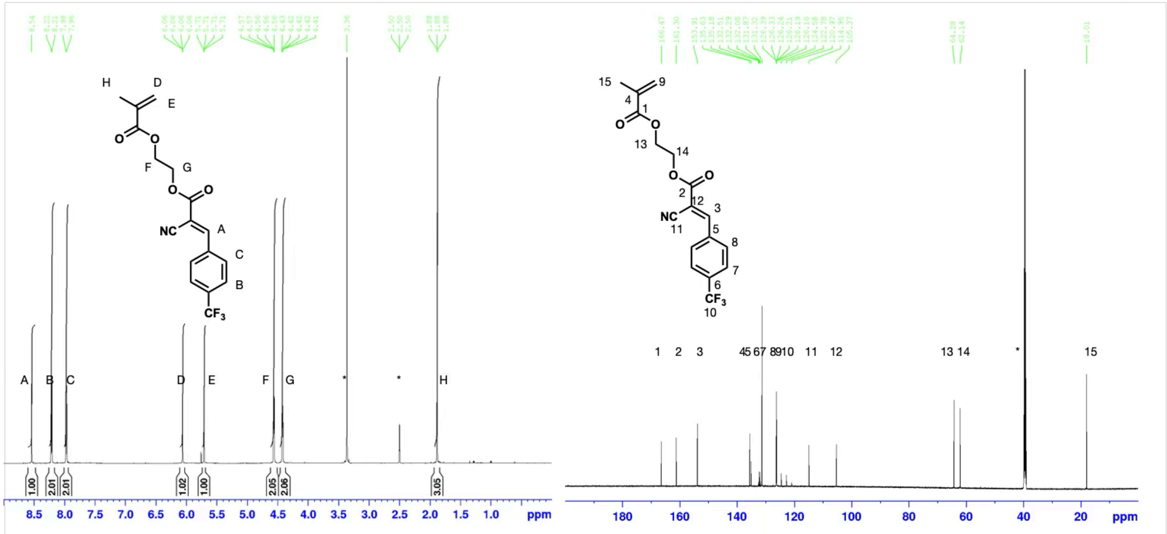

Figure S2.13 ^{1}H NMR (left) and ^{13}C{^{1}H} NMR (right) spectra of NBD-H in DMSO at 25 °C.

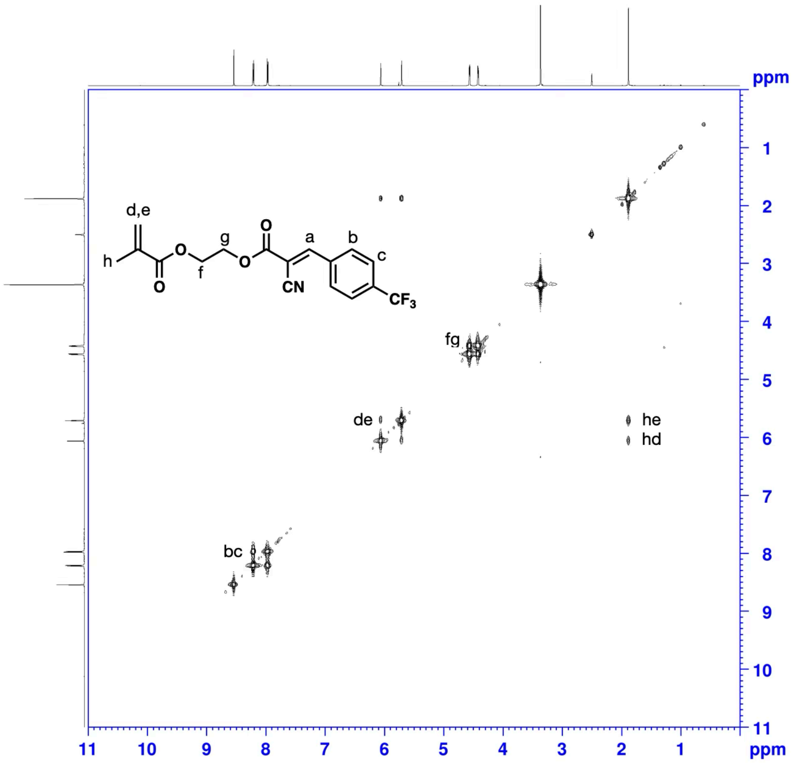

Figure S2.14 ^{1}H–^{1}H COSY NMR spectrum of BCAMA–CF_3 in DMSO at 25 °C

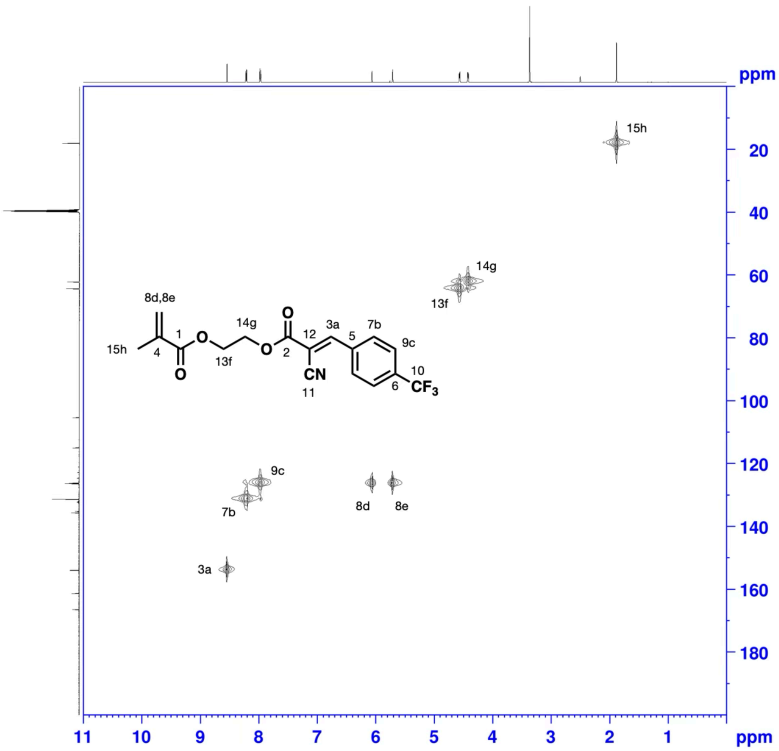

Figure S2.15 ^{1}H–^{13}C HMQC NMR spectrum of BCAMA–CF_3 in DMSO at 25 °C

BCAMA–H. Following **General Procedure 3**, 1716.70 mg of 2-(methacryloyloxy)ethyl (*E*)-2-cyano-3-phenylacrylate was isolated as an orange solid (76% yield). ^{1}H NMR (600 MHz, DMSO) δ 8.43-8.39 (m, 1H), 8.08-8.02 (m, 2H), 7.68-7.63 (m, 1H), 7.63-7.56 (m, 2H), 6.06 (app. s, 1H), 5.71 (app. s, 1H), 4.55 (app. s, 2H), 4.42 (app. s, 2H), 1.88 (app. s, 3H); ^{13}C{^{1}H} NMR (151 MHz, DMSO) δ 166.4, 161.7, 155.5, 135.6, 133.6, 131.3, 130.9, 129.4, 126.2, 115.4, 102.3, 64.0, 62.1, 17.9; HRMS (TOF MS ES+) m/z calculated for $C_{16}H_{15}NO_4$ $[M+Na]^+$ 308.0899, found 308.0888.

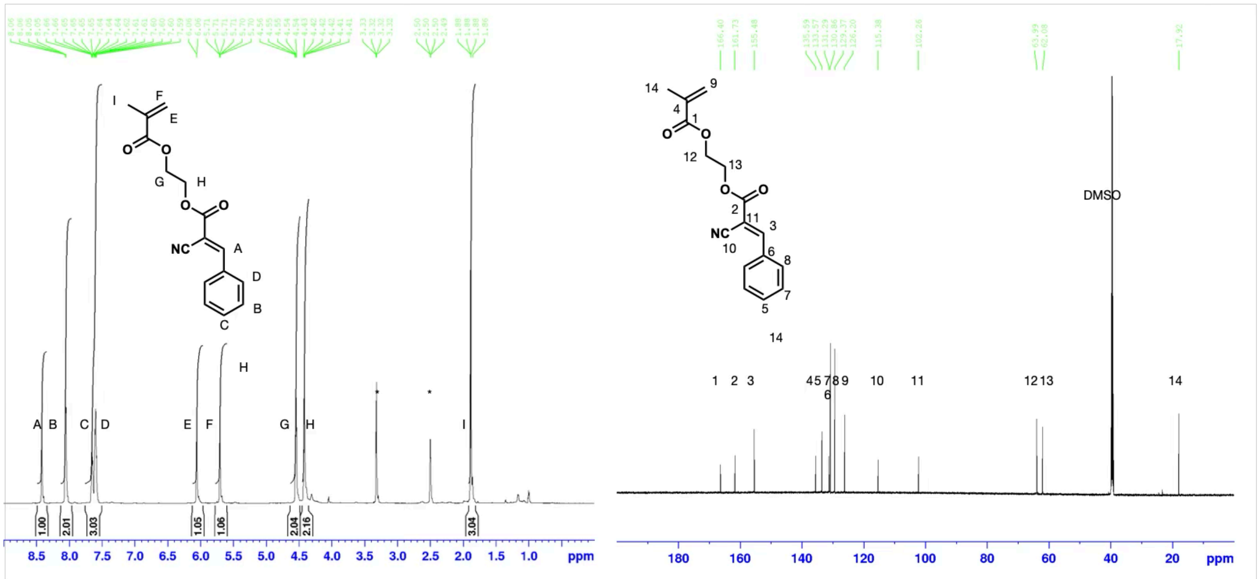

Figure S2.16 ^{1}H NMR (left) and ^{13}C{^{1}H} NMR (right) spectra of BCAMA–H in DMSO at 25 °C.

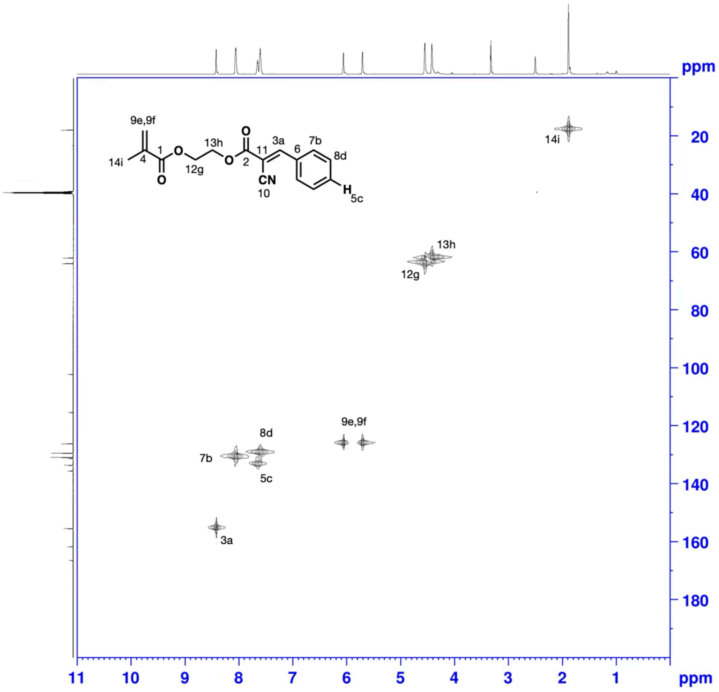

Figure S2.17 ^{1}H–^{13}C HMQC NMR spectrum of BCAMA–H in DMSO at 25 °C.

BCAMA–OMe. Following **General Procedure 3**, 1574.50 mg of 2-(methacryloyloxy)ethyl (*E*)-2-cyano-3-(4-methoxyphenyl)acrylate was isolated as a white powder (68% yield). ^{1}H NMR (600 MHz, DMSO) δ 8.28 (s, 1H), 8.06 (app. d, *J* = 8.9 Hz, 2H), 7.12 (app. d, *J* = 8.9 Hz, 1H), 6.05 (s, 1H), 5.68 (app t, *J* = 1.6 Hz, 1H), 4.55-4.48 (m, 2H), 4.43-4.38 (m, 1H), 3.86 (s, 3H), 3.35 (s, 1H), 1.87 (s, 3H); ^{13}C{^{1}H} NMR (151 MHz, DMSO) δ 166.4, 163.7, 162.3, 154.7, 135.6, 133.6, 126.1, 123.9, 116.0, 115.0, 98.1, 63.7, 62.1, 55.7, 17.9; HRMS (TOF MS ES+) m/z calculated for $C_{17}H_{17}NO_5$ [M+Na]$^+$ 338.1004, found 338.1005.

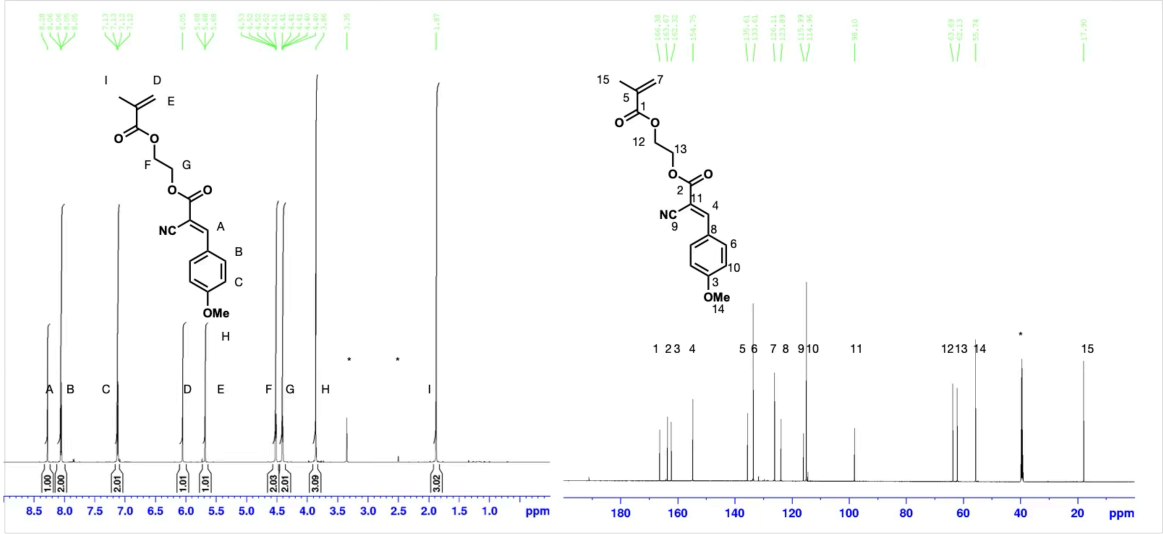

Figure S2.18 ^{1}H NMR (left) and ^{13}C{^{1}H} NMR (right) spectra of BCAMA–OMe in DMSO at 25 °C.

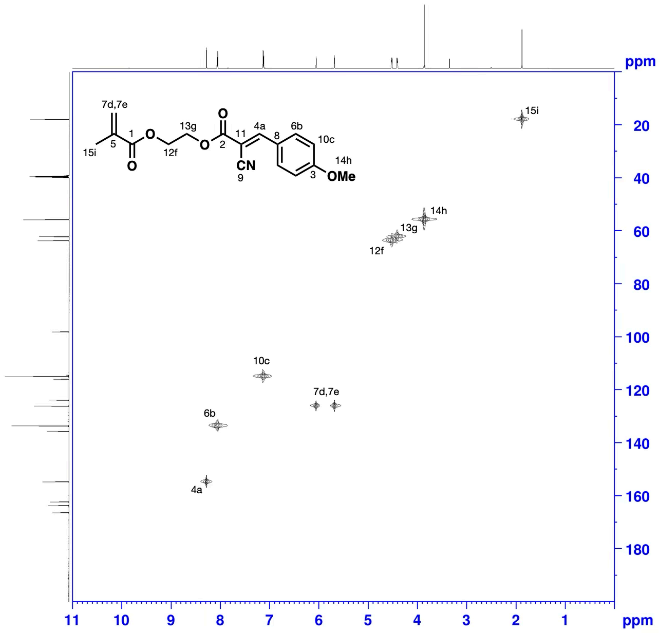

Figure S2.19 ^{1}H–^{13}C HMQC NMR spectrum of BCAMA–H in DMSO at 25 °C.

S2.20–S2.32 Synthesis and characterization of polymers P# [B (or N)-R_1-R_2-%n]

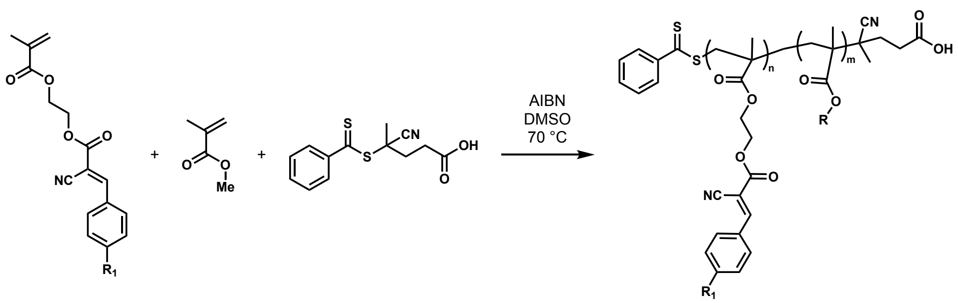

General Procedure 4 [P1 (N-CF_3-CH_3-10)]. BCAMA–CF_3 (707 mg, 2.00 mmol, 10 equiv) and MMA (1.93 mL, 18.00 mmol, 90 equiv) were dissolved in DMSO (9.38 mL) in a 20 mL vial. A 50 mg/mL solution of AIBN in DMSO (0.26 mL, 0.08 mmol, 0.4 equiv) and a 95 mg/mL solution of 4-cyano-4-((phenylcarbonothioyl)thio)pentanoic acid (2.35 mL, 0.80 mmol, 4 quiv) were added and the vial was sealed using a rubber septum. Nitrogen gas was bubbled into the solution for 1 hour, and the reaction was heated to 70 °C for 1 day. The mixture was then poured into a 3.5 kDa MWCO dialysis tubing and dialyzed for 1 week against acetone, exchanging the solvent once a day. The acetone was evaporated under reduced pressure to afford P1 as a pink powder. ^{1}H NMR (400 MHz, DMSO) δ 12.28 (bs), 8.56 (bs), 8.23 (bs), 7.98 (bs), 7.84 (bs), 7.63 (app. t, J = 6.8 Hz), 7.47 (app. t, J = 6.8 Hz), 4.54 (bs), 4.23 (bs), 3.54 (bs), 2.04–1.36 (bs), 1.08–0.61 (bs). $M_{n,calcd.}$ = 5,197 g/mol, $M_{n,\,GPC}$ = 4,064 g/mol, $M_w/M_{n,GPC}$ = 1.09.

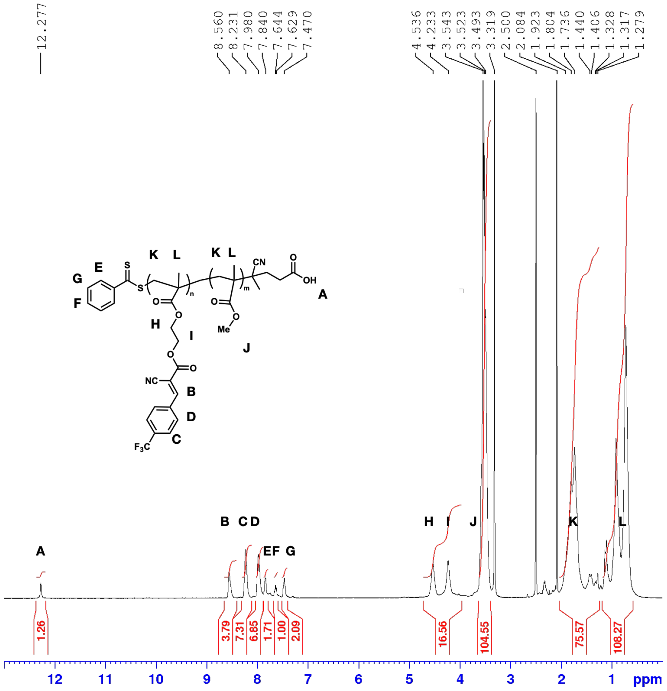

Figure S2.20 ^{1}H NMR spectrum of P1 in DMSO at 25 °C.

P2 (N-OMe-CH_3-10). Following **General Procedure 4**, P2 was isolated as a pink powder. ^{1}H NMR (400 MHz, DMSO) δ 12.29 (bs), 8.34 (bs), 8.11 (bs), 7.84 (bs), 7.65 (app. t, J = 7.3 Hz), 7.47 (app. t, J = 7.3 Hz), 7.17 (bs), 4.49 (bs), 4.22 (bs), 3.89 (bs), 3.54 (bs), 2.04–1.36 (bs), 1.08–0.61 (bs). $M_{n,calcd.}$ = 4,845 g/mol, $M_{n,\,GPC}$ = 4,200 g/mol, $M_w/M_{n,GPC}$ = 1.09.

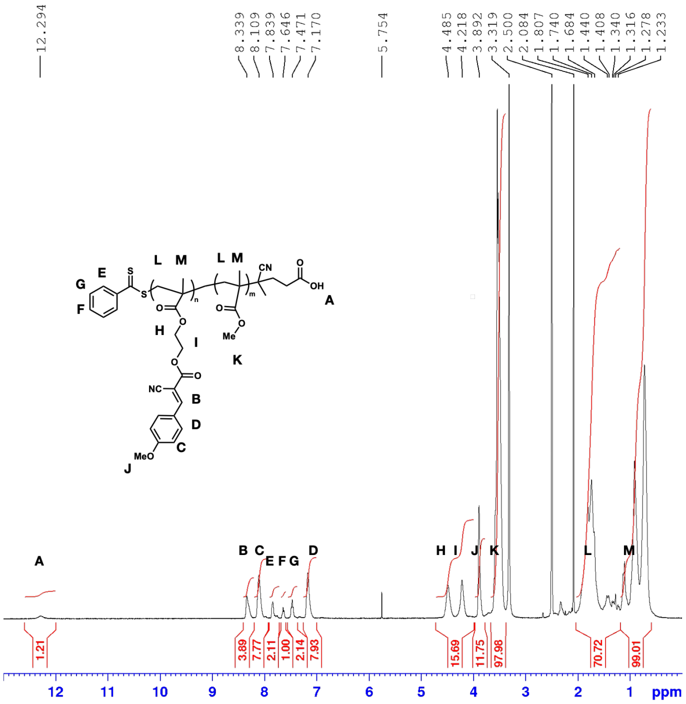

Figure S2.21 ^{1}H NMR spectrum of P2 in DMSO at 25 °C.

General Procedure 5 (P1–NBD). P1 (500 mg, 0.12 mmol, 1.0 equiv.) and NBD–OH (50 mg, 0.22 mmol, 1.9 equiv.) were dissolved in anhydrous THF (12 mL) in a 20 mL vial equipped with a stir bar and cooled to 0 °C. DIC (0.06 mL, 0.41 mmol, 3.38 equiv) and DMAP (5 mg, 0.04 mmol, 0.34 equiv) dissolved in 1 mL THF were sequentially added dropwise at 0 °C and the mixture was allowed to warm to room temperature and stirred for 1 day. The mixture was then poured into a 3.5 kDa MWCO dialysis tubing and dialyzed for 1 week against acetone, exchanging the solvent once a day. The acetone was evaporated under reduced pressure to afford P1–NBD as an orange powder. ^{1}H NMR (400 MHz, DMSO) δ 9.47 (bs), 8.56 (bs), 8.23 (bs), 7.98 (bs), 7.84 (bs), 7.64 (app. t, J = 7.2 Hz), 7.47 (app. t, J = 7.2 Hz), 6.50 (app. d, J = 8.4 Hz), 4.55 (bs), 4.33 (bs), 4.23 (bs), 3.78 (bs), 3.54 (bs), 2.04–1.36 (bs), 1.08–0.61 (bs). $M_{n,calcd.}$ = 5,303 g/mol.

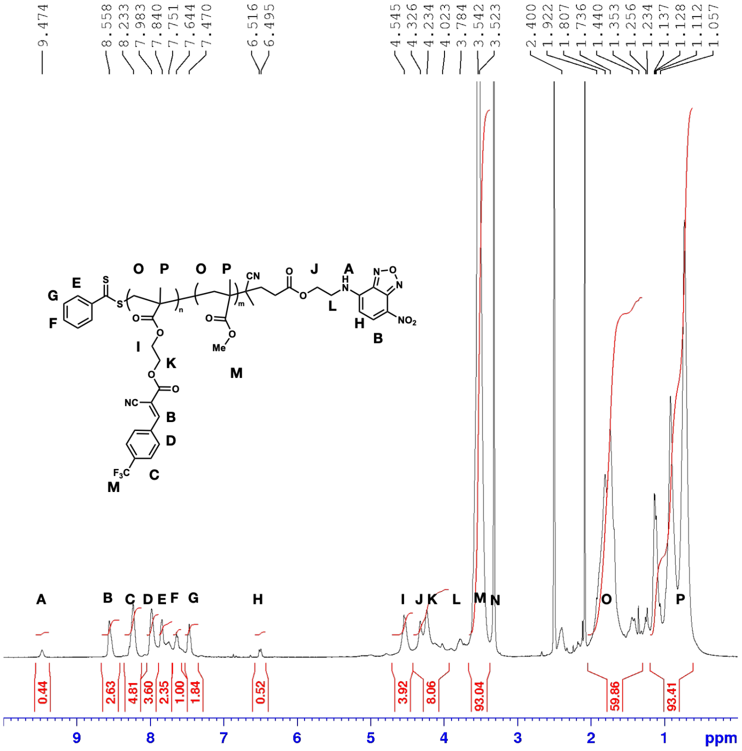

Figure S2.22 ^{1}H NMR spectrum of P1–NBD in DMSO at 25 °C.

P2–NBD. Following **General Procedure 5**, P2 –NBD was isolated as an orange powder. ^{1}H NMR (400 MHz, DMSO) δ 9.47 (bs), 8.52 (bs), 8.33 (bs), 8.11 (bs), 7.84 (bs), 7.64 (app. t, J = 6.9 Hz), 7.47 (app. t, J = 6.9 Hz), 7.17 (bs), 6.49 (app. d, J = 8.2 Hz), 4.48 (bs), 4.33 (bs), 4.22 (bs), 3.90 (bs), 3.77 (bs), 3.54 (bs), 2.04–1.36 (bs), 1.08–0.61 (bs). $M_{n,calcd.}$ = 4,850 g/mol.

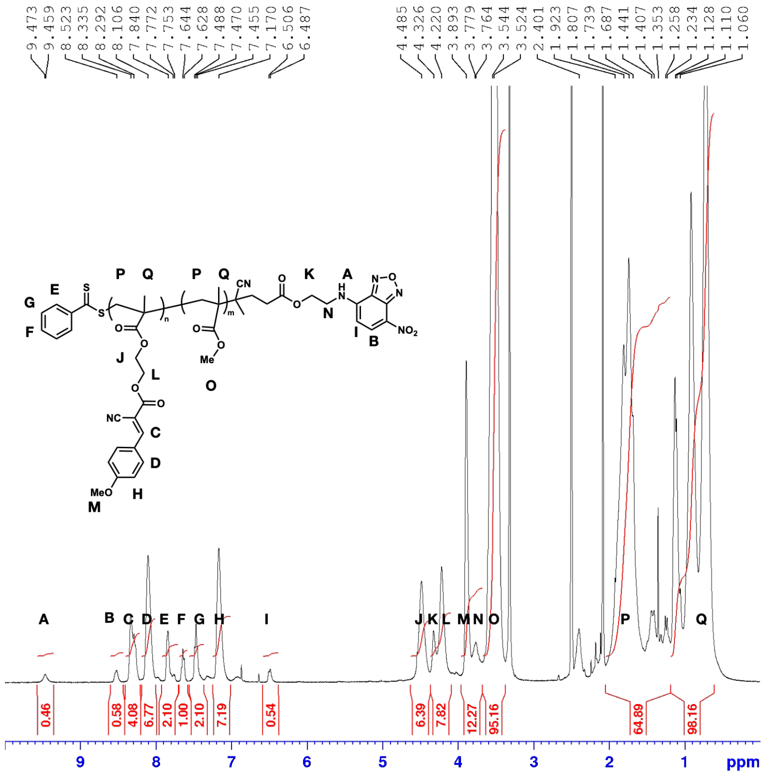

Figure S2.23 ^{1}H NMR spectrum of P2–NBD in DMSO at 25 °C.

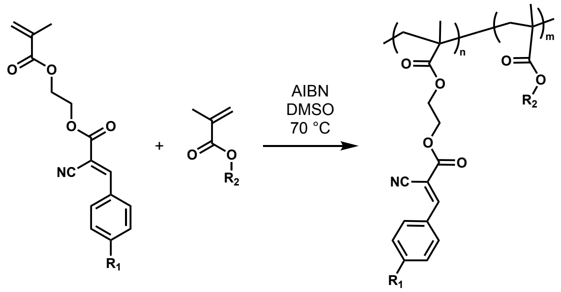

General Procedure 6 [P3 (B-CF_3-CH_3-10)]. BCAMA–CF_3 (707 mg, 2.00 mmol, 10 equiv) and MMA (1.93 mL, 18.00 mmol, 90 equiv) were dissolved in DMSO (9.37 mL) in a 20 mL vial. A 50 mg/mL solution of AIBN in DMSO (2.63 mL, 0.80 mmol, 4.0 equiv) was added and the vial was sealed using a rubber septum. Nitrogen gas was bubbled into the solution for 1 hour, and the reaction was heated to 70 °C for 1 day. The mixture was then poured into a 3.5 kDa MWCO dialysis tubing and dialyzed for 1 week against acetone, exchanging the solvent once a day. The acetone was evaporated under reduced pressure to afford P3 as a yellow powder. ^{1}H NMR (400 MHz, DMSO) δ 8.51 (bs), 8.21 (bs), 7.97 (bs), 4.79 (bs), 4.51 (bs), 4.22 (bs), 3.89 (bs), 3.57 (bs), 2.04–1.36 (bs), 1.08–0.61 (bs). $M_{n,\,GPC}$ = 18,056 g/mol, $M_w/M_{n,GPC}$ = 1.99.

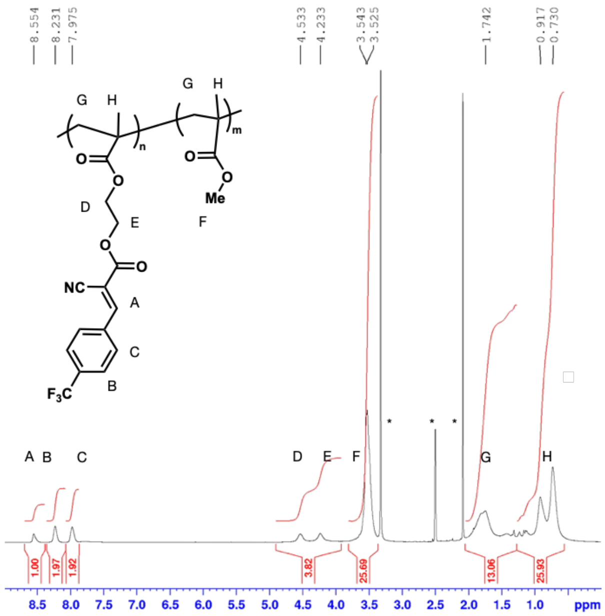

Figure S2.24 ^{1}H NMR spectrum of P3 in DMSO at 25 °C.

P4 (B-CF_3-$CH_2CH_2OCH_3$-10). Following **General Procedure 6**, P4 was isolated as a yellow powder. ^1H NMR (400 MHz, DMSO) δ 8.52 (bs), 8.22 (bs), 7.96 (bs), 4.52 (bs), 4.23 (bs), 4.02 (bs), 3.52 (bs), 3.27 (bs), 2.20–1.29 (bs), 1.29–0.62 (bs). $M_{n,\,GPC}$ = 18,065 g/mol, $M_w/M_{n,GPC}$ = 2.37.

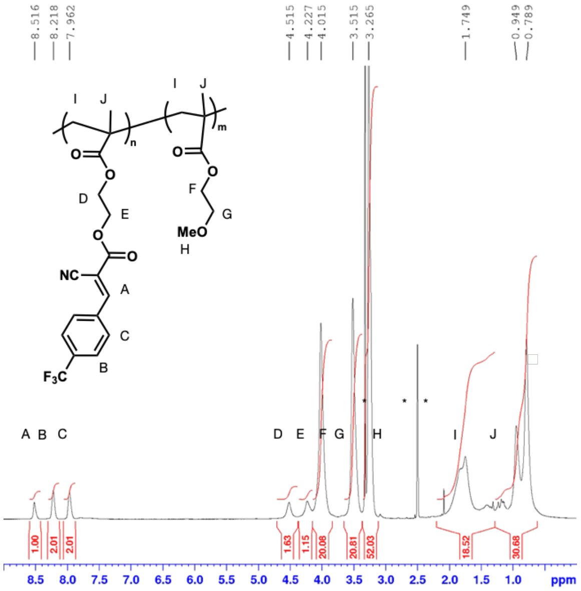

Figure S2.25 ^1H NMR spectrum of P4 in DMSO at 25 °C.

P5 (B-H-CH_3-10). Following **General Procedure 6**, P5 was isolated as a yellow powder. ^1H NMR (600 MHz, DMSO) δ 8.44 (bs), 8.09 (bs), 7.67 (bs), 7.62 (bs), 4.52 (bs), 4.23 (bs), 3.54 (app. bs), 2.03–1.25 (bs), 1.25–0.52 (bs). $M_{n,\,GPC}$ = 20,307 g/mol, $M_w/M_{n,GPC}$ = 1.89.

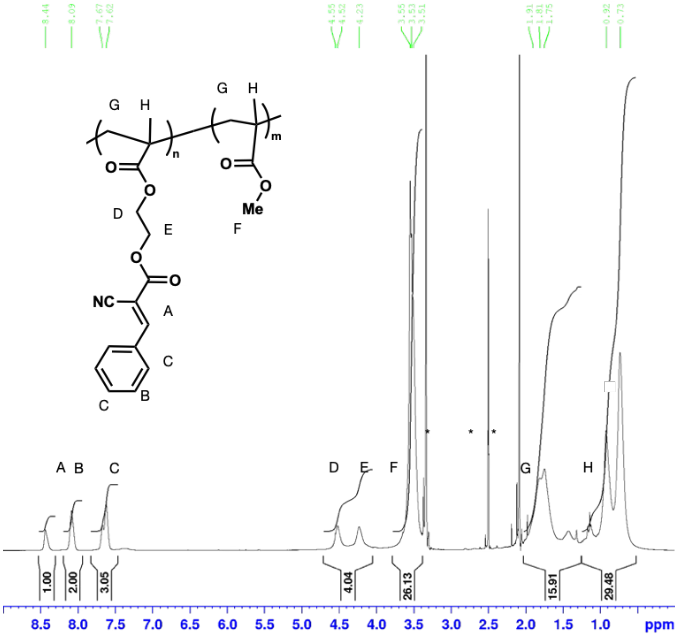

Figure S2.26 ^1H NMR spectrum of P5 in DMSO at 25 °C.

P6 (B-OMe-CH_3-10). Following **General Procedure 6**, P6 was isolated as a yellow powder. ^1H NMR (400 MHz, DMSO) δ 8.33 (bs), 8.11 (bs), 7.17 (bs), 4.48 (bs), 4.22 (bs), 3.90 (bs), 3.54 (app. bs), 2.04–1.22 (bs), 1.06–0.60 (bs). $M_{n,\,GPC}$ = 21,147 g/mol, $M_w/M_{n,GPC}$ = 1.98.

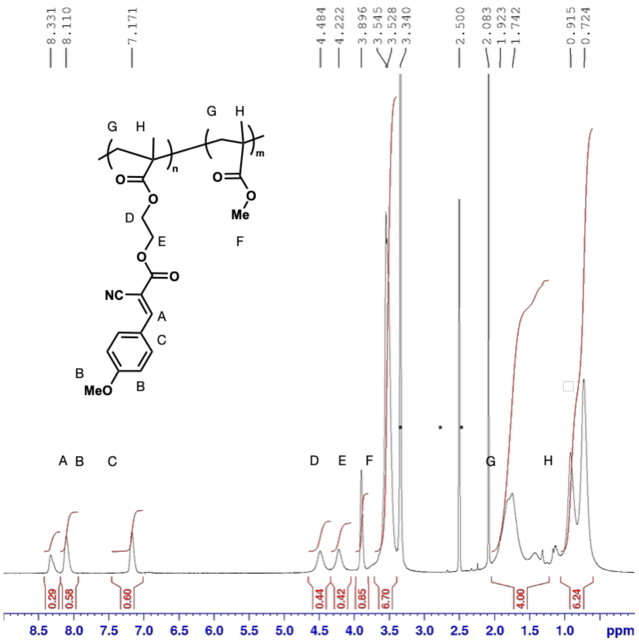

Figure S2.27 ^1H NMR spectrum of P6 in DMSO at 25 °C.

P7 (B-H-$CH_2CH_2OCH_3$-10). Following **General Procedure 6**, P7 was isolated as a yellow powder. ^1H NMR (600 MHz, DMSO) δ 8.41 (bs), 8.07 (bs), 7.66 (bs), 7.61 (bs), 4.50 (bs), 4.22 (bs), 4.02 (bs), 3.52 (bs), 3.27 (bs), 2.06–1.26 (bs), 1.24–0.50 (bs). $M_{n,\,GPC}$ = 21,795 g/mol, $M_w/M_{n,GPC}$ = 2.52.

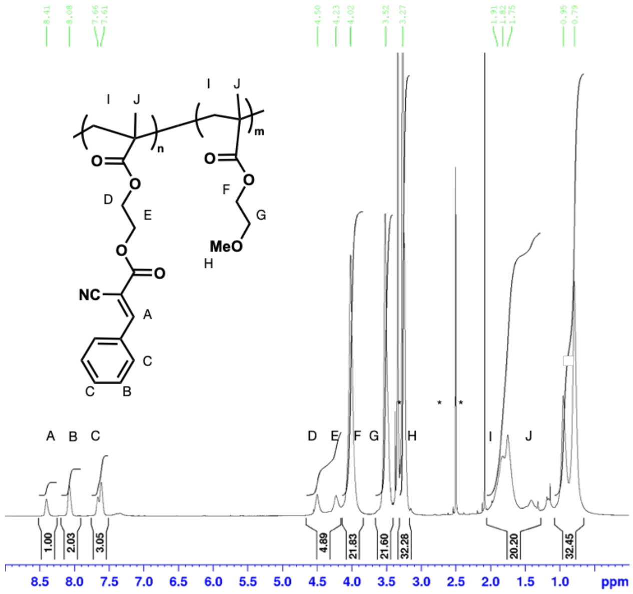

Figure S2.28 ^1H NMR spectrum of P7 in DMSO at 25 °C.

P8 (B-OMe-$CH_2CH_2OCH_3$-10). Following **General Procedure 6**, P8 was isolated as a yellow powder. ^1H NMR (400 MHz, DMSO) δ 8.30 (bs), 8.09 (bs), 7.16 (bs), 4.46 (bs), 4.22 (bs), 4.02 (bs), 3.89 (bs), 3.51 (bs), 3.26 (bs), 2.28–1.28 (bs), 1.22–0.63 (bs). $M_{n,\,GPC}$ = 24,809 g/mol, $M_w/M_{n,GPC}$ = 2.61.

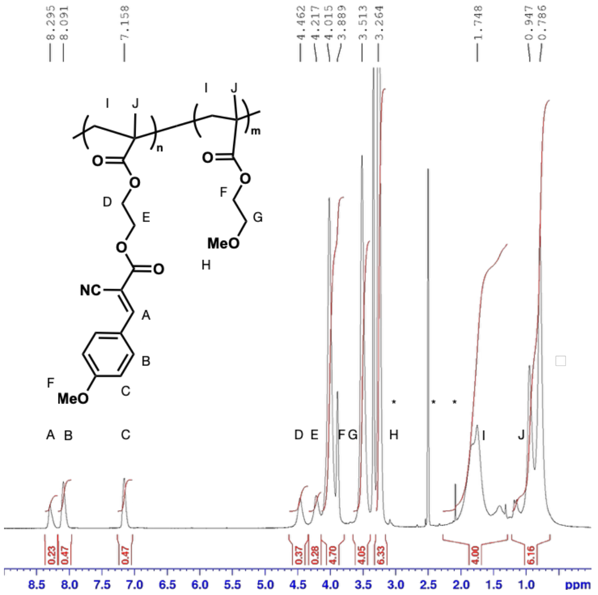

Figure S2.29 ^1H NMR spectrum of P8 in DMSO at 25 °C.

P9 (B-$CH_2CH_2OCH_3$-0). Following **General Procedure 6**, P9 was isolated as a white powder. ^1H NMR (400 MHz, DMSO) δ 4.03 (bs), 3.53 (bs), 3.27 (bs), 2.29–1.26 (bs), 1.24–0.49 (bs). $M_{n,\,GPC}$ = 34,259 g/mol, $M_w/M_{n,GPC}$ = 1.98.

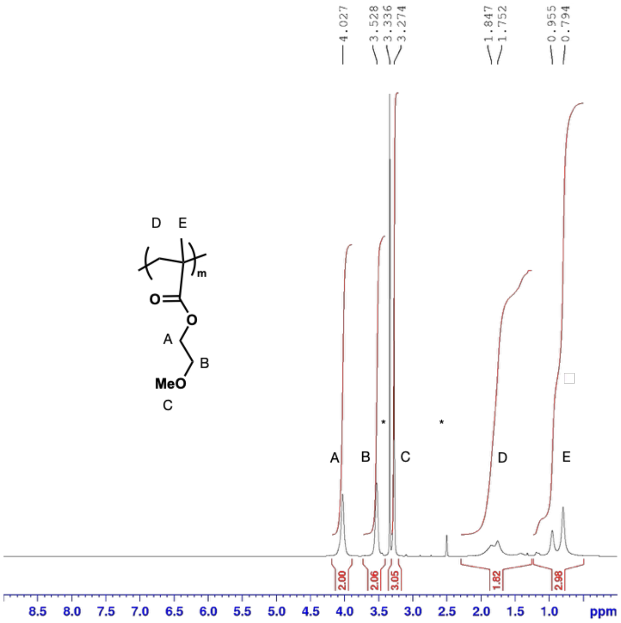

Figure S2.30 ^1H NMR spectrum of P9 in DMSO at 25 °C.

P10 (B-CF_3-$CH_2CH_2OCH_3$-5). Following **General Procedure 6**, P10 was isolated as a yellow powder. ^1H NMR (400 MHz, DMSO) δ 8.53 (bs), 8.23 (bs), 7.98 (bs), 4.52 (bs), 4.24 (bs), 4.02 (bs), 3.52 (bs), 3.27 (bs), 2.28–1.28 (bs), 1.22–0.63 (bs). $M_{n,\,GPC}$ = 19,266 g/mol, $M_w/M_{n,GPC}$ = 2.53.

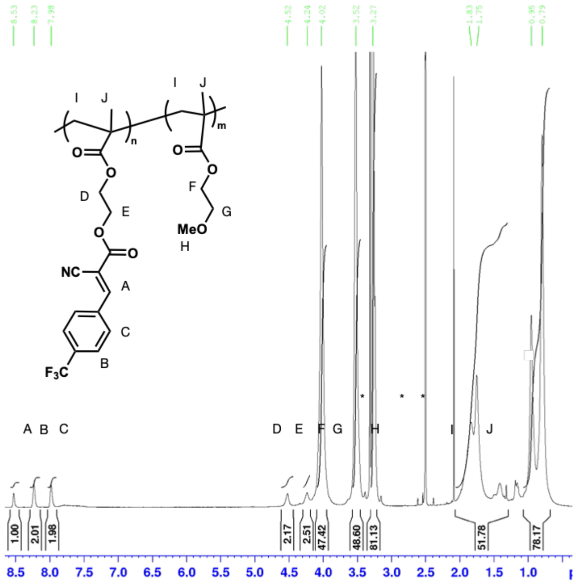

Figure S2.31 ^1H NMR spectrum of P10 in DMSO at 25 °C.

P11 (N-CF_3-$CH_2CH_2OCH_3$-10). BCAMA–CF_3 (707 mg, 2.00 mmol, 10 equiv) and EGMEMA (2.61 mL, 18.00 mmol, 90 equiv) were dissolved in DMSO (11.85 mL) in a 20 mL vial. A 50 mg/mL solution of AIBN in DMSO (0.02 mL, 0.006 mmol, 0.03 equiv) and a 100 mg/mL solution of 2-cyano-2-propyl benzodithioate (0.13 mL, 0.06 mmol, 0.3 quiv) were added and the vial was sealed using a rubber septum. Nitrogen gas was bubbled into the solution for 1 hour, and the reaction was heated to 70 °C for 3 days. The mixture was then poured into a 3.5 kDa MWCO dialysis tubing and dialyzed for 1 week against acetone, exchanging the solvent once a day. The acetone was evaporated under reduced pressure to afford P1 as a pink powder. ^1H NMR (400 MHz, DMSO) δ 8.53 (bs), 8.23 (bs), 7.97 (bs), 7.82 (bs), 7.63 (bs), 7.47 (bs), 4.52 (bs), 4.23 (bs), 4.02 (bs), 3.52 (bs), 3.27 (bs), 2.04–1.36 (bs), 1.08–0.61 (bs). $M_{n,\,GPC}$ = 25,825 g/mol, $M_w/M_{n,GPC}$ = 1.18.

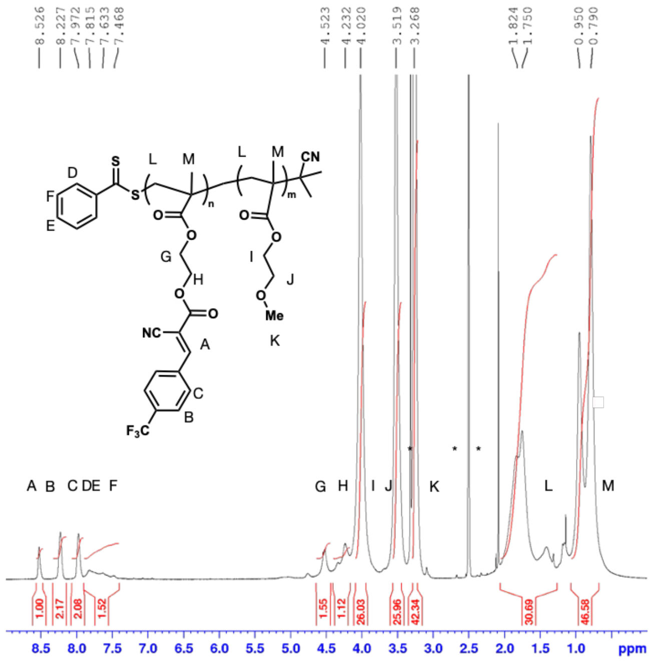

Figure S2.32 ^1H NMR spectrum of P11 in DMSO at 25 °C.

S3. Gel Permeation Chromatography (GPC) Measurements

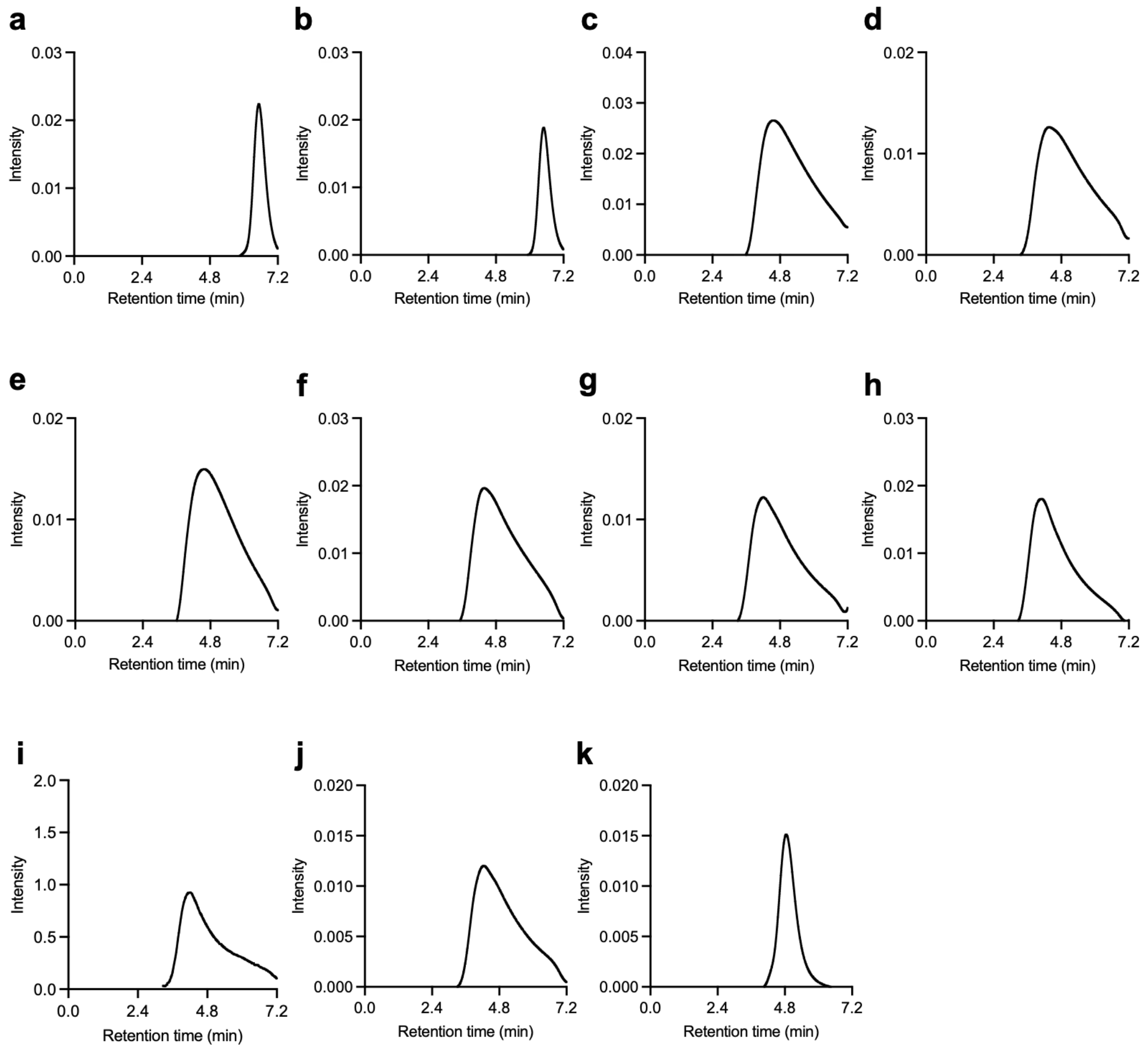

Figure S3.1. Gel permeation chromatography (GPC) traces plotted against retention time. (**a**) P1, (**b**) P2, (**c**) P3, (**d**) P4, (**e**) P5, (**f**) P6, (**g**) P7, h) P8, (**i**) P9, (**j**) P10, (**k**) P11.

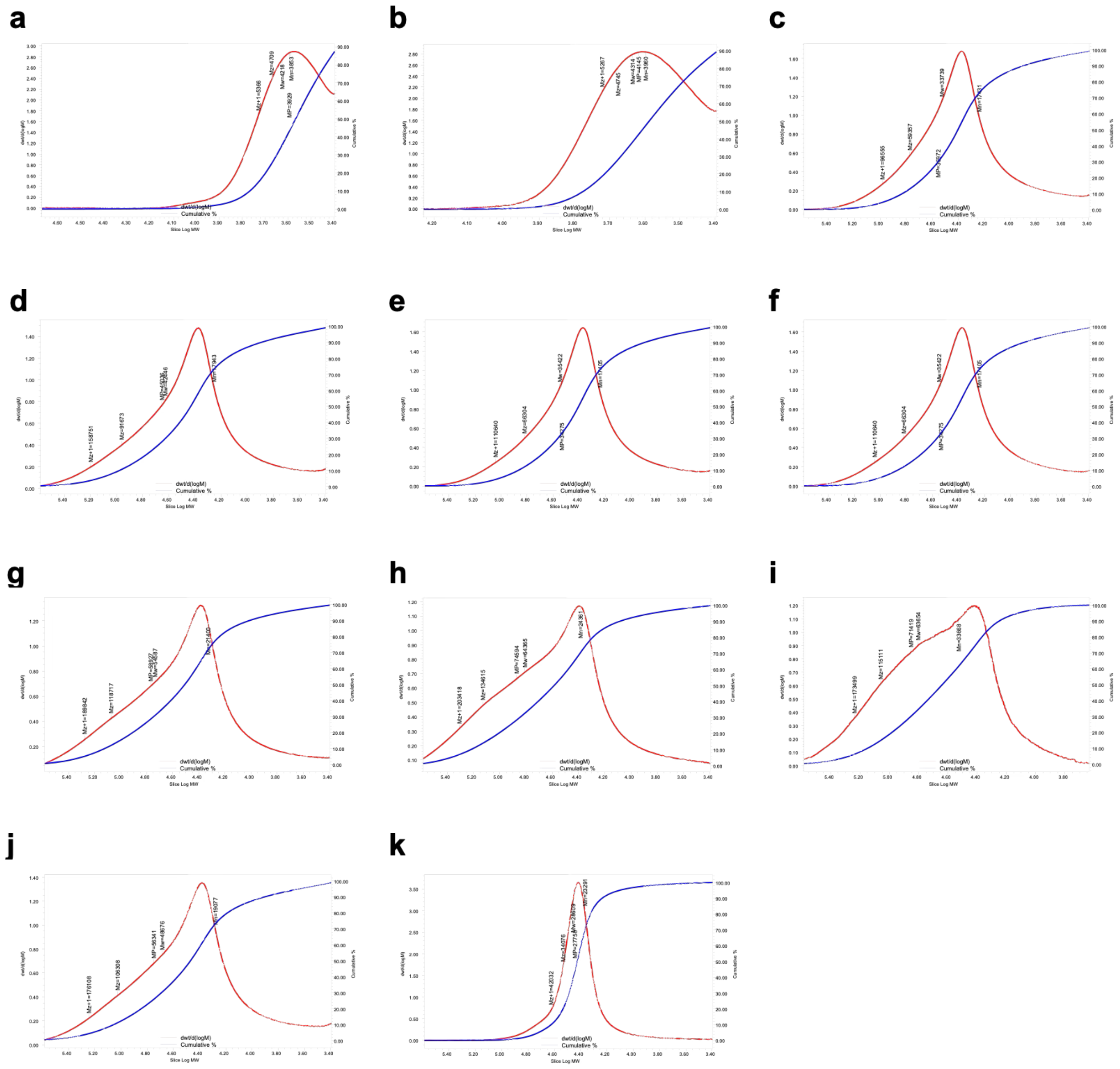

Figure S3.2. GPC trace converted to molecular weight distribution (Log MW). (**a**) P1, (**b**) P2, (**c**) P3, (**d**) P4, (**e**) P5, (**f**) P6, (**g**) P7, h) P8, (**i**) P9, (**j**) P10, (**k**) P11.

S4. BCA Reactivity in Organic Solvents (DMSO-d_6 and Acetone-d_6)

S4.1–S4.2. Aryl substituent-dependent conversion, equilibrium distribution, and rate

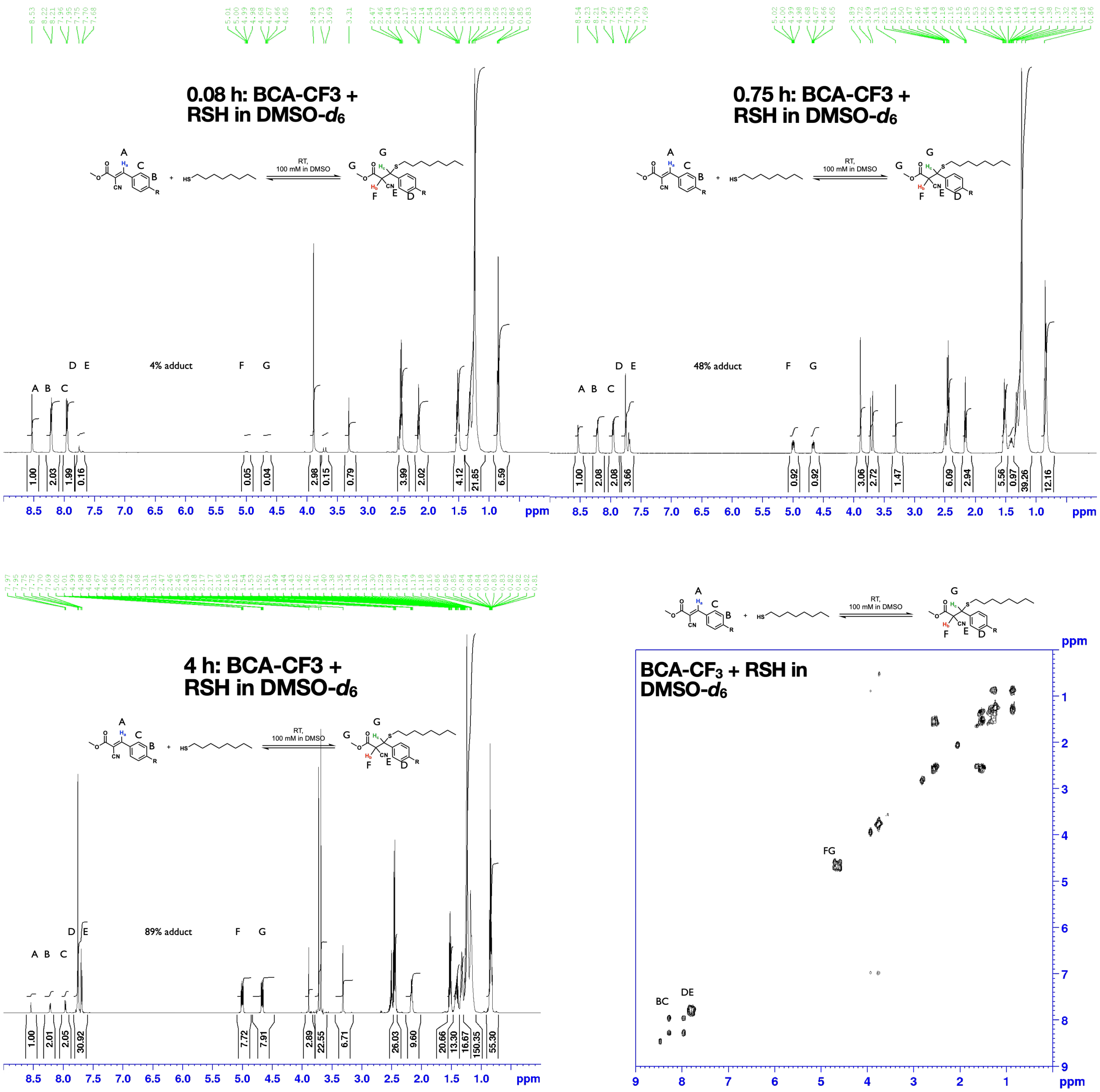

S4.1. TM adduct formation with BCA. An example of ^{1}H NMR analysis to determine % adduct formation over time based on relative integration values of key BCA and tM adduct peaks (e.g., at 4 h, 100% × ($X_{adduct}/X_{BCA+adduct}$) = 100% × [(7.72+7.91)/2]/ [(7.72+7.91)/2+1] = 89%. ^{1}H–^{1}H COSY confirms the assignment of protons belonging to BCA (BC) and the adduct (DE, FG).

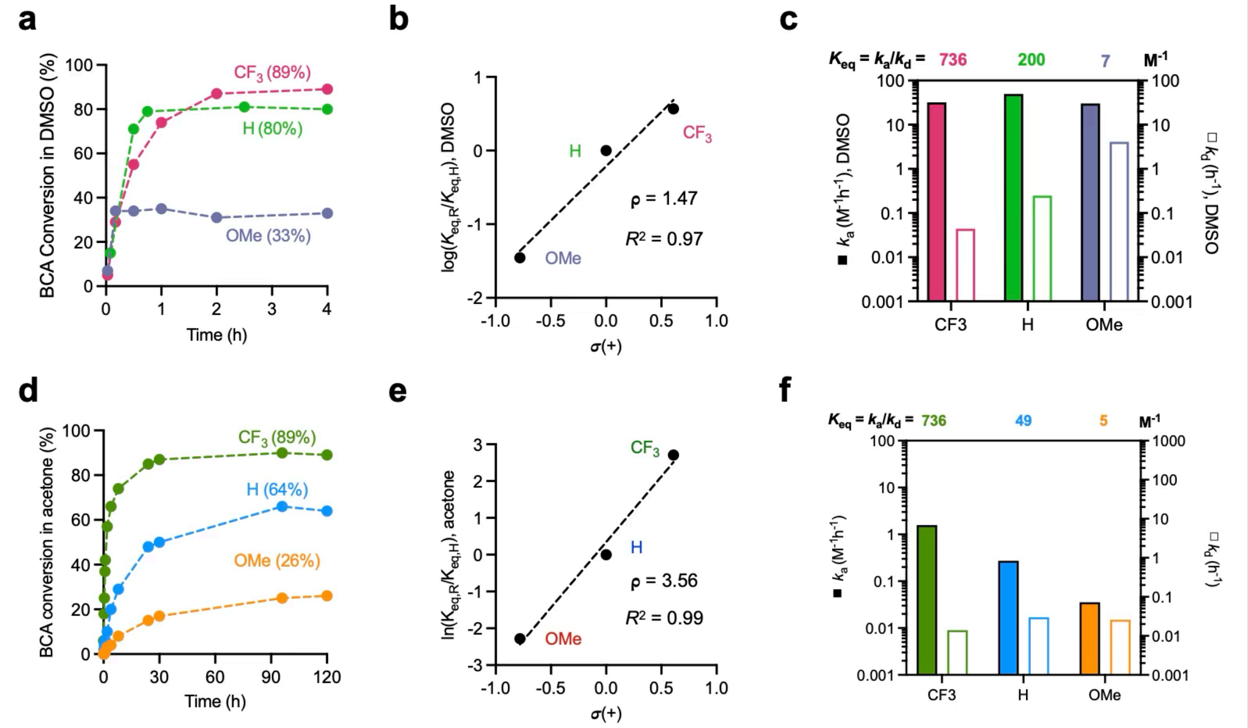

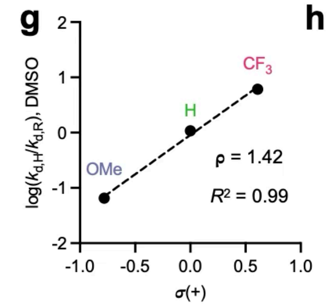

h

$R_{solvent}$	k_a ($M^{-1}h^{-1}$)	$t_{1/2}$ (h)	k_d (h^{-1})	$t_{1/2}$ (h)	K_{eq} (M^{-1})	[pdt], M	[BCA], M	$X_{pdt, eqm}$	$[BCA]_0$, M	Slope, 1/(1-X) vs. t
$CF_{3, DMSO}$	32.03	0.31	0.044	15.917	736	0.089	0.011	0.89	0.1	3.203
$H_{, DMSO}$	49.57	0.20	0.248	2.797	200	0.08	0.02	0.80	0.1	4.957
$OMe_{, DMSO}$	30.15	0.33	4.101	0.169	7	0.033	0.067	0.33	0.1	3.015
$CF_{3, Acetone}$	6.874	1.45	0.009	74.169	736	0.089	0.011	0.89	0.1	0.6874
$H_{, Acetone}$	0.8388	11.92	0.017	40.808	49	0.064	0.036	0.64	0.1	0.08388
$OMe_{, Acetone}$	0.07249	137.95	0.015	45.400	5	0.026	0.074	0.26	0.1	0.007249

i

K_{eq} calculation: $K_{eq} = X_{eq}/[BCA]_0(1-X_{eq})^2$

1) $[BCA]_0 = 0.1$ M; $[tM]_{eq} = [BCA]_0X_{eq}$ & $[BCA]_{eq} = [BCA]_0(1-X_{eq})$; $[BCA]_{eq} = [RSH]_{eq}$

2) $K_{eq} = [tM]_{eq}/[BCA]_{eq}^2 = [BCA]_0X_{eq}/([BCA]_0(1-X_{eq}))^2 = X_{eq}/[BCA]_0(1-X_{eq})^2$

k_a calculation: $k_a = slope_{linear}/[BCA]_0$

1) $X = [tM]/[BCA]_0$; $[BCA] = [BCA]_0(1-X)$; 2nd order rate law $1/[BCA] = 1/[BCA]_0 + k_a(t)$ (Assume reverse reaction is negligible early on)

2) $1/[BCA]_0(1-X)=1/[BCA]_0+k_a(t)$; $1/(1-X)=1+[BCA]_0k_a(t)$
Plot 1/(1-X) vs. t for linear region
y-intercept = 1; Slope = $[BCA]_0k_a$; k_a = slope/$[BCA]_0$

k_d calculation: $k_d = k_a/K_{eq}$

1) $K_{eq} = k_a/k_d$

2) $k_d = k_a/K_{eq}$

S4.2. Kinetic study and estimation of K_{eq}, k_a, and k_d. BCA (100 mM) conversion over time with equimolar amounts of 1-octanethiol in (**a**) DMSO and (**d**) acetone as determined by ^{1}H NMR. Linear free energy plots correlating Hammett constant σ^+ with equilibrium constants in (**b**) DMSO and (**e**) acetone and (**g**) dissociation rate constants in DMSO. Calculated K_{eq}, k_a, and k_d values of the different BCA derivatives reacting with thiols in (**c**) DMSO and (**f**) acetone. (**h**) Table of K_{eq}, k_a, k_d, and other values used for calculation purposes. (**i**) Calculation of K_{eq}, k_a, and k_d using NMR integration, K_{eq} formula, 2nd order (bimolecular reaction) rate law, and fractional conversion relationships.

S4.3. TM bond exchange and dissociation

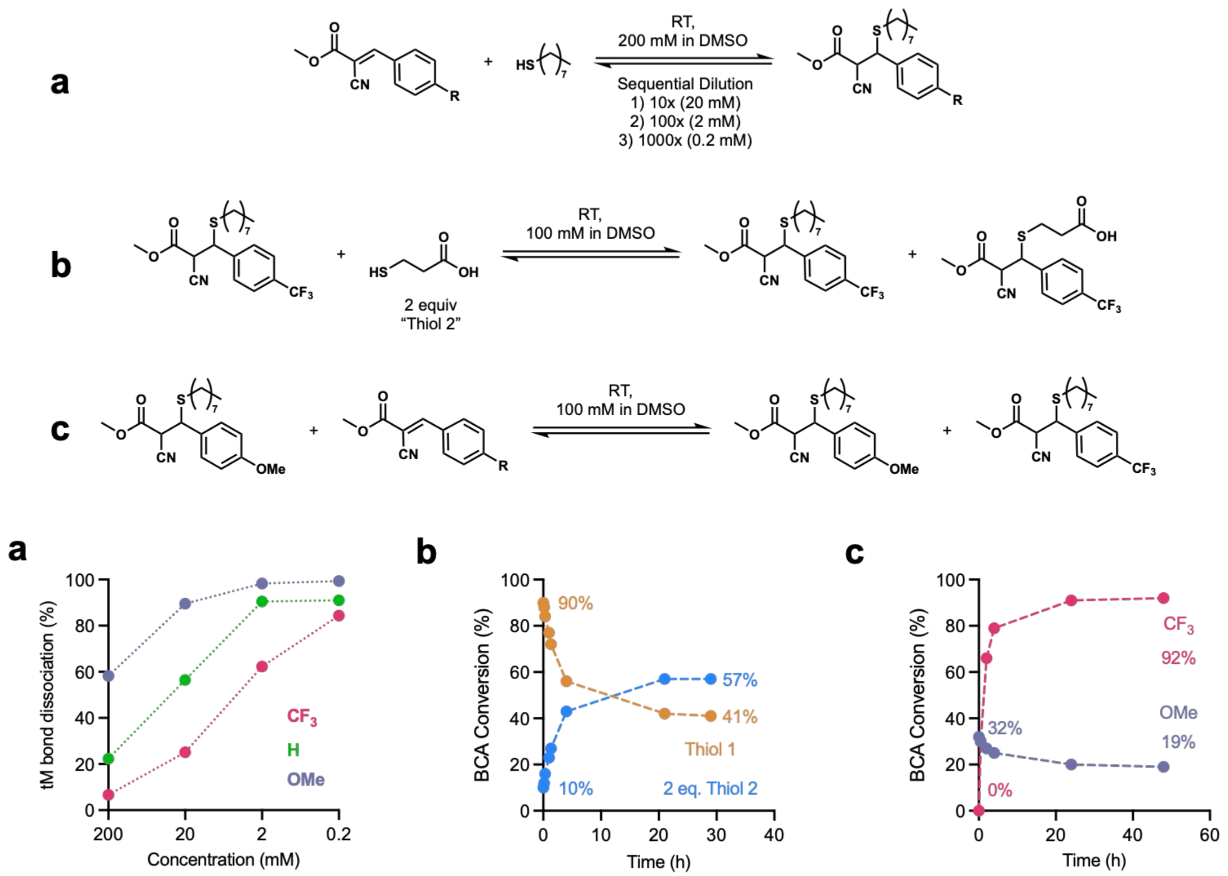

S4.3. TM equilibrium perturbations upon dilution and introduction of exogeneous thiols or BCAs. (**a**) TM adduct dissociation of various BCA derivatives (R = CF_3, H, or OMe) at 10x, 100x, and 1000x dilution starting from 200 mM each of the BCA and 1-octanethiol. (**b**) TM bond exchange (R = CF_3) over time upon the addition of 2 equivalents of a second, distinct thiol species (3-mercaptopropionic acid). (**c**) TM bond exchange of a OMe-based adduct over time upon the addition of a second, more reactive BCA (R = CF_3).

5. Spore Labeling

5.1. Detection of spore surface thiols

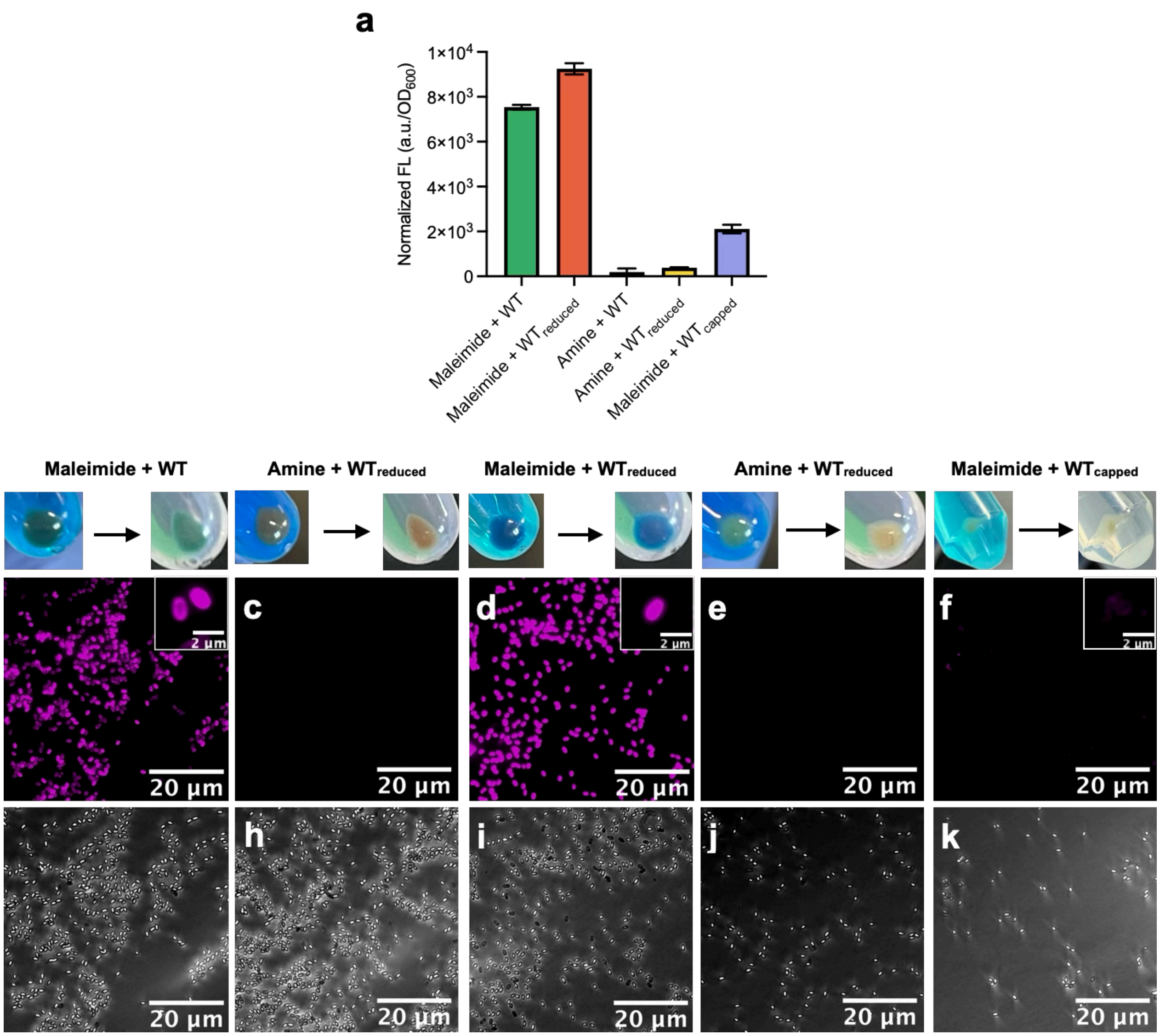

S5.1. Covalent labeling of wild-type *Bacillus subtilis* spores in DMSO. (**a**) Fluorescence intensity of each sample divided by their respective spore concentrations (OD_{600}). (**a–f**) Fluorescence (λ_{ex} = 630 nm, λ_{em} = 690–740 nm) and (**g–l**) bright-field microscope images of *B. subtilis* spore suspensions after fluorescent probes and spores were incubated in DMSO and washed with DMSO for 6 times.

S5.2. Fluorescent probes

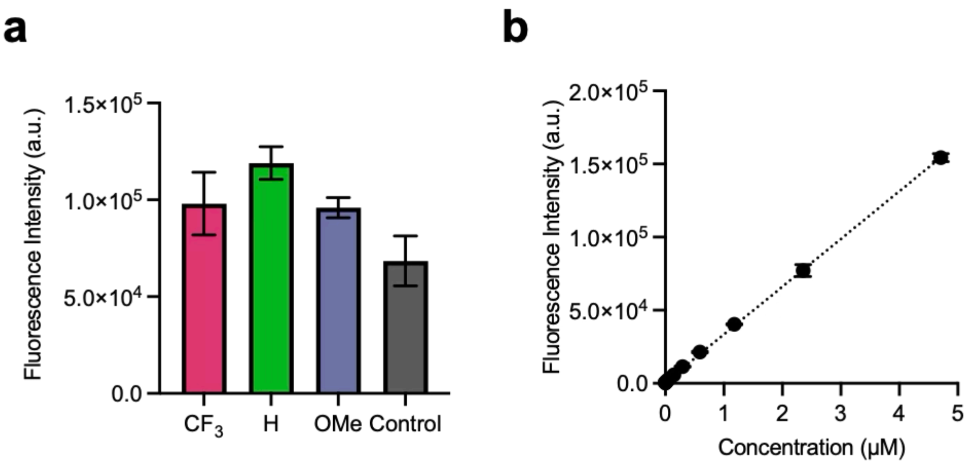

Fluorescence Intensity (a.u.) = 32632*[dye concentration (μM)] + 817.6; R^2 = 0.9979

c

R group	CF_3	H	OMe	Control
Average F.I. (a.u.)$_{100x}$	98026	118984	95971	68453
[NBD–BCA]$_{stock,\ 100x\ dilution}$ (μM)	3.0	3.6	2.9	2.1
[NBD–BCA]$_{stock}$ (μM)	299.2	363.5	292.9	208.6
$V_{NBD–BCA–R}$	3.3	2.8	3.4	4.8
V_{DMSO}	1.7	2.2	1.6	0.2
$V_{spores\ (5\ mg/mL\ in\ DMSO)}$	95.0	95.0	95.0	95.0

S5.2. Determination of stock solution concentration. (**a**) Triplicate fluorescence spectroscopy measurements of NBD–BCA–R stock solutions in DMSO. Control = NBD–OH. Error bars = standard error of the mean. (**b**) Standard curve of fluorescence intensity vs. dye concentration of NBD–OH and its corresponding linear regression equation. (**c**) A table summary of the volumes of stock solutions added to the reaction mixtures to create a final concentration of 10 μM, calculated from the stock solution concentrations.

S5.3. Spore labeling with fluorescent BCA probes

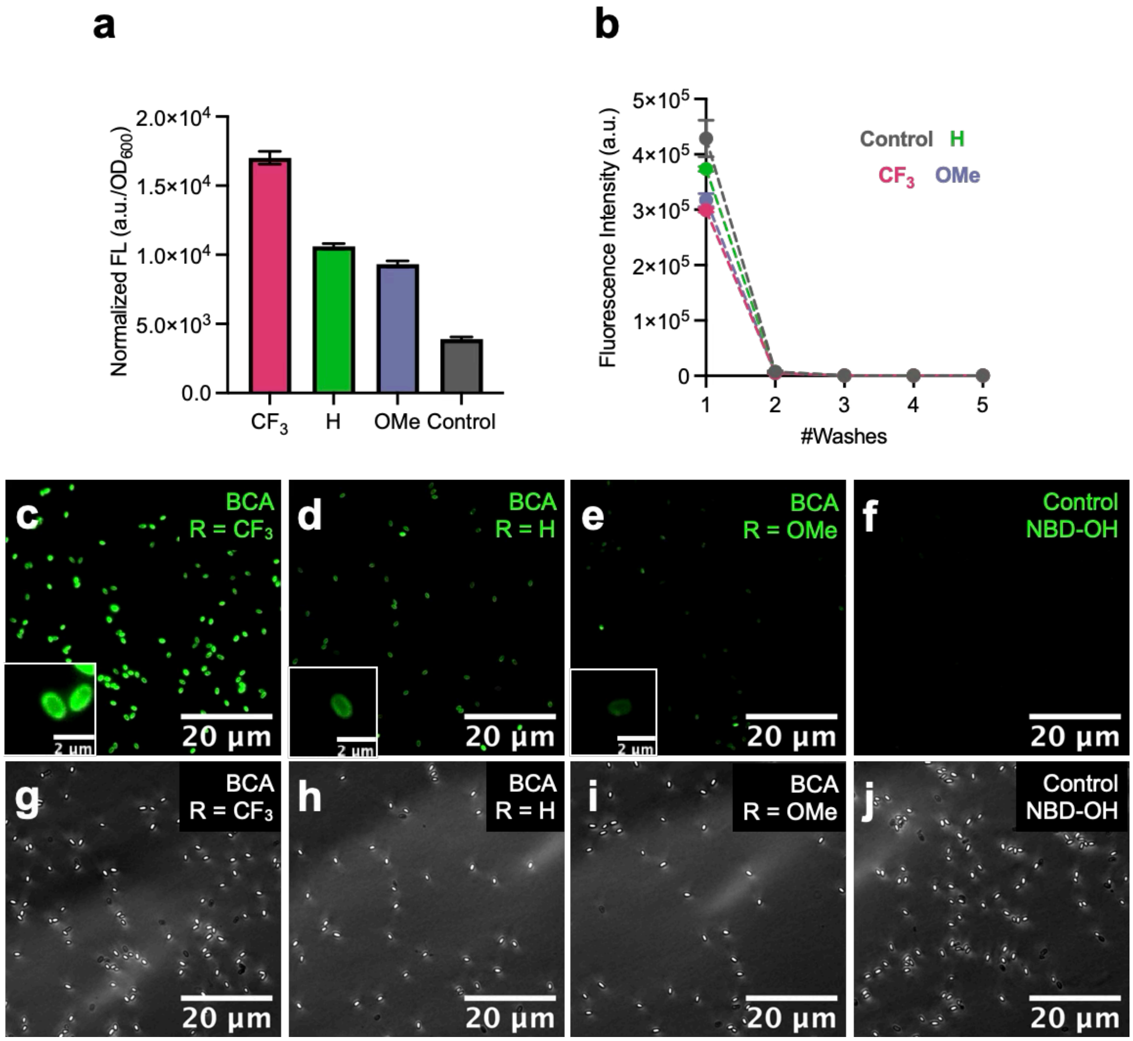

S5.3. Tunable tM bond formation on the *B. subtilis* spore surface. (**a**) Normalized fluorescence intensity (λ_{ex} = 460 nm, λ_{em} = 540 nm) of each sample (FL_{sample} − $FL_{supernatant}$)/($OD_{600,\ sample}$ − $OD_{600,\ supernatant}$). Control = NBD–OH. Error bars = standard error of the mean. (**b**) Fluorescence intensity measurements of the supernatant after each DMSO wash. $FL_{supernatant}$ ≈ 456 a.u. after 5 washes. (**c–f**) Fluorescence (λ_{ex} = 488 nm, λ_{em} = 510 nm) and (**g–j**) bright-field microscope images of *B. subtilis* spore suspensions in DMSO after the washes.

S5.4–S5.6. ^{1}H NMR analysis of covalently labeled spores

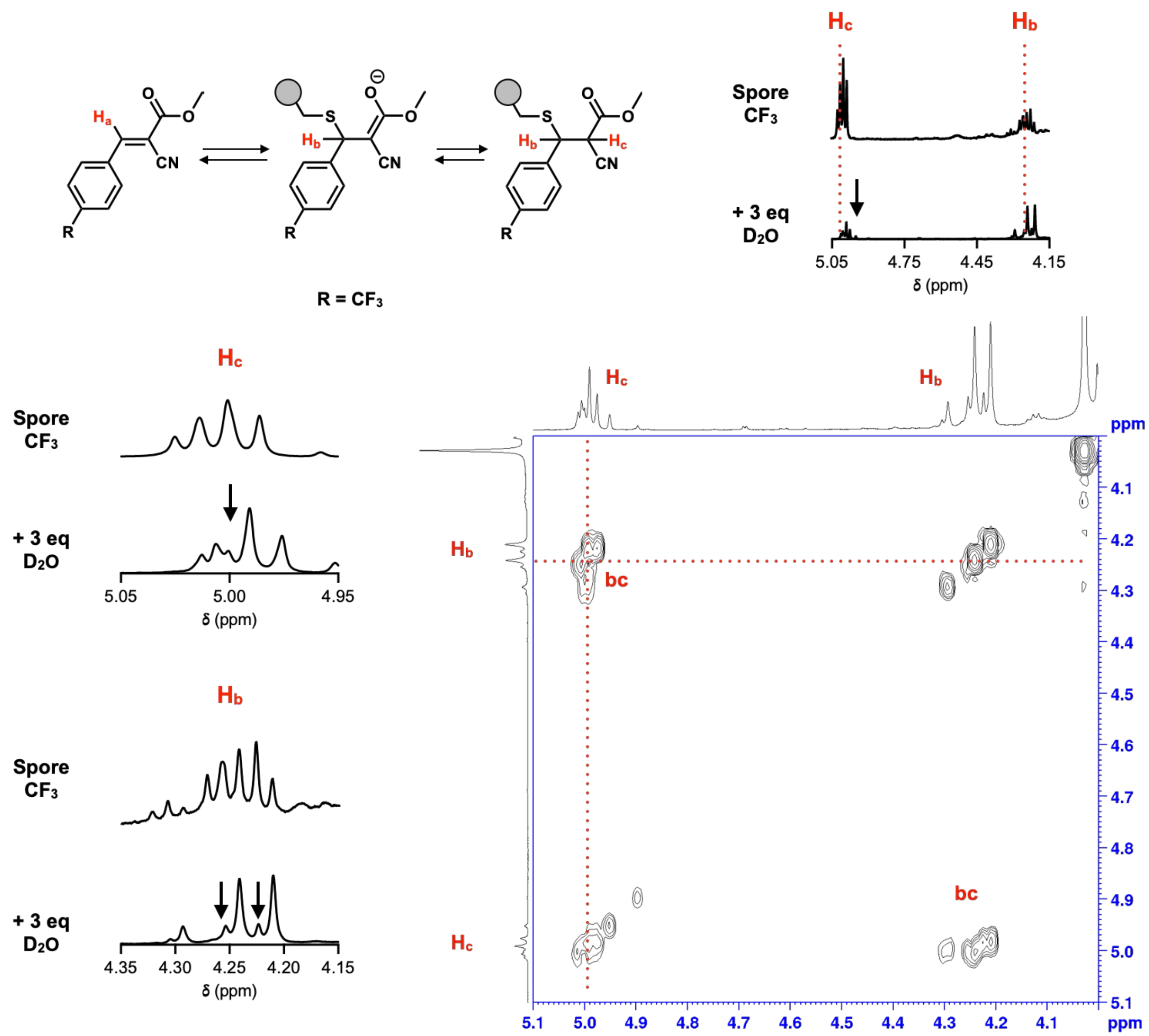

Figure S5.4. Confirmation of BCA–spore bond formation with ^{1}H and ^{1}H–^{1}H COSY NMR. Characteristic proton peaks of the tM adduct, H_c (**α**-proton) and H_b (**β**-proton/benzylic proton) observed from a DMSO-d_6 mixture of 200 mM BCA–CF_3 and 1 mg/mL spores. H_c appears at a chemical shift identical to that of the small molecule model (1-octanethiol instead of spores) spectrum, while the COSY cross peak (bc) reveals the upfield shifted H_b peak.

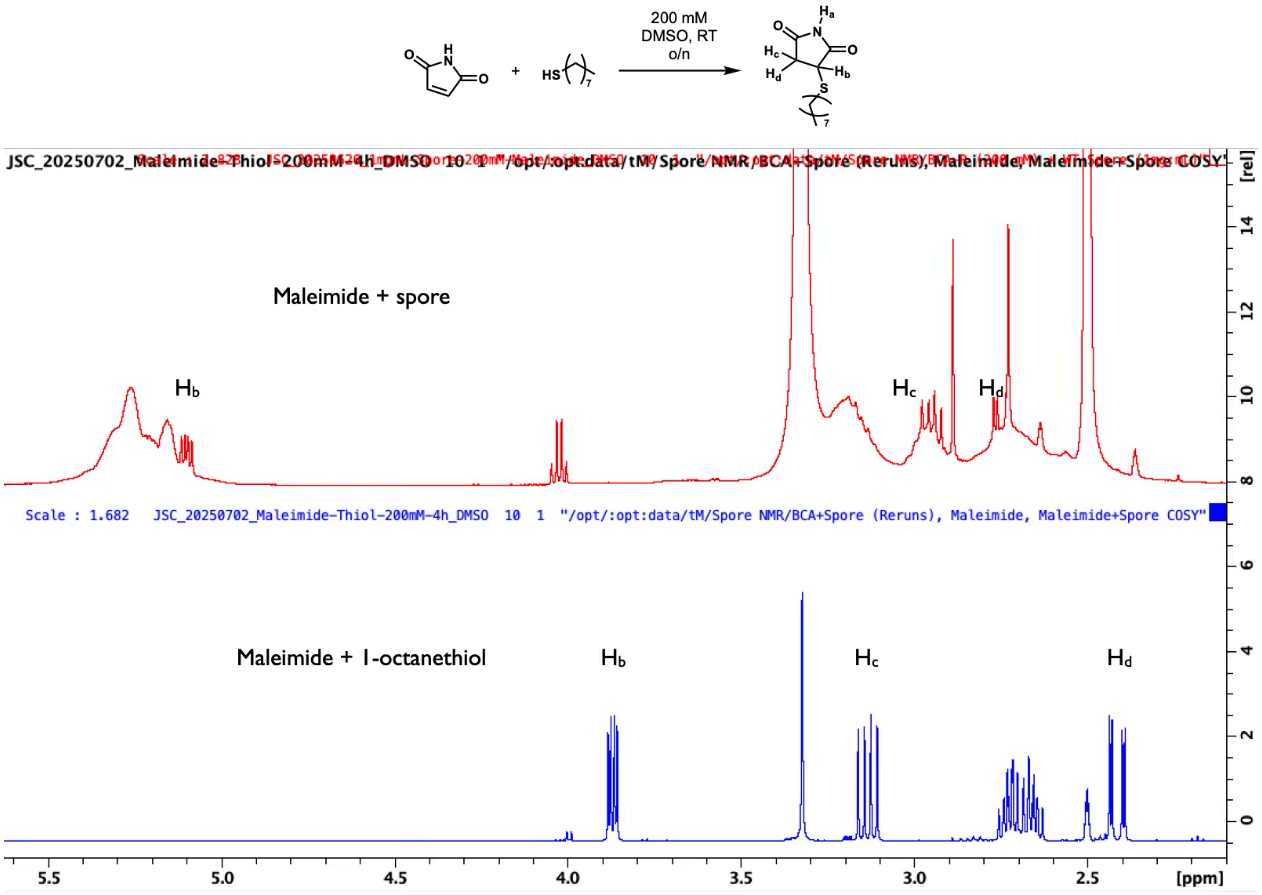

Figure S5.5. ^{1}H NMR of maleimide–spore bonds. Characteristic proton peaks H_b, H_c, and H_d of the tM adducts formed between maleimide and spores (top) and 1-octanethiol (bottom). Similar splitting patterns and relative integration values were observed, but with notable differences in chemical shifts.

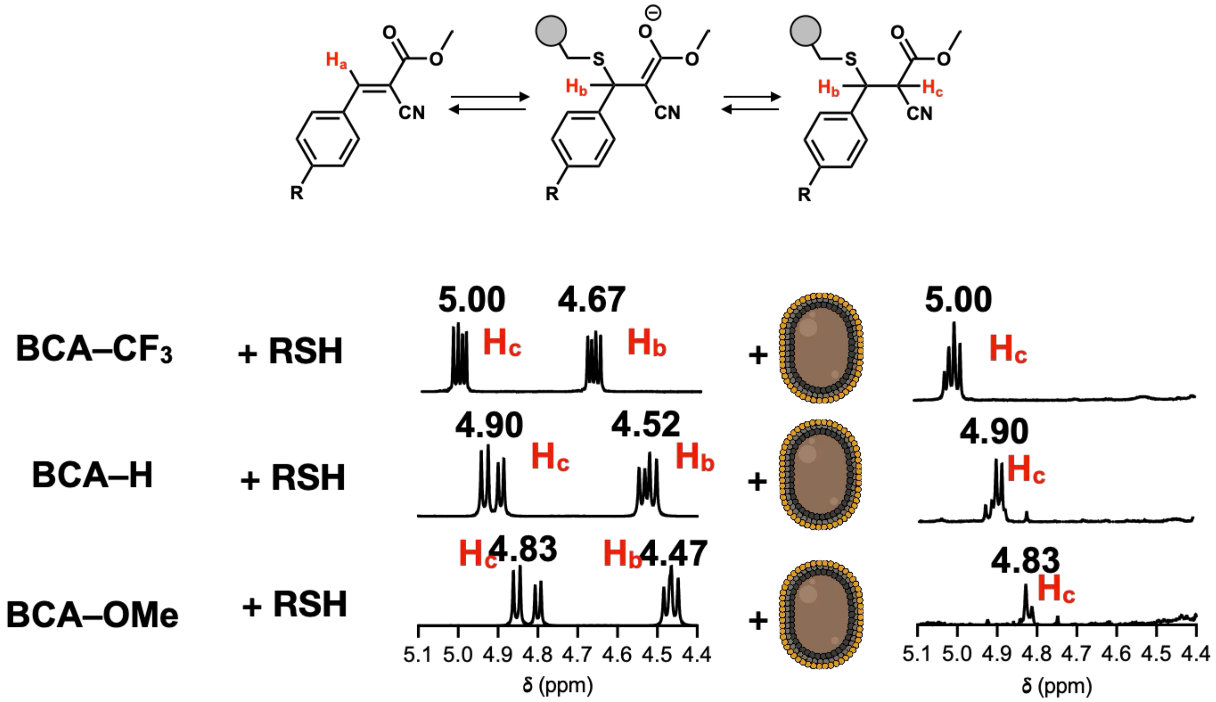

Figure S5.6. Comparison of key tM adducts peaks with 1-octanethiol and with spores. The characteristic ^{1}H NMR signals H_c of the tM adduct appear at identical chemical shifts when BCA–R were reacted with 1-octanethiol and spores. At the same concentration of the BCA (200 mM) and spores (1 mg/mL), the relative intensity of the H_c peaks reflect the expected differences in equilibrium distribution between R = CF_3, H, and OMe. H_b peaks were consistently confirmed to be shifted upfield using COSY NMR analysis (as seen in Figure S5.4).

S6. Fluorescent Polymer–Spore Assemblies

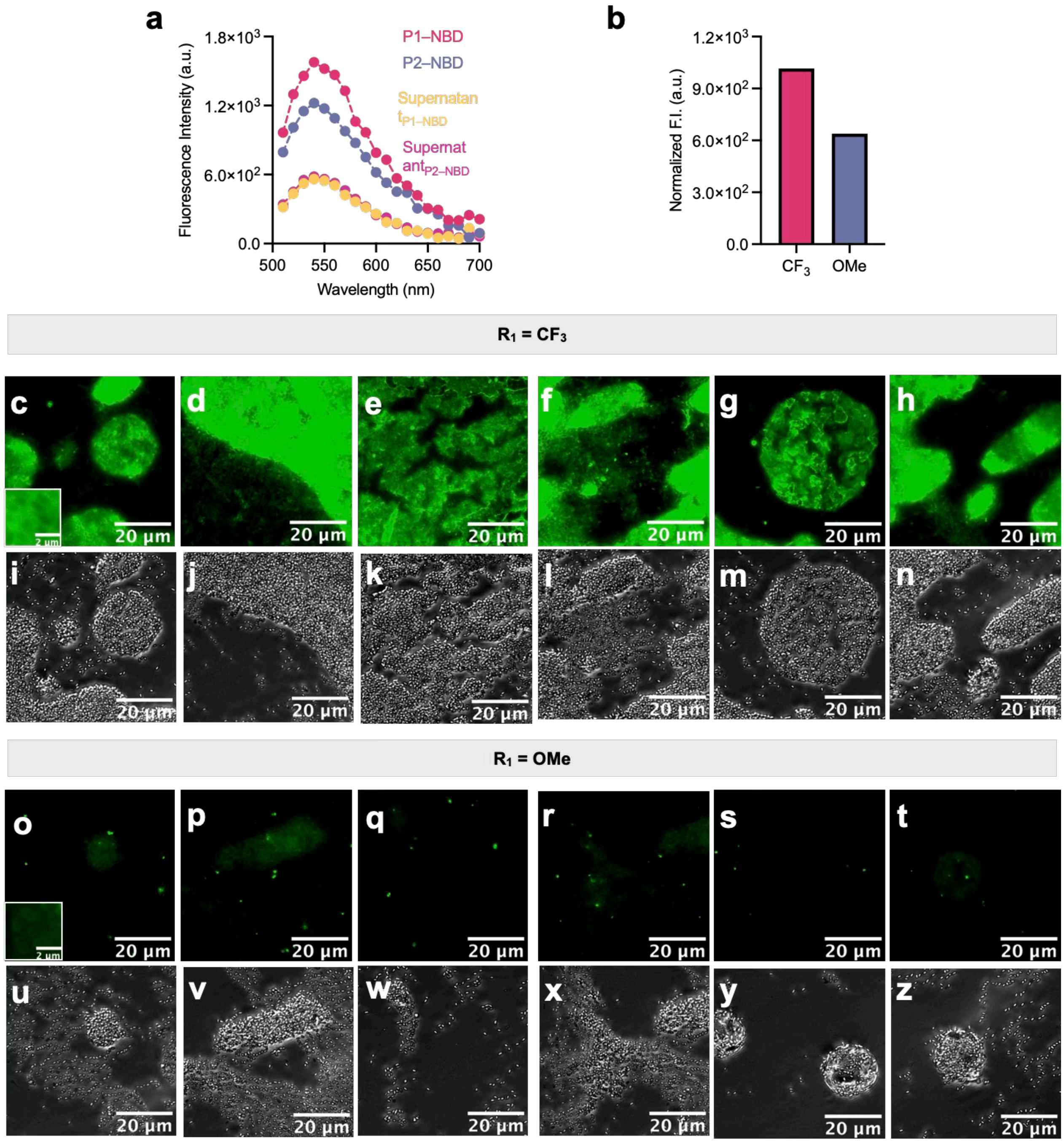

Figure S6. **Fluorescent polymer–spore assemblies in DMSO.** (**a**) Fluorescence emission spectrum (λ_{ex} = 460 nm, λ_{em} = 540 nm) of each sample and their supernatant. (**b**) Normalized fluorescence intensity ($FL_{sample} - FL_{supernatant}$) of the polymer–spore assemblies. Fluorescence (λ_{ex} = 488 nm, λ_{em} = 510 nm) micrographs of the polymer and spore mixtures using (**c**–**h**) P1–NBD and (**o**–**t**) P1–NBD, and their corresponding bright-field images (**i**–**n**) and (**u**–**z**).

S7. Approximation of Relative DMSO Affinity Using Hansen Solubility Parameters

The Hansen solubility parameter distance (R_a) between the solvent (DMSO) and small molecule analogues of the variable side chain R_2 of each polymer was calculated using estimated Hansen solubility parameters[4] with the definitions, values, and relationships clarified below:

$\boldsymbol{\delta}_d$ = dispersion forces; $\boldsymbol{\delta}_p$ = polar (dipole–dipole) interactions; $\boldsymbol{\delta}_h$ = hydrogen bonding capacity

DMSO: $\boldsymbol{\delta}_d = 18.4$, $\boldsymbol{\delta}_p = 16.4$, $\boldsymbol{\delta}_h = 10.2$

n-Pentane (similar to –CH_3 of P3): $\boldsymbol{\delta}_d = 15.6$; $\boldsymbol{\delta}_p = 0.0$; $\boldsymbol{\delta}_h = 0.0$

Ethylene glycol monoethyl ether (similar to –$CH_2CH_2OCH_3$ of P4): $\boldsymbol{\delta}_d = 16.2$; $\boldsymbol{\delta}_p = 9.2$; $\boldsymbol{\delta}_h = 14.3$

$R_a^2 = 4(\boldsymbol{\delta}_{d1} - \boldsymbol{\delta}_{d2})^2 + (\boldsymbol{\delta}_{p1} - \boldsymbol{\delta}_{p2})^2 + (\boldsymbol{\delta}_{d1} - \boldsymbol{\delta}_{h2})^2$

	DMSO	P3 (*n*-Pentane)	P4 (Ethylene glycol monoethyl ether)
$\boldsymbol{\delta}_d$	18.4	15.6	16.2
$\boldsymbol{\delta}_p$	16.4	0.0	9.2
$\boldsymbol{\delta}_h$	10.2	0.0	14.3
$R_{a,\ \text{DMSO–side chain}}$	0.0	6.1	3.4

S8. Solvent Expulsion and Evaporation from Material

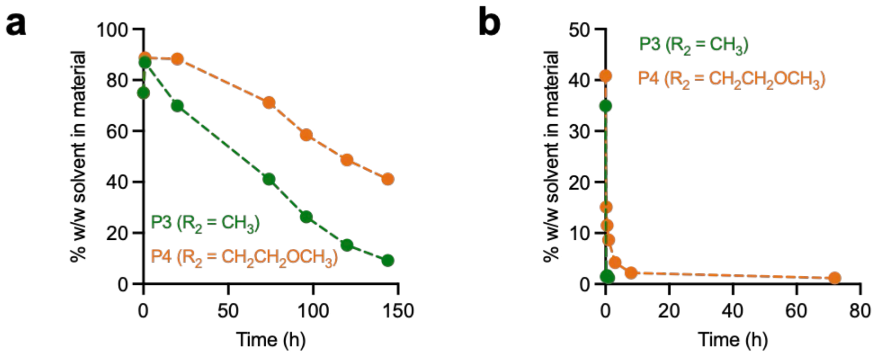

Figure S8. Loss of solvent over time. (**a**) % Residual solvent in DMSO organogels tracked by mass over time. (**b**) % Residual solvent of materials that were submerged in ethanol for 2 hours for a solvent exchange. The volatility of ethanol enables efficient solvent evaporation.

S9. Rheological Characterization and Analysis of Polymer–Spore Organogels

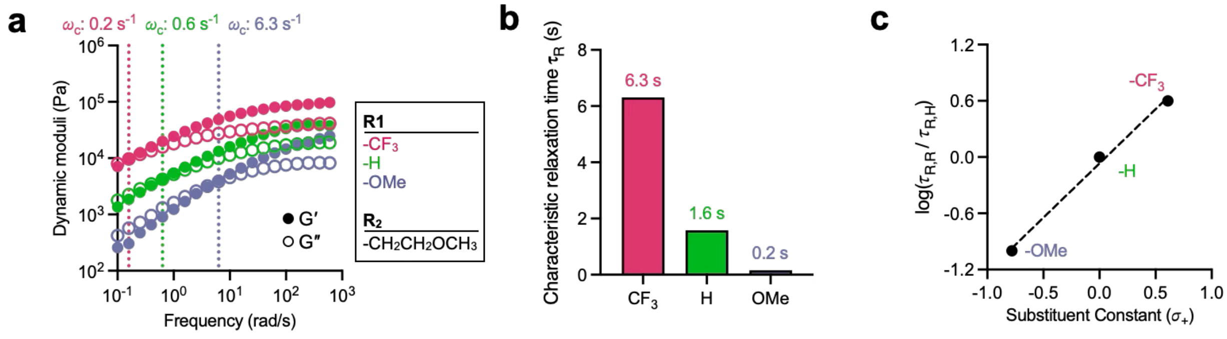

Figure S9. Programmable viscoelastic behavior of EGMEMA-based materials. (**a**) Frequency sweep rheology measurements of EGMEMA-based organogels. (**b**) Characteristic relaxation time τ_R of the EGMEMA-based materials derived from the crossover frequency ($\tau_R = 1/\omega_c$). (**c**) Hammet plot correlating the relaxation time of the material to the expected effect of the aryl substituent (σ^+).

S10. Preparation and Characterization of Biocomposites

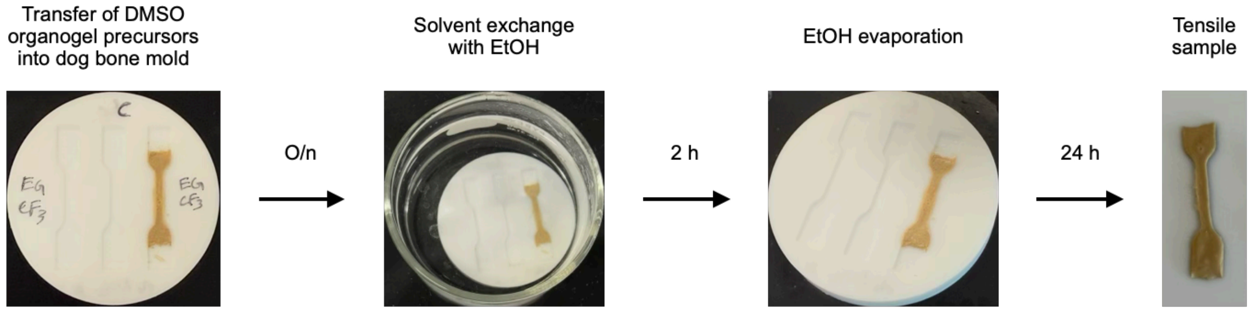

Figure S10.1. Sample preparation for tensile experiments.

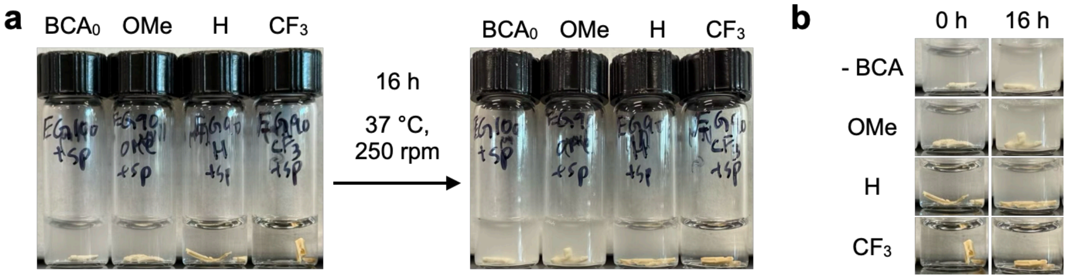

Figure S10.2. R_1-dependent covalent biocontainment in dry biocomposite materials. (**a**) Images of the experimental setup showing capped vials containing various biocomposite materials submerged in PBS (pH = 7.12). (**b**) Side-by-side comparison of solution turbidity.

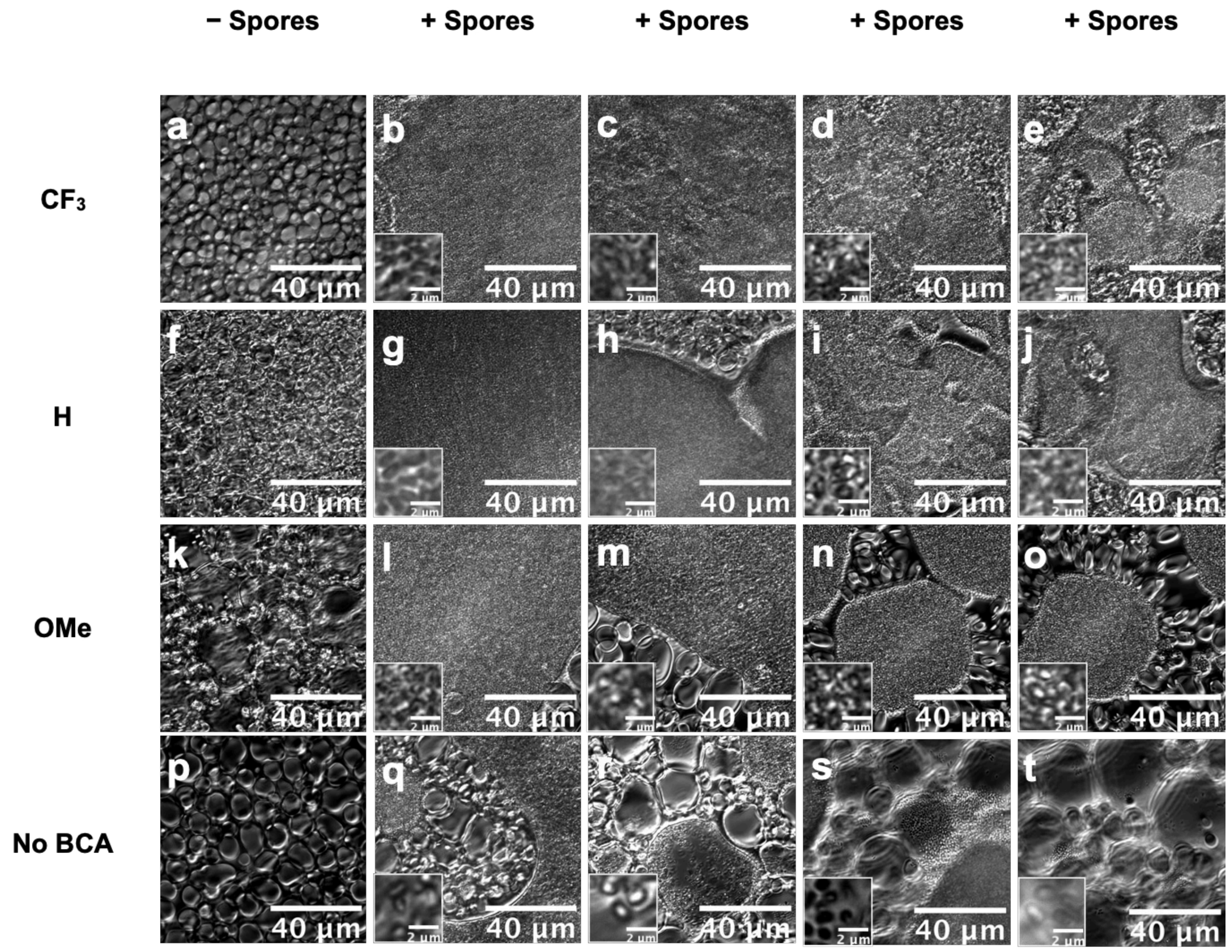

Figure S10.3. Optical microscopy images of organogel biocomposite precursors. Images show polymer-only DMSO solutions (**a, f, k, p**), and the addition of spores to effect near-homogeneous blending (**b**–**e**) with P4 (R_1 = CF_3), an intermediate level of polymer–spore interaction (**g**–**j**) with P7 (R_1 = H), significant separation of phases with P8 (R_1 = OMe), and a lack of polymer–spore interaction with P9 (0% BCA). Inset shows zoomed-in images of spores.

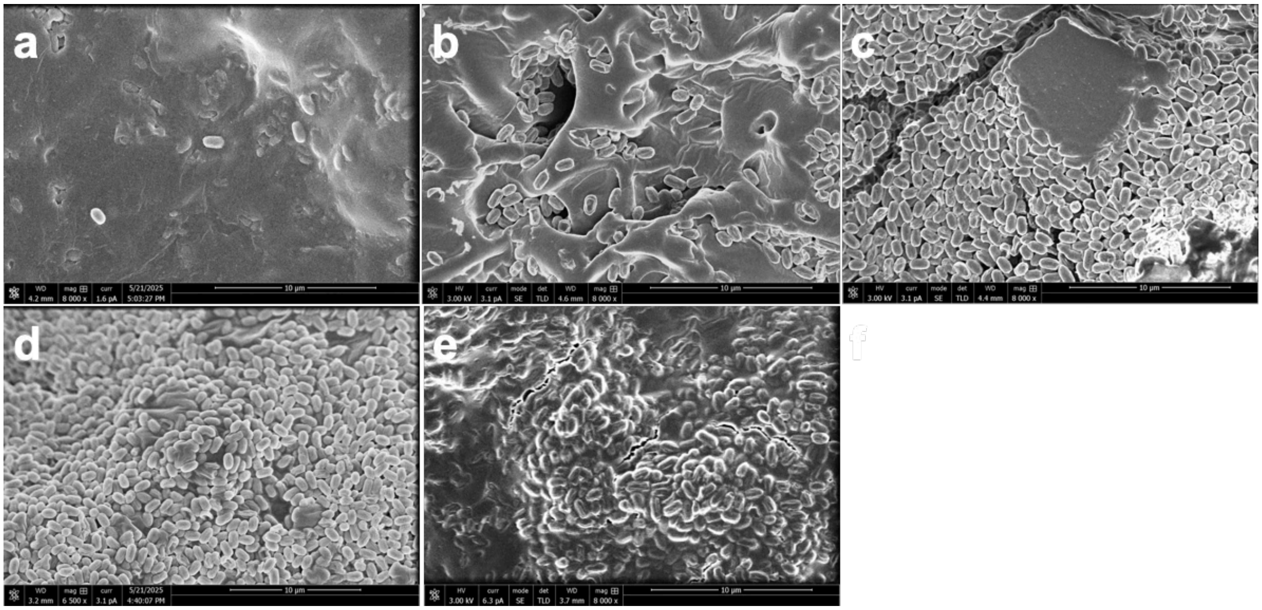

Figure S10.4. SEM images of dry biocomposites. Images of biocomposite materials made using P4 (**a**), P7 (**b**), P8 (**c**), P9 (**d**), and P11 (**e**).

S11. Material Disassembly and Analysis

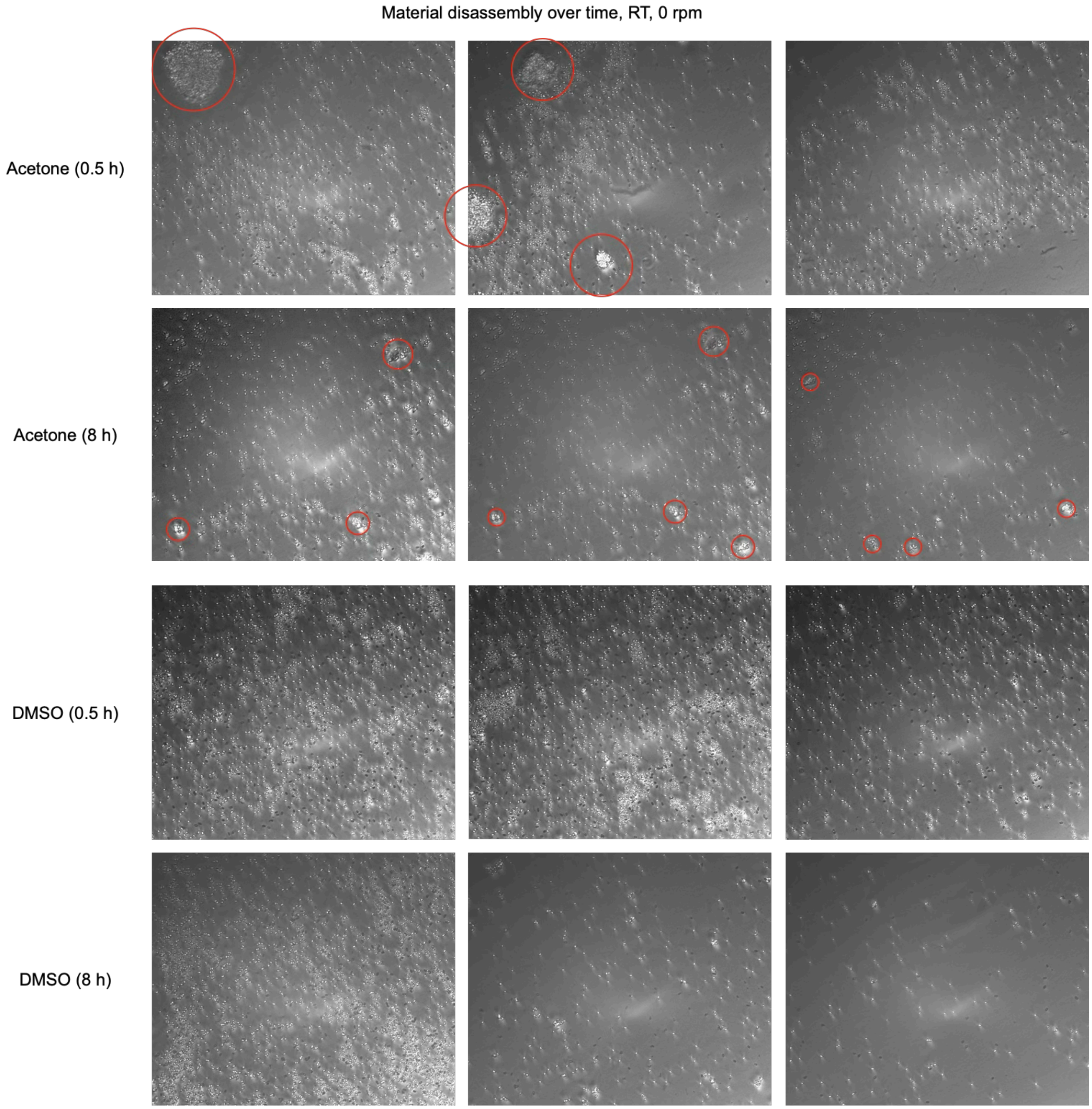

Figure S11.1. Polymer–spore disassembly in DMSO and acetone. Optical micrographs of supernatant during material disassembly. While disassembly in acetone shows residual aggregates after 30 minutes and smaller aggregates after 8 hours (red circles), disassembly in DMSO results in the quick disassembly and release of spores within the first 30 minutes.

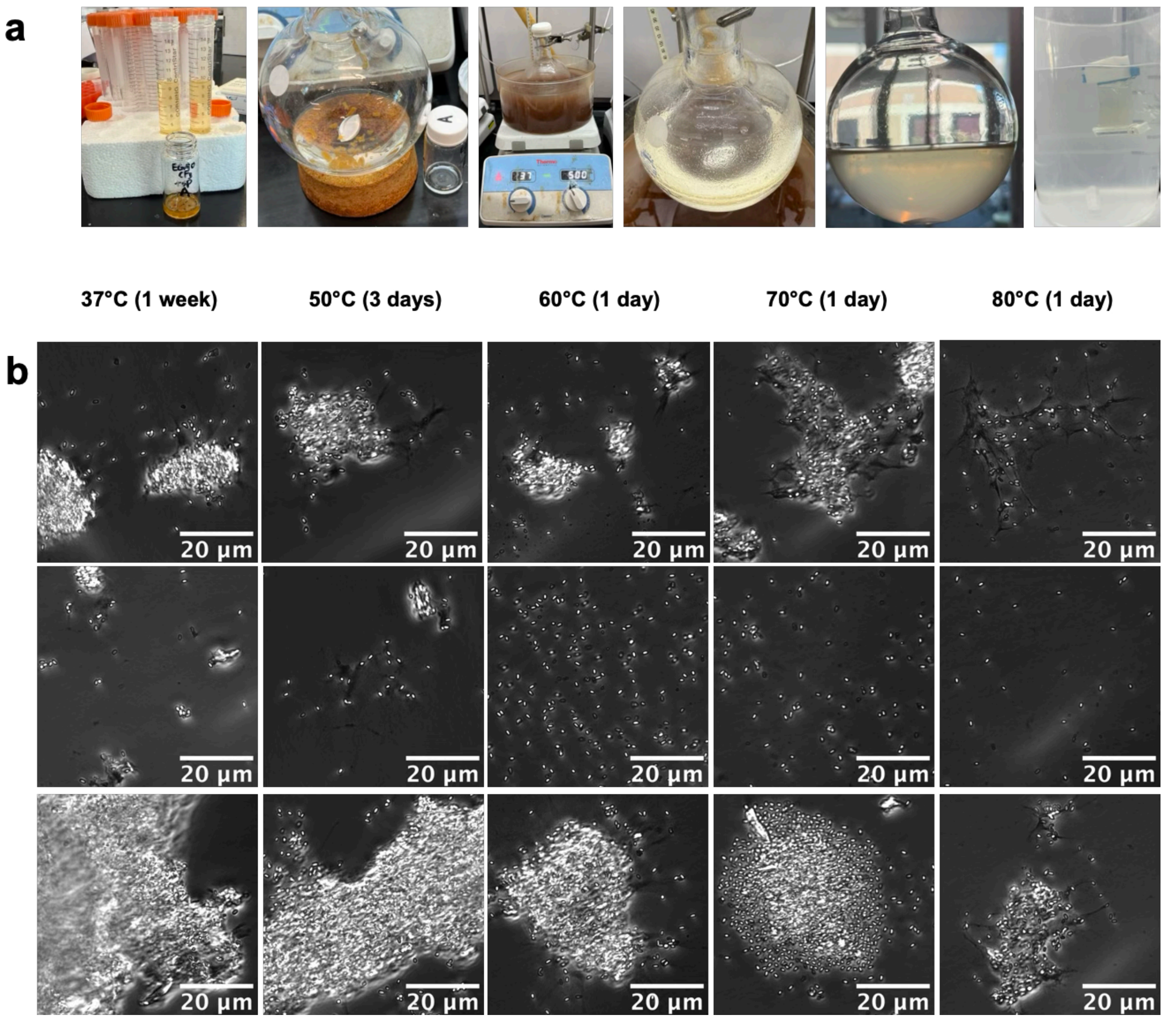

Figure S11.2. Dilution, heating, and stirring to encourage P4 material disassembly. (**a**) Resuspension of material fragments into a larger volume of DMSO to dilute down to approximately 0.2 mM of BCA, and subsequent heating (37–80 °C) and stirring (500–1000 rpm) to increase overall homogeneity. Polymer samples were dialyzed after centrifugation to remove spores. (**b**) Optical micrographs of polymer–spore assemblies after heating to different temperatures. Representative images (top row) show a gradual unraveling of the assemblies, free spore images (middle row) show an increasing prevalence of released spores, and large assembly images (bottom row) show decreasing sizes of assemblies at increased temperatures.

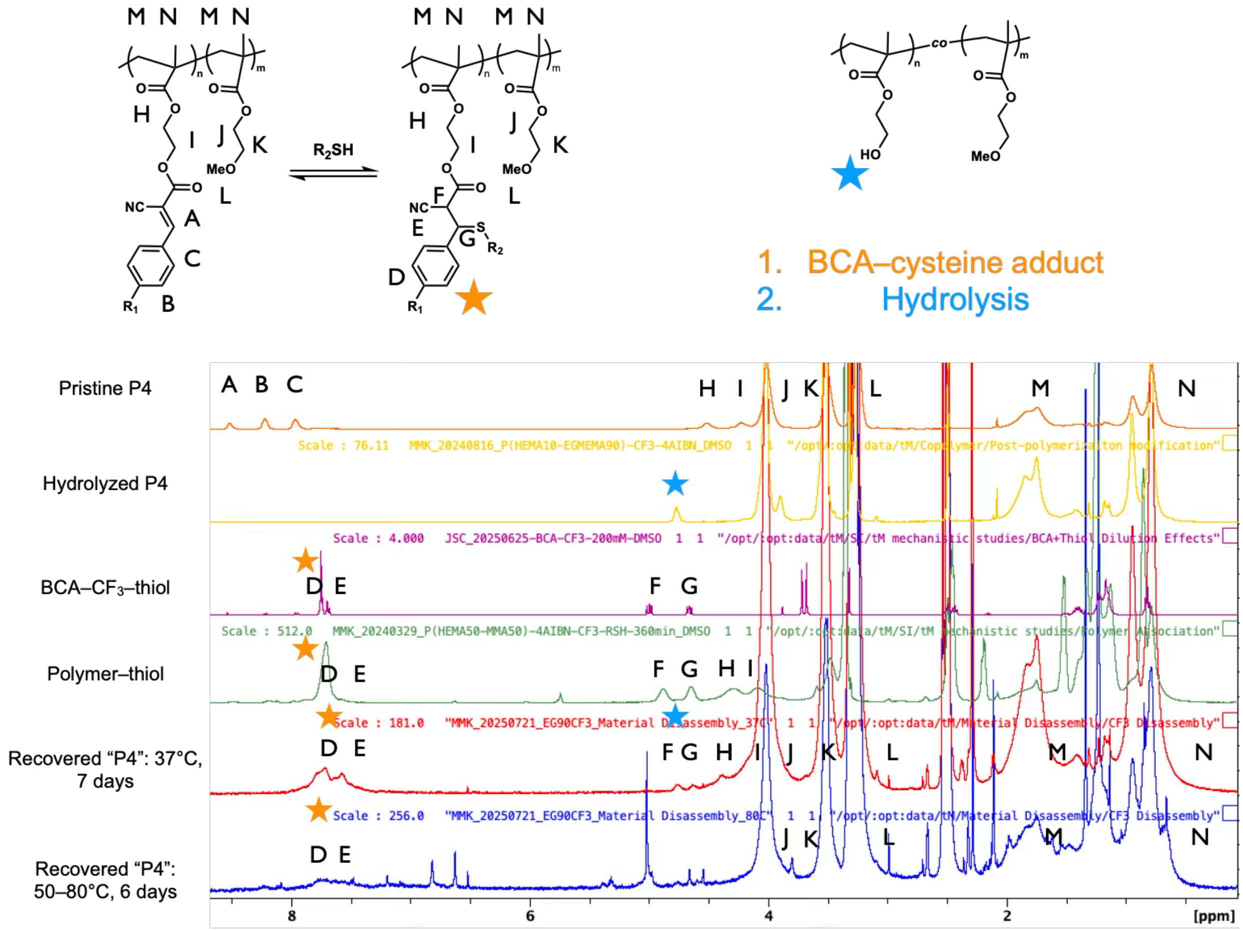

Figure S11.3. ^{1}H NMR spectrum of P4 polymers recovered from large-scale disassembly. Comparison of recovered "P4" polymers (bottom two spectra) with pristine P4 (top), a hydrolyzed variant of P4 (second from top, copolymer of HEMA and EGMEMA), and small molecule and polymer tM adducts of BCA–CF_3 (middle two). The recovered polymers lack the key BCA handles (peaks A–C) when compared to the pristine P4, with the EGMEMA-derived side chains still intact. Aromatic peaks appear that correspond to the tM adduct (orange star, peaks D and E), suggesting that polymers are still bound to cysteines residues. Because the intensity of the adduct peak is relatively weak compared to EGMEMA-derived peaks, polymer release could be dependent on the variable %BCA of each chain. Specifically, polymers with little to no BCA could come off the spore surface much easier than those with higher BCA composition. In addition, hydrolysis could result in the partial loss of BCA motif, as indicated by a peak that appears where the key OH peak (blue star) is expected, although it could belong to either the OH or the tM adduct. Overall, the high reactivity of BCA–CF_3 coupled with multivalent effects appears to have caused persistent tM bonding even with dilution, heating, and stirring.

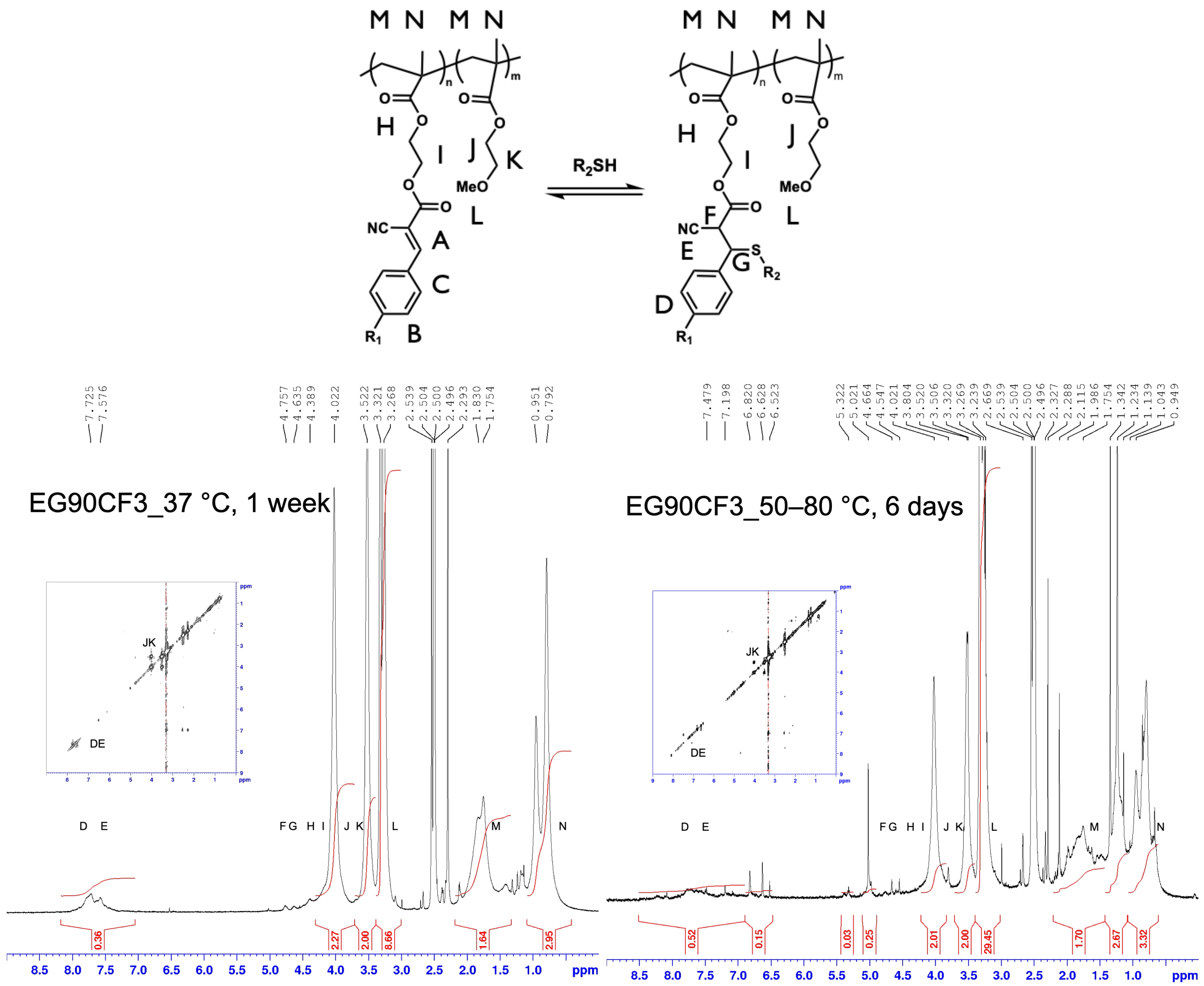

Figure S11.4. ^1H and ^1H–^1H NMR peak assignments of recovered P4 polymers.

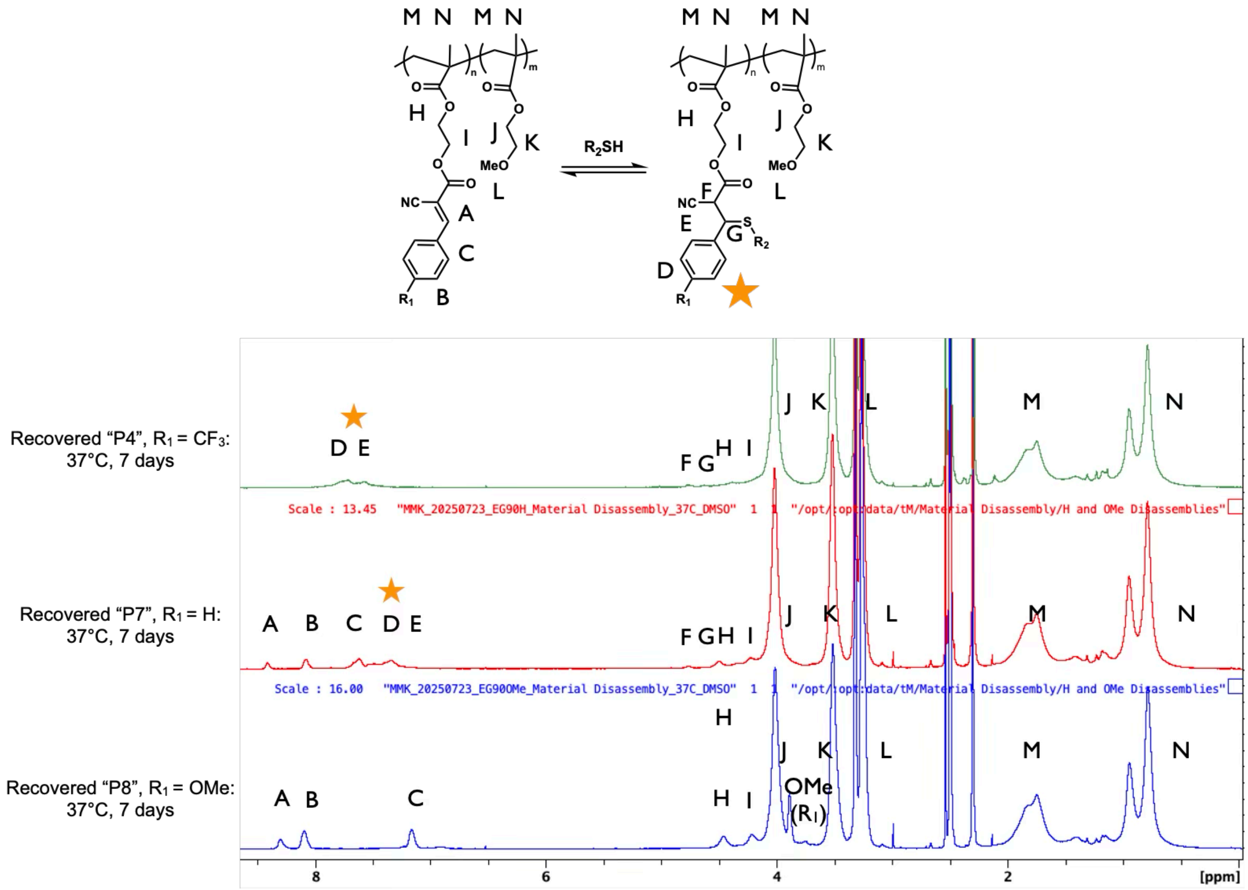

Figure S11.5. Relative ^{1}H NMR peak intensities of P4, P7, and P8 polymers recovered at 37 °C. Comparison of NMR spectra of polymers recovered from material disassembly at 37 °C reveals BCA electrophilicity-dependent polymer release. Specifically, peaks D and E (orange star) as well as peaks F and G correspond to characteristic aromatic, **α-**, and **β**-protons in the tM adduct.

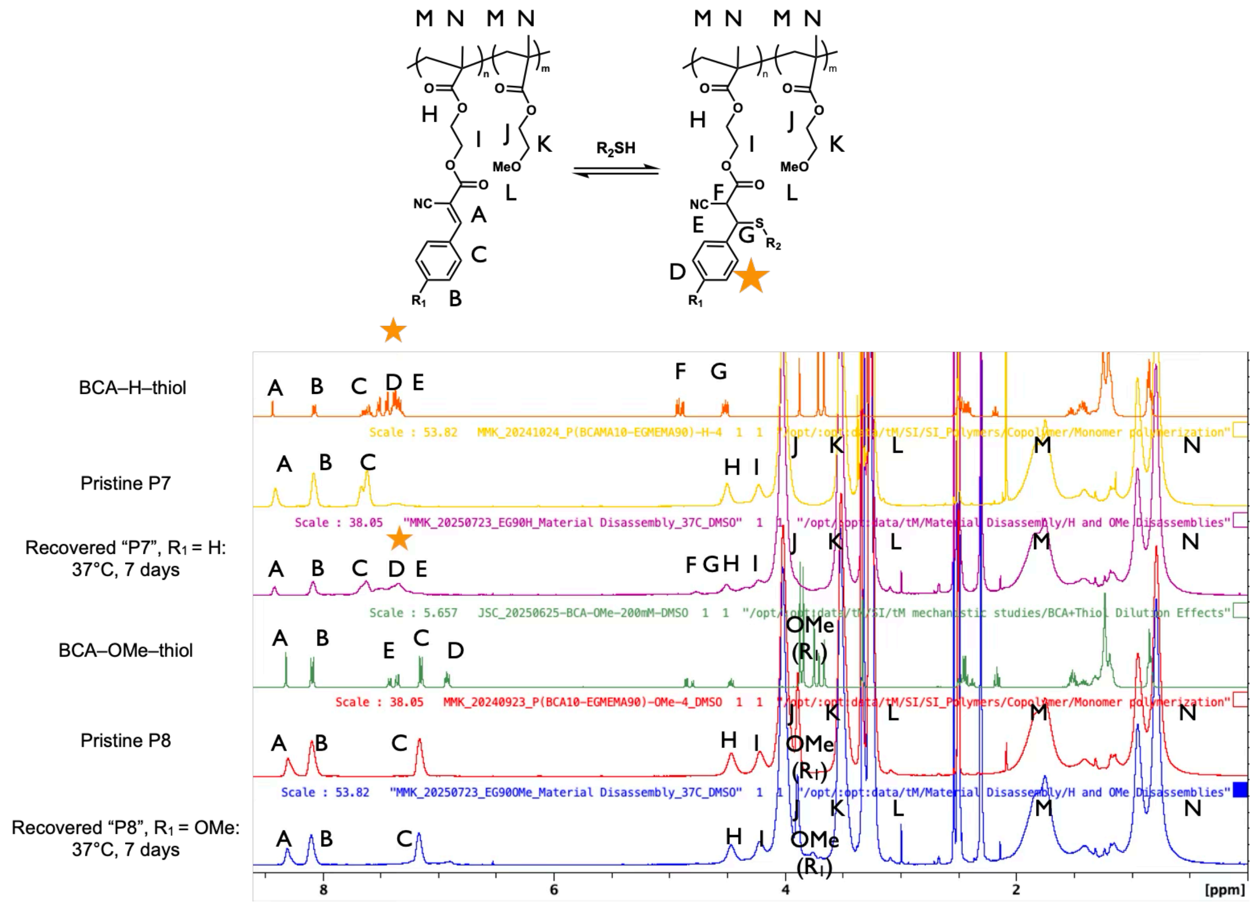

Figure S11.6. ^{1}H and ^{1}H–^{1}H NMR peak assignments of recovered P7 and P8 polymers. Comparison of NMR spectra of small molecule tM adducts, pristine polymer, and polymers recovered from material disassembly attempts. Similar to P4, recovered P7 shows some persistence of tM adducts (peaks D, E, F, and G) while recovered P8 is identical to pristine P8.

S12. Spore Viability After Organic Solvent Exposure

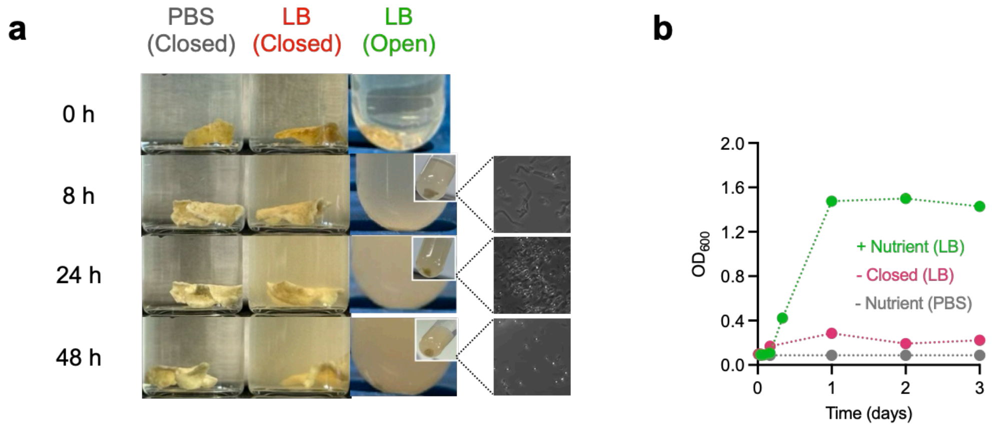

Figure S12. Spore germination and growth from the material surface. (**a**) Incubation of 1 mg of P4-based material fragments in capped vials with PBS and LB to limit oxygen exposure and in culture tubes to maintain aerobic conditions. Vials and tubes were shaken at 37 °C and 250 rpm. Inset shows that the fragment remaining intact during the spore germination and growth period and corresponding optical microscopy images shows the growth of cells and eventual sporulation. (**b**) Optical density (OD_{600}) measurements of each sample's supernatant over time that shows biocontainment (PBS) and partial growth (LB, closed) and full growth to saturation (LB, open). The requirement of both oxygen and nutrient, as well as the lack of turbidity without nutrient confirms the proposed surface-initiated germination mechanism.

S13. Catalytic Materials Assembled in Organic Solvent

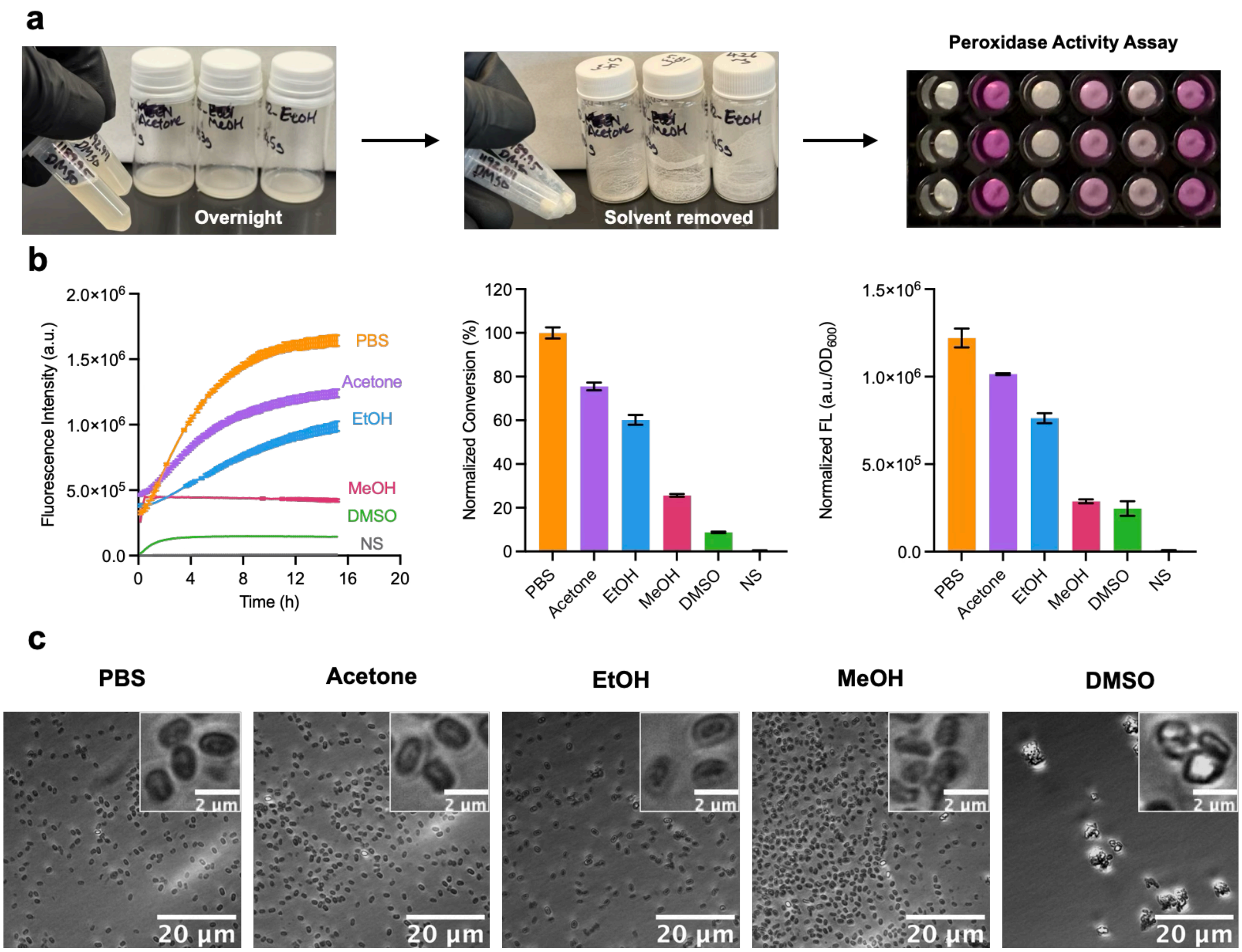

Figure S13.1. Catalytic activity of APEX2-displaying spores after incubation in organic solvents. (**a**) APEX2-displaying spores of the same batch were incubated in various organic solvents overnight, after which solvents were removed by evaporation under reduced pressure (MeOH, EtOH, and acetone) and by lyophilization (DMSO, MeOH, EtOH, and acetone). Spores were then resuspended in PBS at 1 mg/mL to monitor their relative catalytic efficiency by bulk fluorescence microscopy (λ_{ex} = 530 nm, λ_{em} = 590 nm). (**b**) The conversion of Amplex Red to resorufin over time using APEX2 spores that were incubated in various organic solvents. The endpoint (15 hours) was used to generate normalized conversions (fluorescence intensity relative to samples without exposure to organic solvent) and normalized fluorescence (fluorescence intensity divided by relative spore concentration, OD_{600}). (**c**) Optical micrographs of APEX2-displaying spores after incubation in organic solvent and resuspension in PBS.

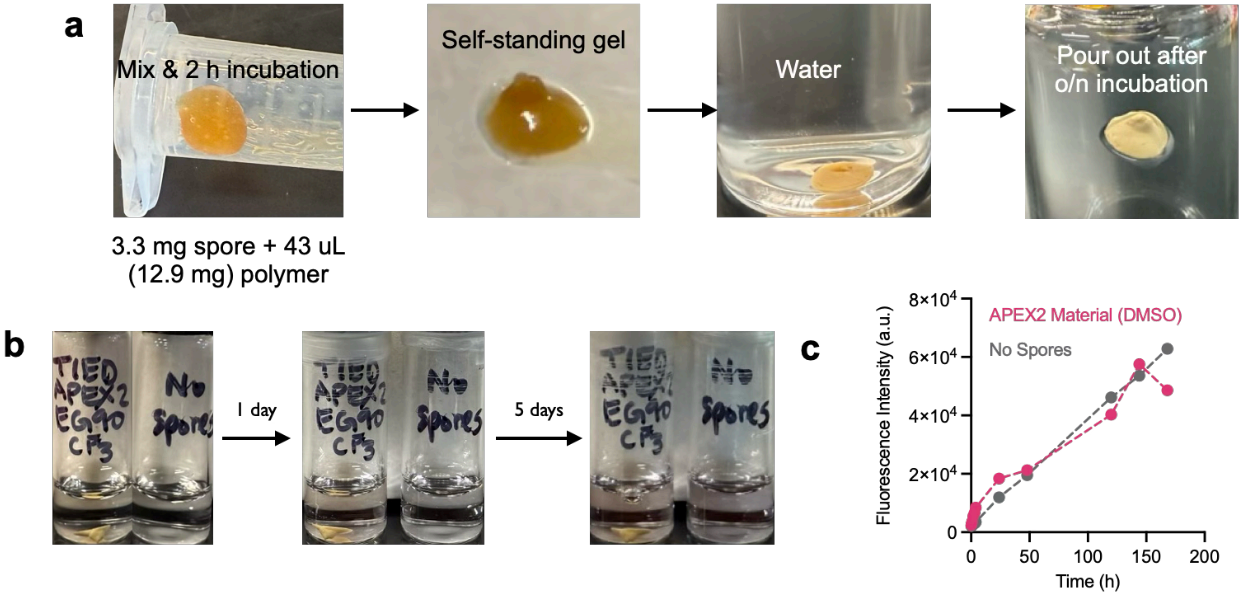

Figure S13.2. Catalytic activity of a material assembled in DMSO. (**a**) Incubation of dry spores with P4 in DMSO affords self-standing gels, which were then incubated in water overnight in preparation for the catalytic activity assay. (**b**) Images of vials containing 0.1 mM Amplex Red and 0.2 mM H_2O_2 with (left) or without (right) the APEX2 spore-containing material in PBS. (**c**) Fluorescence intensity measurements (λ_{ex} = 530 nm, λ_{em} = 590 nm) of the supernatant to compare the relative conversion rates of Amplex Red in the vials.

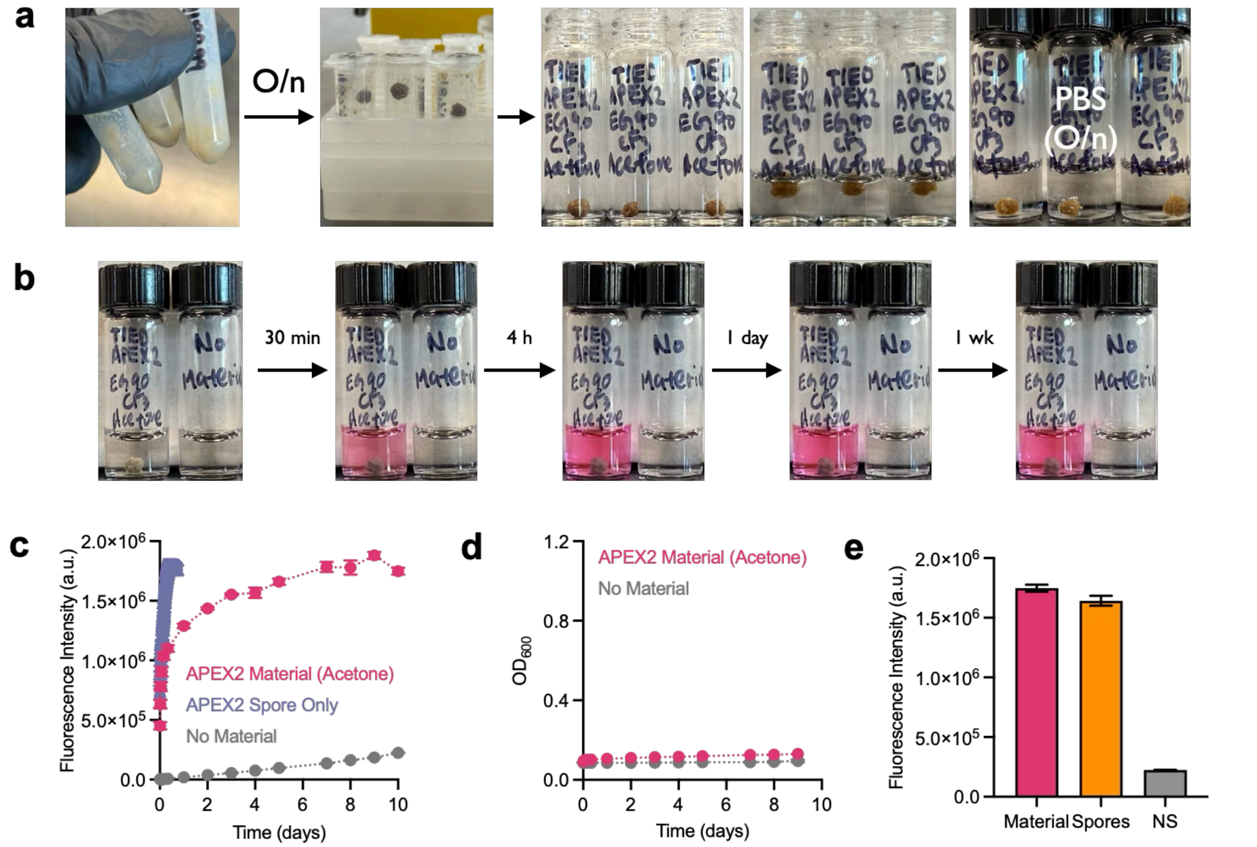

Figure S13.3. Catalytic activity of a material assembled in acetone. (**a**) Incubation of dry spores with P4 in acetone affords catalytic materials, which were then incubated in water overnight in preparation for the catalytic activity assay. (**b**) Images of vials containing 0.1 mM Amplex Red and 0.2 mM H_2O_2 with (left) or without (right) the APEX2 spore-containing material in PBS. (**c**) Fluorescence intensity measurements (λ_{ex} = 530 nm, λ_{em} = 590 nm) of the supernatant to compare the relative conversion rates of Amplex Red in the presence of the material, spores, and without spores. (**d**) Solution turbidity (OD_{600}) shows no spore leakage from the catalytic material. (**e**) The end-point fluorescence intensity of the material-containing reaction mixture compared to that of pristine APEX2 spores without organic solvent exposure, and to a mixture without spores.

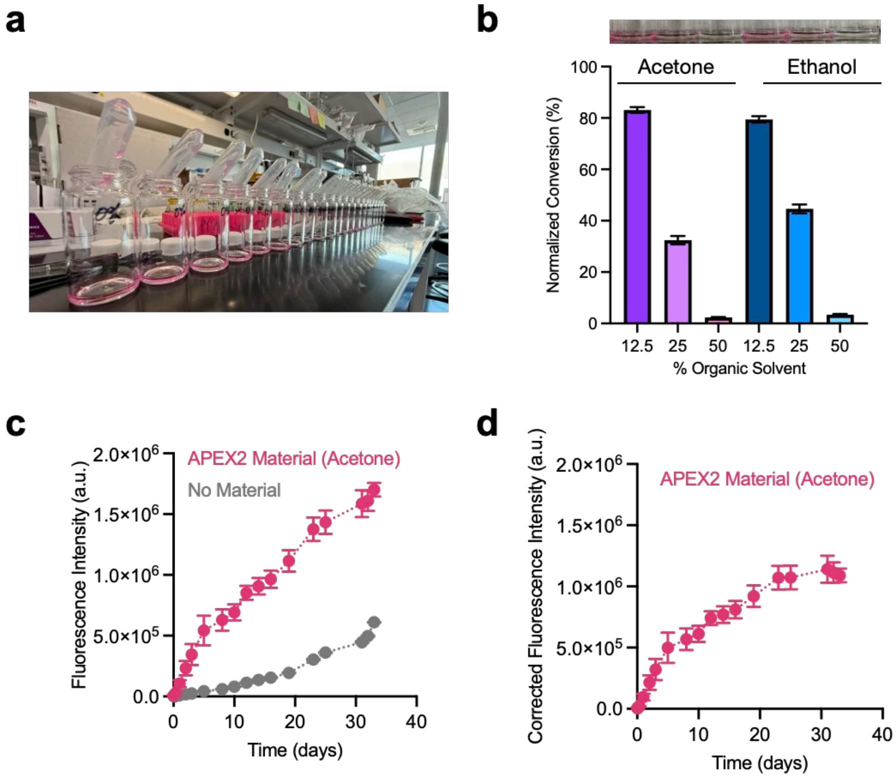

Figure S13.4. Catalytic activity in reaction mixtures containing organic solvent. (**a**) Collection of reaction mixture supernatant after Amplex Red peroxidation catalyzed by APEX2 spores. Tubes were shaken at 250 rpm and 37 °C for 48 h, solvent was removed, and the mixture was resuspended in water (to maintain similar PBS salt concentration). (**b**) Normalized reaction conversion (fluorescence intensity divided by that from a sample without organic solvent) in variable percentages of acetone or ethanol in PBS. (**c**) Fluorescence intensity time trace of the material-catalyzed peroxidation reaction. Uncatalyzed Amplex Red decomposition became significant after 10 days. (**d**) Baseline corrected fluorescence intensity that subtracts Amplex Red conversion from natural decomposition.

S14. Recovery and Renewal of Catalytic Spores

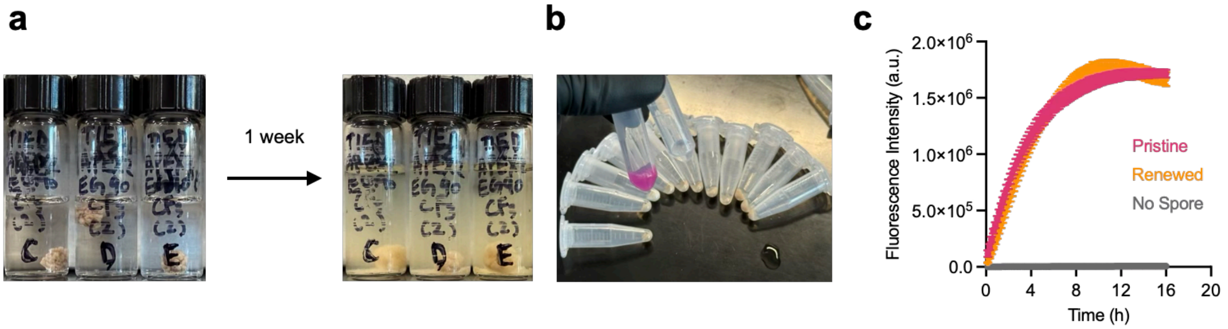

Figure S14. Disassembly of catalytic materials in acetone and subsequent recovery, germination, and growth of APEX2 spores. (**a**) Lyophilized catalytic materials incubated in acetone and shaken at 250 rpm and 37 °C for 1 week to effect partial disassembly. (**b**) Renewed spores that were obtained by germinating and growing APEX2 spores that were isolated from the supernatant of the partially disassembled materials by centrifugation. (**c**) Fluorescence intensity measurements that demonstrates the recovered activity of the renewed spores compared to pristine APEX2 spores and a reaction mixture without spores.

S15. Raman Spectroscopy Analysis

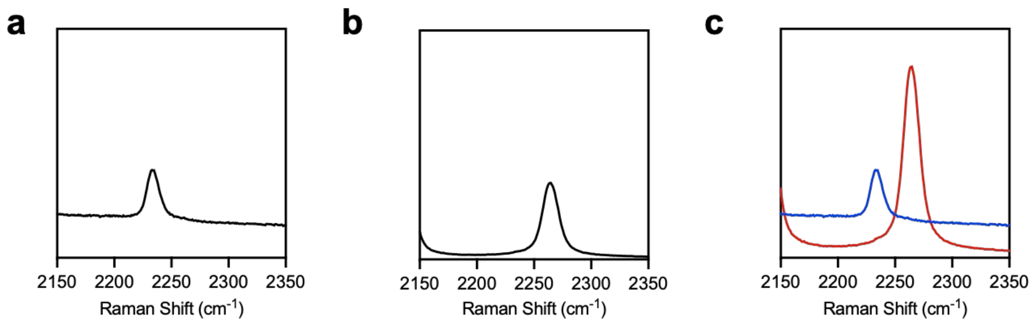

Figure S15.1. Raman shift of the BCA CN group. (**a**) BCA–CF_3 without exposure to thiols. (**b**) BCA–CF_3 with spores. (**c**) An overlay of BCA–CF_3 Raman spectra without (blue) and with (red) spores showing a blue shift from 2230 cm^{-1} to 2264 cm^{-1}.

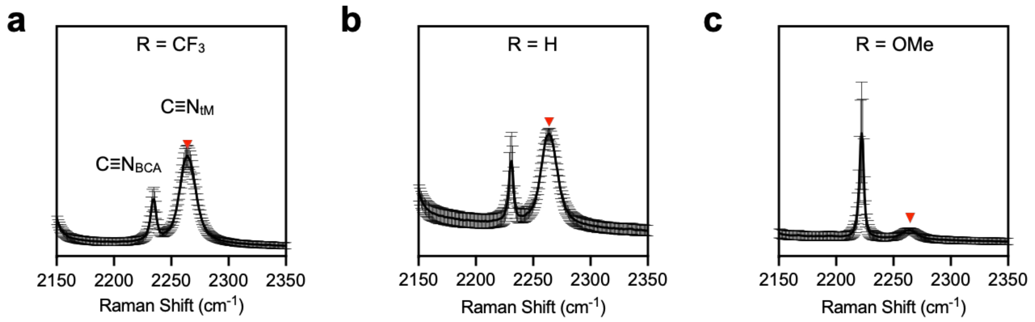

Figure S15.2. Raman spectra of BCA–spore mixtures. Spectra represent mixtures of spores with (**a**) BCA–CF_3, (**b**) BCA–H, and (**c**) BCA–OMe. Error bars represent standard error of the mean (n = 5).

S16. CFU Assay

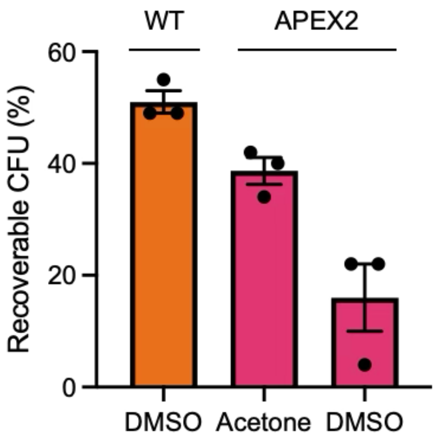

Figure S16. Spore viability after solvent exposure. Relative colony-forming units (CFU) of spores were determined following exposure to organic solvents under conditions mimicking material assembly. Wild-type (WT) spores and APEX2-displaying spores were incubated in DMSO overnight, pelleted, and subsequently incubated in ethanol for 2 h prior to CFU analysis. In a separate experiment, APEX2-displaying spores were incubated in acetone overnight. Data represent biological triplicates; each measured with technical replicates. Error bars represent standard error of the mean across biological triplicates (n = 3).

17. References

(1) Hui, Y.; Cui, Z.; Sim, S. Stress-Tolerant, Recyclable, and Renewable Biocatalyst Platform Enabled by Engineered Bacterial Spores. *ACS Synth. Biol.* **2022**, *11* (8), 2857–2868. https://doi.org/10.1021/acssynbio.2c00256.

(2) Dolinski, N. D.; Tao, R.; Boynton, N. R.; Kotula, A. P.; Lindberg, C. A.; Petersen, K. J.; Forster, A. M.; Rowan, S. J. Connecting Molecular Exchange Dynamics to Stress Relaxation in Phase-Separated Dynamic Covalent Networks. *ACS Macro Lett.* **2024**, *13* (2), 174–180. https://doi.org/10.1021/acsmacrolett.3c00717.

(3) Henn, D. M.; Fu, W.; Mei, S.; Li, C. Y.; Zhao, B. Temperature-Induced Shape Changing of Thermosensitive Binary Heterografted Linear Molecular Brushes between Extended Wormlike and Stable Globular Conformations. *Macromolecules* **2017**, *50* (4), 1645–1656. https://doi.org/10.1021/acs.macromol.7b00150.

(4) Hansen, C., M. The Three Dimensional Solubility Parameter and Solvent Diffusion Coefficient, Technical University of Denmark (Danish Technical Press), Copenhagen, 1967.
